# 7-Methylguanine With a Cyclopentane Backbone: A Bright Combination for a FIT-PNA RNA Sensor

**DOI:** 10.3389/bjbs.2025.15526

**Published:** 2025-11-21

**Authors:** Salam Maree, Pinaki Chanda, Sheethal Thomas Mannully, Hongchao Zheng, Daniel H. Appella, Eylon Yavin

**Affiliations:** 1 The Institute for Drug Research, The School of Pharmacy, The Faculty of Medicine, The Hebrew University of Jerusalem, Jerusalem, Israel; 2 Synthetic Bioactive Molecules Section, Laboratory of Bioorganic Chemistry (LBC), National Institute of Diabetes and Digestive and Kidney Diseases (NIDDK), National Institutes of Health, Bethesda, MD, United States

**Keywords:** FIT-PNA, BisQ, cpG^+^, molecular simulations, RNA biosensors

## Abstract

FIT-PNAs (forced intercalation-Peptide Nucleic Acids) are promising RNA sensors due to the enhanced fluorescence gained by such molecules upon RNA hybridization. In this report we describe a chemical approach that leads to unprecedented brightness for a FIT-PNA where the neighbouring Guanine base (G) to the fluorophore (a.k.a. surrogate base) is chemically modified with a cyclopentane (cp) backbone and is N-methylated, leading to a positively charged (G^+^) base. A series of G modified bases (G^+^, cpG, and cpG^+^) were introduced as the neighbouring base to BisQ (surrogate base) in 15-mer FIT-PNAs designed to sense the oncogenic long-noncoding RNA, colon cancer associated transcript 1 (lncRNA CCTA-1). Using synthetic RNA, the combination denoted as cpG^+^ led to a two-fold increase in brightness (BR = 16.9) compared to the unmodified G base (BR = 8.4). Introducing a G mismatch in RNA sequence that is opposite to the G base (G, G^+^, cpG, or cpG^+^) in the FIT-PNA, led to an increase in fluorescence that was not observed for synthetic DNA. Molecular simulations confirmed these observations and further correlated fluorescence data for FIT-PNAs with synthetic DNA and RNA with/out mismatches. Importantly, in ovarian cancer cells overexpressing CCAT1, only the cpG^+^ modified FIT-PNA produced a bright fluorescent signal, confirmed by FACS and confocal microscopy. Our results demonstrate that strategic chemical modifications of the neighboring G base in FIT-PNA significantly enhance their brightness and specificity for RNA detection in biological systems.

## Introduction

Peptide Nucleic Acids (PNAs) are synthetic DNA analogs that offer high chemical and enzymatic stability [1, 2], strong affinity, and sequence-specific recognition of complementary RNA and DNA [1, 3, 4].

PNAs face several limitations, including low aqueous solubility, a tendency to self-aggregate, non-specific interactions with biomacromolecules, poor cellular uptake, and rapid elimination *in vivo* [5]. To address these issues, researchers have explored various strategies such as chemical modifications of the PNA backbone [6], conjugation with cell-penetrating peptides [7] and targeting ligands [8], and encapsulation within nanoparticles [9, 10].

Detecting RNA biomarkers, such as pathogens (e.g., SARS-CoV-2, HIV) or disease indicator, is a simple and effective approach for medical diagnosis. Fluorogenic PNA probes [11–14] have proven particularly useful for detecting various RNA molecules, including mRNA, lncRNA, siRNA, and miRNA, both in extracted RNA sample [15, 16] and within cells [2, 17–22], tissues [23], and *in vivo* [17, 24].

PNAs have been applied for RNA sensing by various alternative approaches. One is based on Graphene Oxide (GO) that interacts with PNA by π−π stacking and quenches PNA fluorescence (of the appended fluorophore). In addition, due to its nanosize, GO facilitates PNA cellular uptake [25–28]. Upon release of PNA from GO after RNA hybridization, a fluorescent signal is gained.

A variety of electrochemical-based PNA sensors have been devised to detect miRNAs with miR-21 as the most common target for cancer diagnosis [29–33] as well as others [34–36]. Such biosensors are extremely sensitive to RNA levels reaching a limit of detection (LOD) in the range of femto to attomolar. To achieve such high sensitivity, other amplifications such as rolling cycle amplification (RCA) [37] and ATP-driven strand displacement of DNA nanoflowers [37, 38] was realized.

In addition, colorimetric detection of miR-21 [39] and c-Myc mRNA [40] was achieved with the PNA as the hybridization nucleic acid.

Initially developed with PNA chemistry [41], Forced-Intercalation (FIT) PNA probes have expanded to 2′-O-methyl RNA and DNA chemistries (FIT probes without a PNA backbone) [12, 24, 42, 43]. Incorporating Locked Nucleic Acid (LNA), a rigid sugar-modified nucleotide, flanking the FIT surrogate base Quinoline Blue (QB or Bis-Quinoline (BisQ) in PNA), significantly increases probe brightness [42]. Backbone modifications with a cyclopentane (cp) ring have also improved binding affinity and specificity to RNA and DNA [44–46]. Recently, we demonstrated that adding a cyclopentane-modified monomer (cpT or cpC) adjacent to BisQ, enhances brightness and quantum yield, especially when positioned 3′ to BisQ [47]. We further applied these modifications to detect a highly expressed long non-coding RNA (FLJ22447) in ovarian cancer cells [22]. The oncogenic long-noncoding RNA, colon cancer associated transcript 1 (lncRNA CCTA-1) is highly expressed in colorectal cancer (CRC) as determined by RT-qPCR [48, 49] and by an electrochemical Geno-sensing platform [50]. Based on a previous study on detecting CCAT1 in CRC [22], we selected this biomarker that is over-expressed in many cancers, among them, ovarian cancer.

Research from Aiba and Shoji showed that N-7 methylation of guanine (G^+^) improves hybridization efficiency and reduces PNA-PNA duplex formation [51]. Since the sensitivity of FIT-PNA depends on the ratio of duplex to single-stranded (ss) forms, lowering background fluorescence in ss form can improve biomarker detection. Background fluorescence arises partly from π-π interactions between BisQ and neighboring purines (G and A). The positively charged G^+^ may induce electrostatic repulsion, reducing this background.

To test this, we synthesized a series of FIT-PNAs with G^+^, cpG, and cpG^+^ modifications. We chose a 15-mer FIT-PNA targeting Colon Cancer Associated Transcript 1 (CCAT-1) [52–55], a lncRNA highly expressed in ovarian cancer [53, 56–59]. Our results show that all G-modified FIT-PNAs have similar background fluorescence, but the cpG^+^ variant produces the strongest fluorescent response upon hybridization to synthetic RNA. Importantly, cpG^+^ FIT-PNA effectively detects CCAT1 in ovarian cancer cells, where other variants show much less response.

## Materials and Methods

### Materials

Manual solid-phase synthesis was performed by using 5 mL polyethylene syringe reactors (Phenomenex, Torrance, CA, USA) that are equipped with a fritted disk. RNA oligos were purchased from IDT, USA. Fmoc-PNA monomers were purchased from PolyOrg, Inc. (USA) and used as received. Fmoc-D-Lysine and reagents for solid phase synthesis were purchased from Merck (Germany) and Biolab (Israel). Fmoc-protected cyclopentane PNA monomers (cpG) [60], positively charged guanine (G^+^) [51], and BisQ [61] were synthesized as previously reported.

### Solid-phase Synthesis of FIT-PNAs

FIT-PNAs were synthesized on solid phase in a continuous process, thereby eliminating the need for repurification [61]. Coupling of the first monomer, Fmoc-D-Lysine(tBOC)-OH, onto Novasyn TGA Resin was performed as follows: The resin (100 mg, 0.25 mmol/g) was allowed to swell in 2 mL DMF for 2 h. For pre-activation, 5 equivalents of diisopropylcarbodiimide (DIC, 0.125 mmols, 15.8 mg, 19.5 µL), and 0.1 equivalent of 4-dimethylaminopyrimidine (DMAP, 0.0025 mmols, 0.3 mg) were added to a solution of 10 equivalents of Fmoc-D-Lysine(tBOC)-OH (0.25 mmols, 117 mg) in DCM (2.5 mL) in an ice bath. After 20 min, the mixture was evaporated, re-dissolved in dry DMF and added to the resin. After 5h, the resin was washed with dichloromethane (5 × 2 mL), DMF (5 × 2 mL) and the procedure was repeated. Fmoc deprotection was performed by treating the resin with 20% piperidine in DMF for 10 min (×2), followed by washing with DCM (5 × 2 mL) and DMF (5 × 2 mL). For a 10 μmols scale synthesis on TGA-NovaSyn resin (loading−0.25 mmol/g), 2-(1H-7-azabenzotriazol-1-yl)-1,1,3,3-tetramethyl uronium hexafluorophosphate methanaminium (HATU, 40 μmols, 15.2 mg), hydroxybenzotrilazole (HOBT, 40 μmols, 5.4 mg), diisopropylethylamine (DIPEA, 80 μmols, 14 µL), and Fmoc-amino acids/Fmoc-PNA monomers (40 μmols) were mixed in dry DMF (0.4 mL). After 5 min of pre-activation, the solution was transferred to the resin. After 60 min, the reaction mixture was discarded, and the resin was washed with DCM (5 × 2 mL) and DMF (5 × 2 mL). The PNA−peptide conjugates were deprotected and released from the resin by treatment with 90:10 (v/v) TFA/*m*-cresol for 2 h (2 × 1 mL). The PNAs were triturated with cold diethyl ether, and the precipitate was collected by centrifugation and decantation of the supernatant. The residues were dissolved in water and purified by semi preparative HPLC using a Dionex UltiMate 3000 HPLC system (ThermoFisher Scientific, Waltham, MA, USA) with automatic fraction collection. A semi-preparative C18 reversed-phase column (Jupiter C18, 10u, 300Å, 250 × 10 mm, Phenomenex) was used with a linear gradient of eluents A (0.1% TFA in water) and B (MeCN) at a flow rate of 4 mL/min. Mass analysis of FIT-PNAs was acquired by MALDI-TOF MS (Bruker Daltonics, Microflex LRF) using 2,5-Dihydroxybenzoic acid (DHB) as a matrix.

### T_m_ Measurement

The melting temperatures (T_m_) of the PNA: RNA/DNA duplexes were determined using UV melting curves recorded on an Evolution One Plus UV-Vis Spectrophotometer. Solutions of the FIT-PNAs and their complementary RNAs (1:1 ratio) were prepared in a PBS buffer (100 mM NaCl, 10 mM NaH_2_PO_4_, pH 7) and adjusted to a final duplex concentration of 2 µM. Prior to analysis, the samples were heated from 20 °C to 90 °C at a rate of 5 °C/min and then cooled back to the starting temperature at a rate of 2 °C/min. Absorbance at 260 nm was monitored as the temperature increased to 90 °C at a rate of 1 °C/min. Each measurement was repeated at least twice, with the T_m_ value representing the average value of the inflection point.

### Fluorescence Measurements

Fluorescence spectra were recorded by using a Jasco FT-6500 spectrometer. Measurements were carried out in fluorescence quartz cuvettes (10 mm). Solution of the FIT-PNA and the RNA/DNA (ratio 1:2) were prepared in a PBS buffer (pH 7.0) at 37 °C for 2 h.

### UV-Vis Spectrum

UV-Vis spectra of CCAT1 FIT-PNAs were recorded using an Evolution One Plus UV-Vis Spectrophotometer. FIT-PNA solutions, either with or without the presence of RNA synthetic RNA, were prepared in PBS buffer (100 mM NaCl, 10 mM NaH_2_PO_4_, pH 7). Prior to measurement, the FIT-PNA:RNA duplex solutions were annealed at 37 °C for 2 h. Full spectrum was recorded in the range of 200–800 nm.

### Circular Dichroism (CD) Spectroscopy

CD spectra were acquired using a Jasco F-1100 spectropolarimeter equipped with a temperature-controlled sample holder. Samples included both single-stranded FIT-PNA and FIT-PNA:RNA duplexes (1:1 M ratio), prepared at a final FIT-PNA concentration of 15 µM in PBS buffer (100 mM NaCl, 10 mM NaH_2_PO_4_, pH 7.0). Hybridization was carried out by incubating the samples at 37 °C for 2 h. CD measurements were performed at 25 °C using a 1 mm pathlength quartz cuvette with a total volume of 200 µL. Spectra were recorded over 200–320 nm range, and each final spectrum represents an average of five replicates.

### Quantum Yields

Quantum yields for all FIT-PNAs were calculated using Cresyl Violet as a reference fluorescent dye [62–64]. Each FIT-PNA (4, 6 and 8 µM) was hybridized to complementary and G-mismatched RNA in PBS (pH 7.0) at a 1:2 ratio, respectively, and incubated at 37 °C for 2 h. The samples were excited at 580 nm, and emission spectra were recorded between 400 and 750 nm.

### Limit of Detection

Limit of detection (LOD) of all FIT-PNAs was recorded by using a Cytation 3 plate reader. Measurements were carried out in Greiner 96 well black plates with flat bottom in a Tris-EDTA buffered solution (25 mM Tris-EDTA, 150 mM NaCl with 0.05% Tween-20). The FIT-PNA’s concentration was constant in the duplex solution (0.5 µM) while the RNA was added in different concentrations. All the FIT-PNAs were incubated with the complementary RNA at 37 °C for 2 h on the plate for annealing. LOD was calculated according to the formula: LOD = 3.3*σ/slope [65].

### RT-qPCR

Total RNA from the cells was isolated using TRIzol reagent (ThermoFisher Scientific, Waltham, USA) following the manufacturer’s instructions and quantified using a NanoDrop 2000 Spectrophotometer (ThermoFisher Scientific, Waltham, USA). Reverse transcription of RNA(1 µg) into cDNA was performed using the QScript cDNA Synthesis Kit (Quantabio, Beverly, MA, USA) according to the manufacturer’s instructions. RT-qPCR was conducted to on a CFX Connect Real-Time PCR Detection System (BioRad, Hercules, CA, USA) using PerfeCTa SYBER® Green FastMix qPCR reagent (Quantabio, Beverly, MA, USA). The primers used are described in Supplementary Material (Supplementary Table S4), they were purchased form IDT (Coralville, USA) and HyLabs (Rehovot, Israel). The target genes were amplified under the following thermocycling conditions: initial denaturation at 95 °C for 5 min, followed by 40 cycles of 95 °C for 10 s and 60 °C for 30 s. The specificity of the PCR products was verified by analyzing the melting curves. The relative expression of target genes was calculated using the 2^−ΔΔCT^ method, and expression levels were normalized to the housekeeping gene Ribosomal protein lateral stalk subunit P0 (RPLP0).

### Cell Culture

OVCA433 and SKOV3 cells were grown in EMEM and McCoy’s 5A, respectively, (Beit Haemek Biological Industries, Israel) supplemented with 10% (v/v) FBS, 100 U/mL penicillin; 0.1 mg/mL streptomycin; 2 mM L-Glutamine, at 37 °C with 5% CO_2_. Cells were routinely checked for *mycoplasma* contamination using MycoBlue *Mycoplasma* detector Kit (Vazyme, China).

### Flow Cytometry Analysis

FACS analysis of FIT-PNA uptake was conducted by seeding OVCA433 (50 × 10^4^) and SKOV3 (35 × 10^4^) cells into 6-well plates, allowing them to adhere overnight under standard culture conditions until they reached 70%–80% confluence. The medium was replaced, and the cells were incubated with 2 µM FIT-PNAs at 37 °C in a humidified atmosphere containing 5% CO_2_ for 5 h. Following thorough washing, the cells were harvested using 0.25% Trypsin-EDTA (3 min at 37 °C), collected into 15 mL Falcon tubes, and centrifuged at 1,200 rpm for 5 min. The supernatant was discarded, and the cells were resuspended in 350 μL cold PBS, which was then filtered through 70 µm Falcon Cell Strainers. The samples were analyzed using a Fortessa FACS analyzer (Core Research Facilities, The Hebrew University of Jerusalem, Jerusalem, Israel). The cells were gated based on normalized fluorescence of untreated cells to determine the percentage of cells that internalized the FIT-PNAs. Data analysis was performed using FlowJo 10.10 software.

### Statistical Analysis

FACS data are presented as the mean ± SD from experiments. At least two independent experiments were performed per assay, each with Two technical replicates. Statistical significance was determined using a One-way or Two-way ANOVA test with P < 0.001 considered extremely significant (***), P < 0.01 highly significant (**), and P < 0.05 statistically significant (*). mRNA expression, as measured by RT-qPCR, was normalized to the control cell expression, and the data represent the average of two biological replicates, each with corresponding duplicates. Statistical analysis was carried out using Student’s t-test, with P < 0.05 considered statistically significant (*).

### Confocal Microscopy

Twenty-four hours prior to PNA addition, OVCA433 cells (60 × 10^3^) and SKOV3 cells (50 × 10^3^) were seeded onto µ-slide 8-well chambers (ibidi GmbH, Gräfelfing, Germany) and incubated at 37 °C with 5% CO_2_ until reaching 60%–70% confluence. The cells were rinsed with 1× PBS and treated with 2 µM FIT-PNAs in medium at 37 °C for 5 h. After incubation, the cells were washed twice with 1× PBS and stained with Hoechst (1 μg/mL) for 15 min at room temperature. The cells were then washed again with 1× PBS, and 300 µL of 1× PBS was added to each well for live cell observation. Control cells included OVCA433 and SKOV3 cells that were not treated with FIT-PNA. Cell fluorescence observations were performed using a Nikon AIR+ confocal microscope (Core Research Facilities, The Hebrew University of Jerusalem, Israel) and images were analyzed using NIS-Elements AR software (version 5.21).

### Molecular Stimulations

Double-stranded PNA molecules were constructed using the Proto Nucleic Acid Builder (pNAB) software, where the 5′ to 3′ sequence of the target RNA/DNA strand was used as the N- to C-terminal input for PNA strand generation. The resulting PNA:PNA duplex structures were analyzed using the x3DNA server to obtain helical parameters. The generated parameter file was manually edited (replacing “T” with “U”) and used to model corresponding RNA:RNA and B-form DNA:DNA duplexes using x3DNA. Relevant single strands from the pNAB-generated PNA:PNA duplex and the x3DNA-generated RNA:RNA or DNA:DNA duplex were extracted and saved as individual PDB files. These were then docked into PNA:RNA and PNA:DNA duplexes using the HNADOCK server. The resulting duplex structures were processed in Schrödinger Maestro (v.14.0) for structure preparation. Final structures were energy-minimized using the OPLS4 force field implemented in Maestro. Further details are provided in the Supplementary Material.

## Results

### Chemical Synthesis of cpG^+^ and FIT-PNAs

**SCHEME 1:**
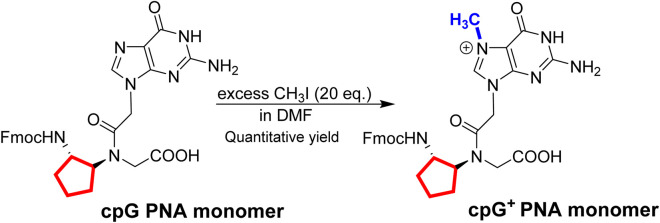
One step synthesis of cpG^+^ PNA monomer. In red – cp backbone and in blue - methyl group on N7 guanine.

Based on the simple one-step synthetic of G^+^ PNA monomer [51], we prepared in quantitative yields the cpG^+^ PNA monomer starting from the Fmoc-protected cpG monomer [60] (Scheme 1). The final product was used after a simple workup and was fully characterized by NMR and HRMS (Supplementary Figures S35, S36).

**SCHEME 2:**
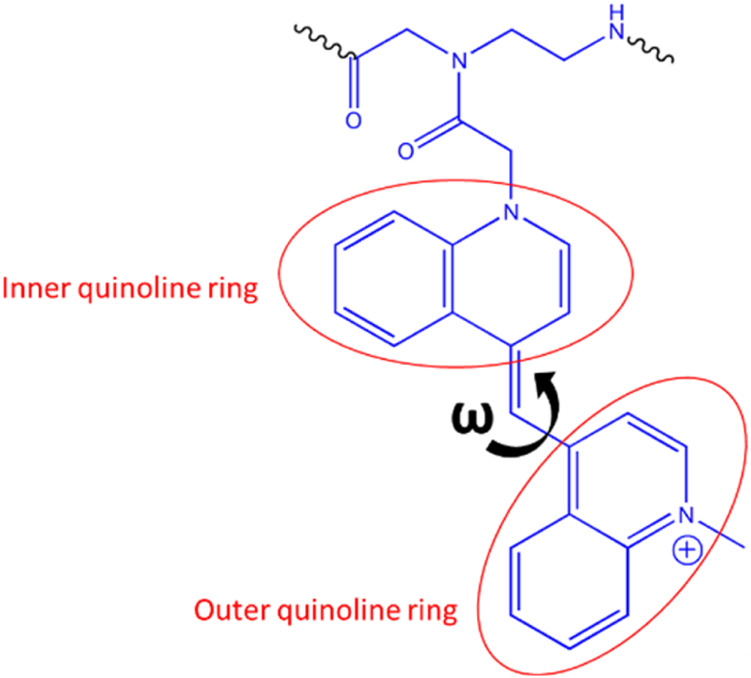
Quinoline rings in BisQ.

In this study, we have synthesized a series of G-modified FIT-PNAs (Table 1) that target the lncRNA CCAT1. This RNA biomarker has been previously studied in our lab for FIT-PNA based diagnosis in colorectal cancer where CCAT1 was detected in unfixed cancer cell lines [18] and in fresh human cancer tissues [23]. The choice of the BisQ surrogate base (Scheme 2) was for several reasons: (1) ease of synthesis [61]; (2) superior RNA sensing in comparison to the TO surrogate base [61]; and (3) red-shifted emission (λ_em,max_ = 613 nm) that is more suitable for biological samples (lower background fluorescence from biological samples). Three types of G modifications adjacent to BisQ were installed: G^+^, cpG, and cpG^+^. FIT-PNAs were synthesized on the solid support (Novasyn TGA resin) using standard Fmoc-based peptide/PNA Chemistry. To provide water solubility and cellular uptake, FIT-PNAs were installed with a short peptide ((D)K_4_) that has higher stability in biological medium than the L-peptide (K_4_), as previously reported [2]. After FIT-PNA cleavage from the solid support, the FIT-PNA oligomers were purified by HPLC and analyzed by MALDI-TOF MS (Supplementary Figures S1–S4).

**TABLE 1:** G-modified and unmodified FIT-PNAs. BisQ is marked in blue and guanine (modified and unmodified) PNA bases are marked in red. (D)K_4_ = 4 Lysines in D configuration.

Entry	Description	PNA sequence
Unmodified	Unmodified FIT-PNA (control)	^3′^(D)K_4_-GTGAAT G - BisQ -TCCAACC^-5‘^
G^+^	G^+^ modified FIT-PNA	^3′^(D)K_4_-GTGAAT ${\text{G}}^{+}$ - BisQ -TCCAACC^-5‘^
cpG	cpG modified FIT-PNA	^3′^(D)K_4_-GTGAAT cpG - BisQ -TCCAACC^-5‘^
cpG^+^	cpG^+^ modified FIT-PNA	^3′^(D)K_4_-GTGAAT ${\text{cpG}}^{+}$ - BisQ -TCCAACC^-5‘^

### Photophysical and Molecular Simulation Studies of FIT-PNAs With Synthetic RNA and DNA

**FIGURE 1:**
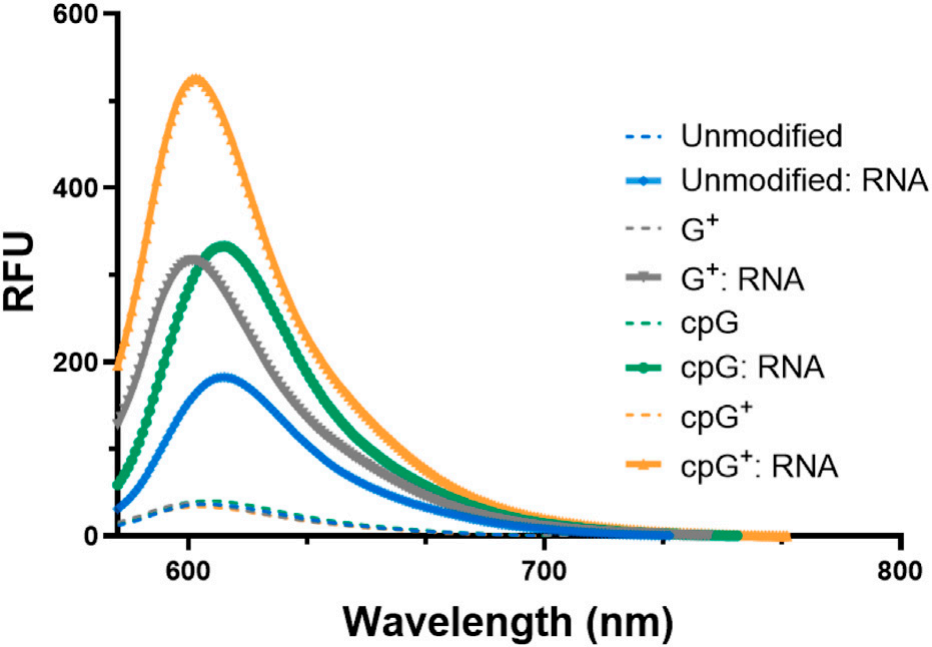
Enhanced fluorescence of G modified and unmodified CCAT1 FIT-PNAs after RNA hybridization. Annealing was conducted by incubating FIT-PNA:RNA at 37 °C for 2 h. The unmodified FIT-PNA is marked in blue, G^+^ FIT-PNA is marked gray, cpG FIT-PNA is marked in green and cpG^+^ is marked in orange. [FIT-PNA] = 0.5 µM, [RNA] = 1 µM. (λex = 570 nm, λem = 580 nm). RFU = Relative Fluorescence Unit.

FIT-PNAs were annealed to a fully complementary 15-mer RNA, and the fluorescence of the duplexes was measured (Figure 1). Among the sequences tested, cpG^+^ FIT-PNA exhibited the most pronounced response, showing over a twofold increase in fluorescence compared to the unmodified G FIT-PNA. While single modifications on G (G^+^ and cpG) also enhanced fluorescence, their performance was less effective than the double modification. Overall, the data demonstrate that the combined chemical modifications on G synergistically improve the fluorescence response, making cpG^+^ FIT-PNA the most responsive RNA probe.

We next explored the sequence selectivity of FIT-PNAs by measuring the fluorescence of FIT-PNAs with RNA sequences that have a single mismatch at the nucleobase opposite to the modified G base in the FIT-PNA sequence. To our surprise, we found higher emission for all FIT-PNA sequences for the GG mismatch in RNA (Figure 3A; Supplementary Figure S16). This was not the case for a GG mismatch in DNA (Figure 3C). All other mismatches were well-discriminated by FIT-PNAs (Supplementary Figure S16 for RNA; Supplementary Figure S17 for DNA).

The overall photophysical properties of FIT-PNAs were assessed by measuring three key parameters: brightness (BR = QY × ε_max_), fluorescence increases upon RNA hybridization (I/I_0_), and quantum yield (QY) (Table 2). These measurements also included the GG mismatch RNA for all FIT-PNAs.

**TABLE 2:** Photophysical properties and binding affinities of FIT-PNAs. BR, brightness; ɸ, quantum yields; I/I_0_, signal to background ratio, and LOD, limit of detection.

Entry	PNA:RNA duplex	λ_max,abs_ _[nm]_	ε_max_ _[mM-1 cm-1]_	**ɸ**	BR _[mM-1 cm-1]_	I/I_0_	T_m_	ΔT_m_	LOD _[nM]_
Unmodified	G-C	588	93.3	0.09	8.4	4	65.8	-	5
	G-G_mm_	590	97.5	0.11	10.4	6.6	59.3	(−6.5)	5.65
G^+^	G-C	584	85.2	0.11	6.4	5.3	62.8	(−3.0)	2.67
	G-G_mm_	584	93.2	0.13	12.1	7	58.9	(−6.9)	3.92
cpG	G-C	590	86.5	0.17	14.7	8.7	68.9	(**+3.1**)	2
	G-G_mm_	588	92.6	0.24	22.2	13.7	61.2	(−4.6)	3.5
cpG^+^	G-C	584	89	**0.19**	16.9	10.5	60.9	(−4.9)	**1.56**
	G-G_mm_	584	95.8	0.29	**27.8**	14.3	56.9	(−8.9)	2.6

As shown in Table 2, G^+^ FIT-PNA exhibited parameters similar to those of the unmodified G FIT-PNA, indicating minimal improvement in photophysical performance. For example, QY values were 0.11 and 0.13 for fully matched (FM) and GG mismatch RNA, respectively, comparable to 0.09 and 0.11 for G FIT-PNA. In contrast, cpG FIT-PNA demonstrated increased responsiveness, with QYs of 0.17 and 0.24 for FM and GG mismatch RNA. Most notably, the cpG^+^ FIT-PNA achieved the best results, with approximately a threefold increase in both QY and brightness (QY = 0.29; BR = 27.8) with GG mismatch RNA compared to G FIT-PNA. Its fluorescence enhancement over background in the single-stranded form was also significant, with I/I_0_ values of 10.5 and 14.3 for FM and GG mismatch RNA, respectively.

**FIGURE 2:**
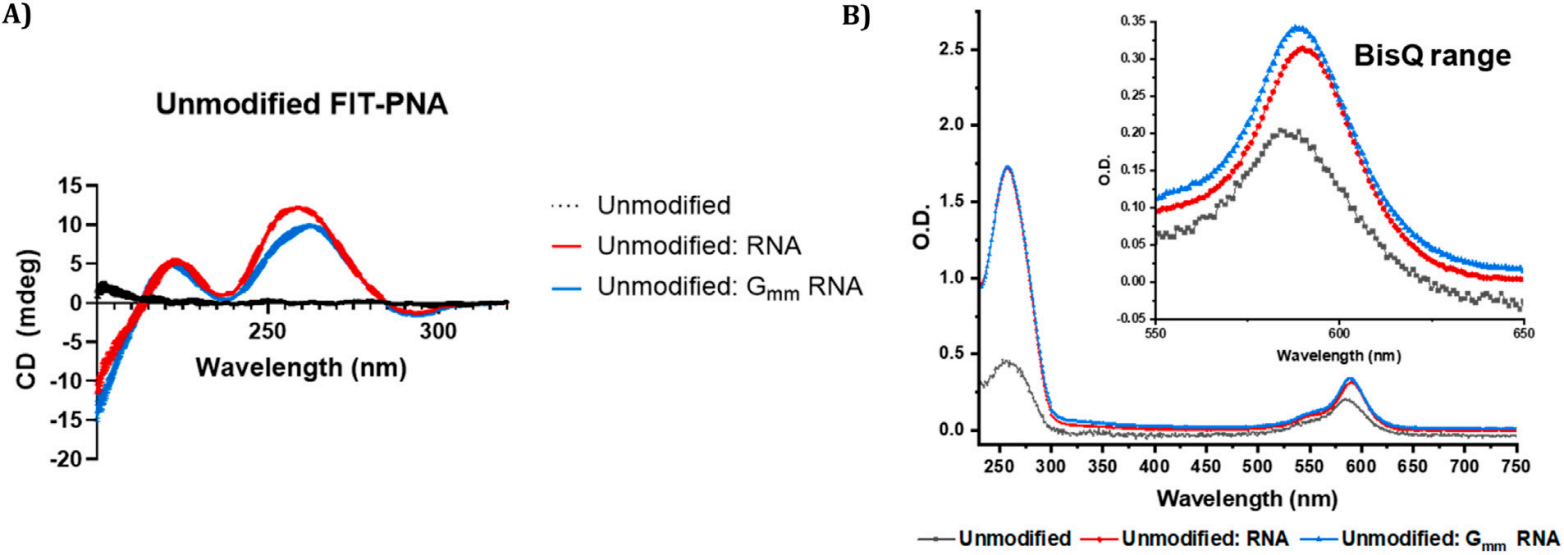
CD and UV-Vis spectra of unmodified CCAT1 FIT-PNA before and after RNA hybridization. Annealing was conducted by incubating FIT-PNA:RNA at 37 °C for 2 h **(A)** CD spectra of unmodified CCAT1 FIT-PNA as single strand (marked in black) and hybridized to fully matched and G_mm_ RNA (marked in red and blue, respectively) in PBS buffer (100 mM NaCl, 10 mM NaH_2_PO_4_, pH 7.0). [FIT-PNA] = [RNA] = 15 µM. **(B)** UV-Vis spectrum of unmodified FIT-PNA as single strand and hybridized to fully matched and G_mm_ RNA in PBS buffer. [FIT-PNA] = [RNA] = 4 µM.

Moreover, a slight increase in absorbance values was observed in the BisQ absorbance region (λ_max,abs=_ ∼590 nm) for the duplex formed with G_mm_ RNA compared to the fully matched RNA duplex (Figure 2B; Supplementary Figures S18–S20). This was observed for all FIT-PANs (modified and unmodified).

Limit of detection (LOD) is defined as the lowest concentration of RNA detected by a particular probe. All modified FIT-PNAs exhibited a lower LOD compared to the unmodified G FIT-PNA (Supplementary Figure S26; Table 2). Specifically, the LOD values for fully matched RNA decreased from 5.00 nM for the unmodified probe to 2.67, 2.00, and 1.56 nM for G^+^, cpG, and cpG^+^ FIT-PNAs, respectively. cpG^+^ FIT-PNA also showed the lowest LOD with G_mm_ RNA (2.6 nM). However, all FIT-PNAs exhibited higher values for the LODs (inferior) for the G_mm_ RNA compared to the fully matched RNA.

**FIGURE 3:**
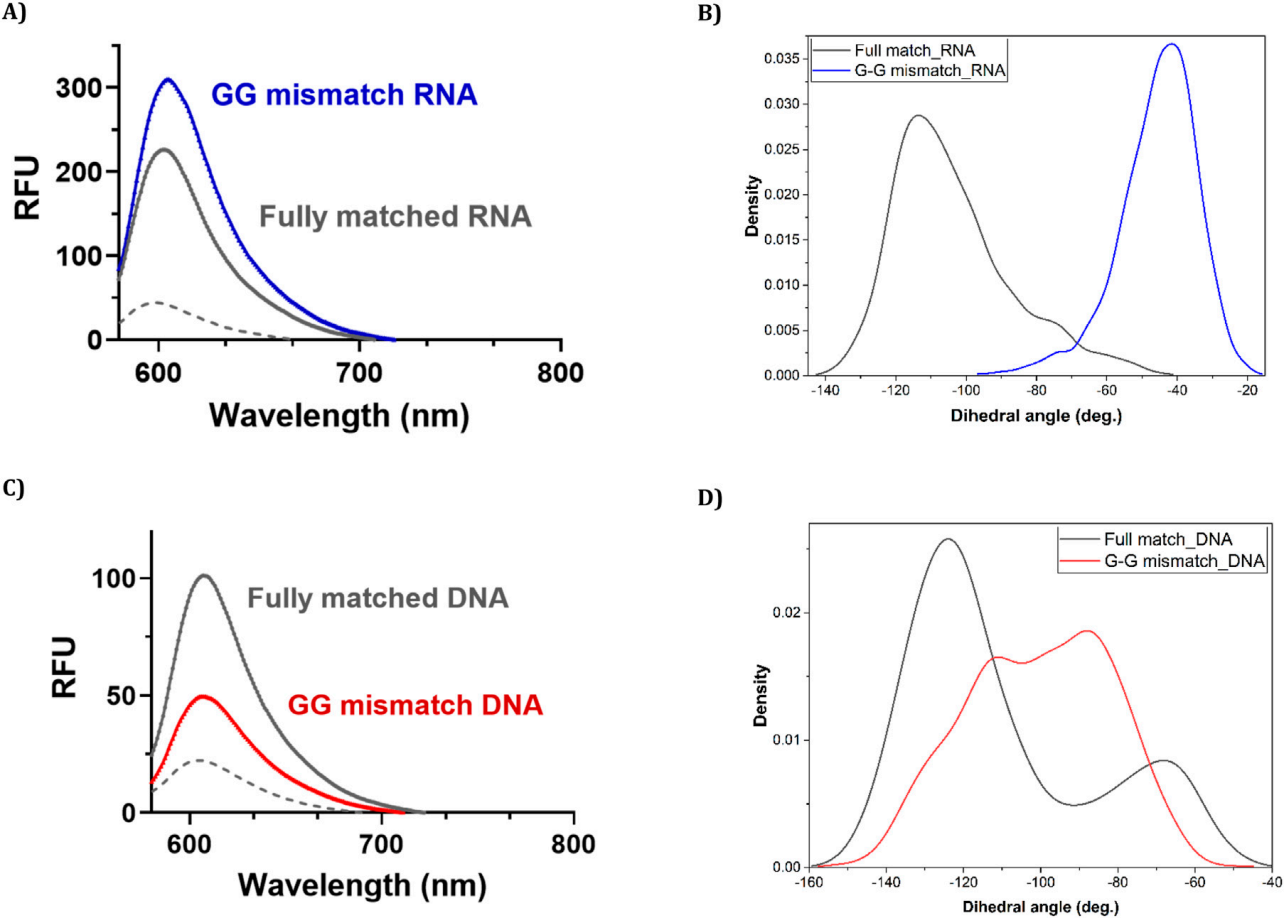
Fluorescence measurements and their accompanying molecular simulations for duplex formation of unmodified (G) CCAT1 FIT-PNA with FM and GG mismatched RNA and DNA. Annealing was conducted by incubating FIT-PNA:RNA/DNA at 37 °C for 2 h [FIT-PNA] = 0.5 µM, [RNA] = [DNA] = 1 µM. RFU = Relative Fluorescence Unit. **(A)** Enhanced fluorescence of unmodified FIT-PNA after hybridization to the GG mismatch RNA sequence in comparison to the fully matched (FM) RNA sequence. **(B)** Population density of different values of the dihedral angle (ω) in 10 ns of the simulation for “fully matched BisQ FIT-PNA:RNA duplex” (black line) and “G-G mismatched BisQ FIT-PNA:RNA duplex” (blue line). **(C)** Enhanced fluorescence of unmodified FIT-PNA after hybridization to the GG mismatch DNA sequence in comparison to the fully matched (FM) DNA sequence. **(D)** Population density of different values of the dihedral angle (ω) in 10 ns of the simulation for “fully matched BisQ FIT-PNA:DNA duplex’ (black line) and “G-G mismatched BisQ FIT-PNA:DNA duplex” (red line).

We also measured melting temperatures (T_m_) for FIT-PNAs with FM and GG mismatch RNA (Table 2; Supplementary Figures S8–S11). For FM RNA, the presence of a positive charge on G (G^+^) generally decreased duplex stability, shown by a T_m_ reduction of about 3 °C for G^+^/G and around 4.9 °C for cpG^+^/cpG. Conversely, the cpG modification increased T_m_ value only for cpG FIT-PNA. All FIT-PNAs exhibited lower T_m_ values with GG mismatch RNA, indicating decreased duplex stability. Interestingly, there was an inverse correlation: the lower stability of the GG mismatch duplex corresponded with higher fluorescence intensity across all FIT-PNAs. FIT-PNAs were also tested with synthetic DNA, where no fluorescence increase was observed for GG mismatches, and all mismatches were well-resolved (Supplementary Figure S17; Supplementary Table S3), in accordance with molecular simulations (Figures 3C,D, 5).

CD spectroscopy was also performed on all FIT-PNA sequences in the presence and absence of complementary RNA and G_mm_ RNA (Figure 2A; Supplementary Figure S21) to investigate molecular interactions and assess the structural stability of the formed duplexes. As expected for single-stranded FIT-PNAs, including the cpG and cpG^+^ modified variants, no detectable CD signals were observed [66]. Upon hybridization, both FIT-PNA:RNA and FIT-PNA:G_mm_ RNA duplexes exhibited characteristic CD signatures of antiparallel PNA:RNA heteroduplexes [3, 67]. The CD signals of the FIT-PNA:G_mm_ RNA duplexes were less intense in the ∼260–270 nm region, and a slight spectral shift was observed, suggesting altered helical organization and reduced duplex stability. Nonetheless, both duplex types displayed a similar maximum at ∼210–220 nm and a minimum at ∼240–245 nm. These observations align with the thermal melting (T_m_) data, where FIT-PNAs showed lower T_m_ values when hybridized to G_mm_ RNA compared to the fully matched RNA.

To provide some insight into these observations, we modelled the structures of “fully matched BisQ FIT-PNA:RNA duplex,” “G-G mismatched BisQ FIT-PNA:RNA duplex,” “fully matched BisQ FIT-PNA:DNA duplex,” and “G-G mismatched BisQ FIT-PNA:DNA duplex” (detailed in ESI, Supplementary Figures S37–S41). It is noteworthy that these molecular simulations were done at the ground state of these molecules.

Ten ns stochastic dynamics simulations were conducted to monitor the dihedral angle (ω) between the two quinoline rings of BisQ (Scheme 2) and their π-π stacking interactions with neighboring nucleobases.

We observed that in the fully matched duplex (with RNA), ω mainly ranged from −100° to −140°, with dominantly the inner quinoline ring π-stacking effectively, and the outer ring exhibited weak or no π-stacking at all. In contrast, the G-G mismatched duplex (with RNA), predominantly showed ω between −40° and −80°, with both rings forming face-to-face π-π interactions. This may explain the higher fluorescence observed for a G-G mismatch in RNA. Analysis of dihedral angle populations (ω) during 10 ns shows a clear difference between the duplex types (Figures 3B,D). For the G-G mismatch with RNA, more population density lies between −40° and −80°, favorable for π–π stacking of both quinoline rings (Figure 3B, blue trace). In the fully matched duplex (with RNA), ω predominantly falls between −100° and −140°, a range unsuitable for stacking of the outer quinoline ring (Figure 3B, black trace). For DNA, the population densities are strikingly different (Figure 3D). For the G-G mismatch with DNA, ω spreads all over (Figure 3D, red trace) with no distinct population density at the −40° to −80° range. With FM (with DNA), there is a distinct population at this range (Figure 3D, black trace), albeit lower than that of G-G mismatch with RNA (Figure 3B, blue trace). Altogether, the results shown in Figures 3B,D correlate with the spectroscopic data (Figures 3A,C).

To validate our observation that the value of ω ranging from −40° to −80° is suitable for π−stacking of both quinoline rings in BisQ, we further modelled and simulated a total of 4 FIT-PNAs that include X-BisQ in each probe (where X = A, G, C, or T, Supplementary Figures S1, S5–S7), and performed a correlation study between the percentage of well-stacked population of BisQ (where both quinoline rings are stacked between neighboring bases) and the percentage of population where ω ranges from −40° to −80° over 10 ns (Supplementary Figures S41a). We obtained a 0.82 Pearson’s correlation coefficient (Supplementary Figures S41b). Subsequently, we validated this correlation to the experimental value of fluorescence against π-stacked BisQ population which provided a Pearson’s correlation coefficient of ca. 0.74 (Supplementary Figures S41c). Statistically, these two values suggest a good correlation between these parameters (fluorescence and π-stacked BisQ population).

**FIGURE 4:**
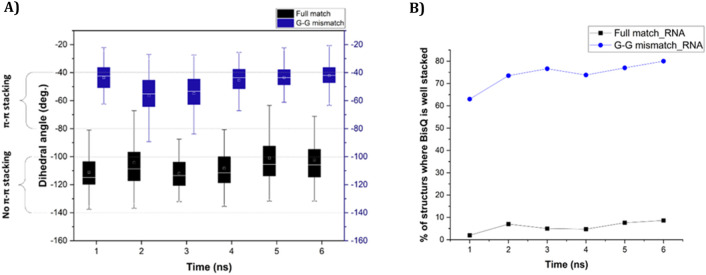
**(A)** Change of dihedral angle (ω) over time in case of “fully matched BisQ FIT-PNA:RNA duplex” (black boxes) and “G-G mismatched BisQ FIT-PNA:RNA duplex” (blue boxes). Data is shown for initial 6 ns. **(B)** Percentage of stacked population of BisQ over 6 ns. “Fully matched BisQ FIT-PNA:RNA duplex” is represented in black trace and “G-G mismatched BisQ FIT-PNA:RNA duplex” is represented in blue trace.

**FIGURE 5:**
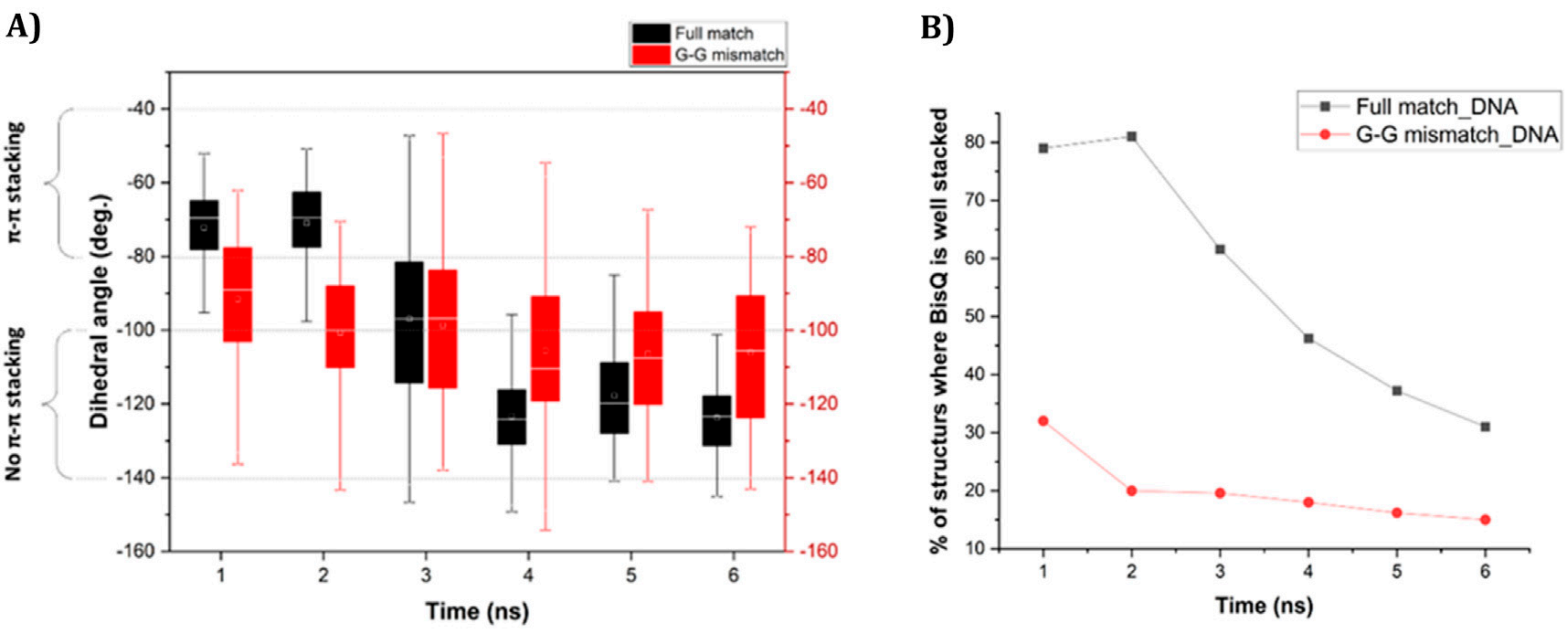
**(A)** Change of dihedral angle (ω) over time in case of “fully matched BisQ FIT-PNA:DNA duplex” (black boxes) and “G-G mismatched BisQ FIT-PNA:DNA duplex” (red boxes). Data is shown for initial 6 ns. **(B)** Percentage of stacked population of BisQ over 6 ns. “Fully matched BisQ FIT-PNA:RNA duplex” is represented in black trace and “G-G mismatched BisQ FIT-PNA:RNA duplex” is represented in red trace.

We next studied the dynamics of ω over each nanosecond (for 6 ns, data is shown in Figures 4, 5), focusing on either poor π-stacking (−100° < ω < −140°) or appreciable π-stacking (−40° < ω < −80°). For RNA, ω is quite stable for both FM and GG mismatch (Figure 4A). The well-stacked structures of BisQ for GG mismatch consist of 60%–80% of all structures generated during this timeframe (Figure 4B, blue trace). In contrast, for FM RNA, this value drops down to 1%–10% (Figure 4B, black trace).

For DNA, ω is much more dynamic in this timeframe (Figure 5A, 6 ns). For FM BisQ FIT-PNA:DNA duplex (Figure 5A, black boxes for each 1 ns of simulation), the outer quinoline ring of BisQ is initially well stacked in the duplex (ca. 80% of all structures during the first 2 ns) but gradually drops to ca. 30% after 4 ns (Figure 5B, black trace). In contrast, the well-stacked structures for GG mismatch DNA (Figure 5A, red boxes for each 1 ns of simulation) consist of only ca. 30% and decrease to ca. 18% during the remaining 5 ns of the simulation (Figure 5B red trace).

### Detection of CCAT1 FIT-PNA in Ovarian Cancer (OC) Cells

**FIGURE 6:**
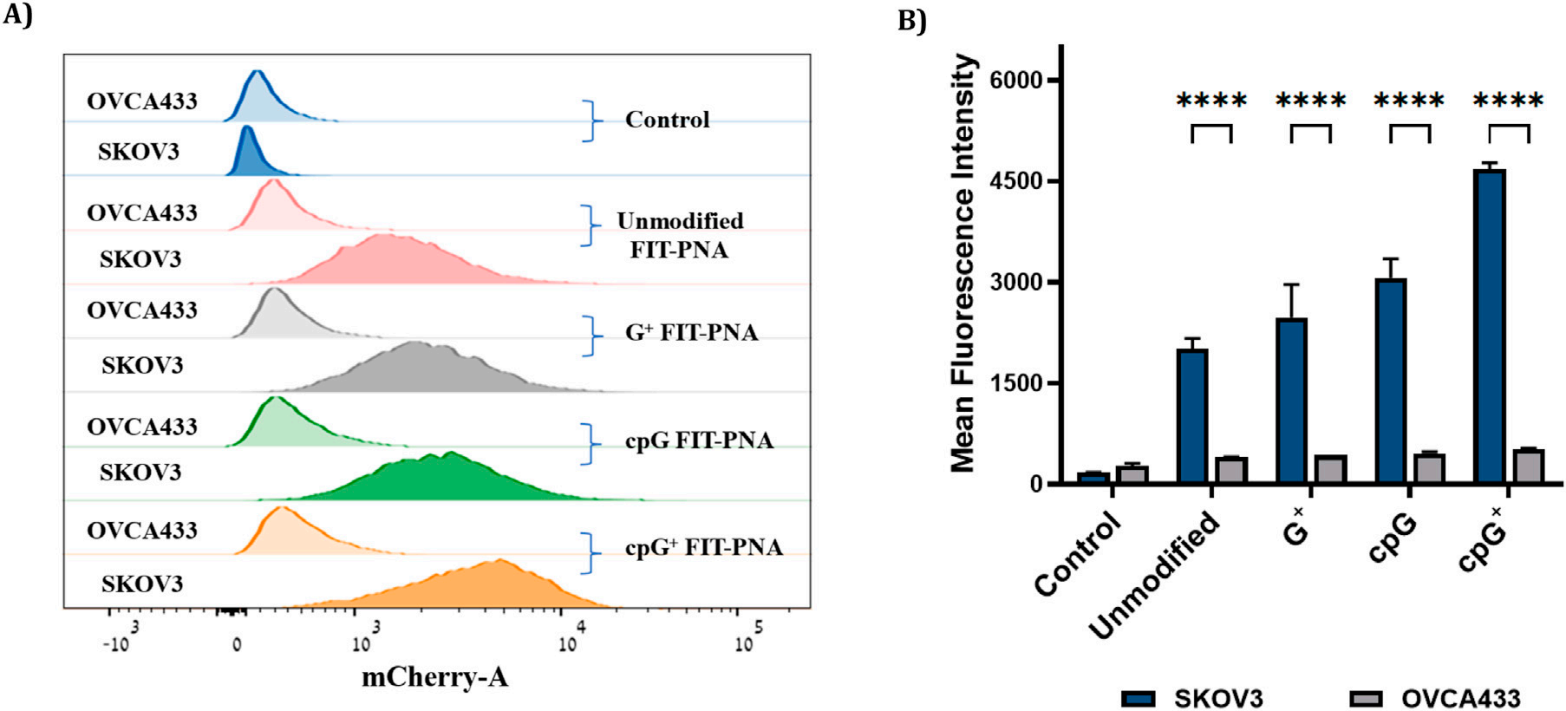
Flow cytometry analysis in OC cells (SKOV3 and OVCA433) after incubation with 2 µm of modified and unmodified CCAT1 FIT-PNAs for 3 h at 37 °C. Untreated cells from both cell lines served as control. **(A)** Histogram of FACS analysis in SKOV3 and OVCA433 cells treated with FIT-PNA. Histogram illustrates the mean fluorescence intensity plotted in horizontal axis against the number of cell events detected in the vertical axis. **(B)** Mean fluorescence intensity of FIT-PNAs in SKOV3 and OVCA433 cells. The Data is presented as the mean ± SD (n = 2). *** represents p ≤ 0.001, ** represents p ≤ 0.01 and * represents p ≤ 0.05 as determined by a Two-way ANOVA test.

To improve water solubility and cellular uptake, FIT-PNAs were conjugated to a short positively charged peptide (4 D-Lysines, (D)K_4_) at the C-terminus. We studied their ability to track lncRNA CCAT1 in two ovarian cancer cell lines: SKOV3, which expresses high levels of CCAT1 (confirmed by RT-qPCR), and OVCA433, which has minimal CCAT1 expression (Supplementary Figure S27; Supplementary Table S4). Cells were treated with 2 µM of modified and unmodified FIT-PNAs for 3 h at 37 °C, and fluorescence was analyzed via flow cytometry (Figure 6). All FIT-PNAs showed higher fluorescence in SKOV3 than in OVCA433. Notably, cpG^+^ FIT-PNA produced the strongest signal in both cell lines, with an approximately 8-fold higher fluorescence in SKOV3. It was the only modified probe that outperformed the unmodified FIT-PNA in both cell types (Supplementary Figure S28). In contrast, G^+^ and cpG FIT-PNAs showed no significant difference from the unmodified probe, indicating that these modifications offered no added benefit when adjacent to BisQ.

**FIGURE 7:**
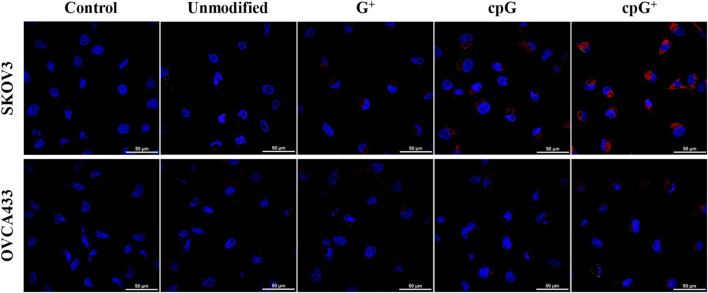
Confocal microscopy images of modified and unmodified CCAT1 FIT-PNAs (red) in SKOV3 and OVCA433 cells, overlaid with DAPI nuclear staining (blue). Scale bar = 50 µm. Cells were treated with 2 µM of FIT-PNAs for 5 h at 37 °C. Untreated cells of both cell lines served as control.

Live-cell imaging (Figure 7; Supplementary Figures S33–S34) supported these findings: SKOV3 and OVCA433 cells incubated with 2 µM FIT-PNAs for 5 h and stained with Hoechst showed higher fluorescence for cpG^+^ FIT-PNA in SKOV3. In OVCA433, only minimal fluorescence was observed for cpG^+^ FIT-PNA, and signals from other probes were undetectable. Overall, cpG^+^ FIT-PNA was the most effective for RNA detection in SKOV3 cells, with fluorescence levels correlating with CCAT1 expression, demonstrating its specificity and potential as a targeted probe for ovarian cancer cells.

## Discussion

RNA plays a crucial role in regulating cellular processes, making it a key target for diagnostic probes. Among these, oligonucleotide-based probes, particularly FIT-PNAs (forced intercalation peptide nucleic acids), stand out for their high sensitivity and specificity. In FIT-PNA design, the surrogate base (such as TO or BisQ) is typically placed centrally within the sequence, and the FIT-PNA:RNA typically forms a stable duplex despite BisQ/TO not participating in Watson-Crick-Franklin hydrogen bonding.

Previously, we developed a CCAT1 FIT-PNA to detect this oncogenic biomarker in colorectal cancer [18, 23]. However, positioning BisQ with a guanine (G) monomer adjacent (3′ side) lacked certain features to reduce background fluorescence. Our initial unmodified FIT-PNA showed only a four-fold increase in fluorescence upon duplex formation with RNA, with modest quantum yield, and negligible fluorescence in ovarian cancer cell lines. Incorporating a cyclopentane-modified PNA monomer (cpT) as a neighboring base to BisQ, improved detection of another lncRNA (FLJ22447) [22], but the enhancement for CCAT1 using cpG was still limited compared to cpT modified FLJ22447 FIT-PNA.

Introducing a combined backbone and base modification, specifically, a guanine with methylation (cpG^+^), resulted in a FIT-PNA with substantially improved performance. The cpG^+^ modification resulted in a 16-fold increase in fluorescence in duplex form and raised the quantum yield to 19%. Importantly, in live ovarian cancer cells overexpressing CCAT1, cpG^+^ FIT-PNA with a simple (D)K_4_ peptide produced a robust fluorescence signal, demonstrating cpG^+^ FIT-PNA as a sensitive probe. This simple, one-step methylation reaction on cpG offers a straightforward route to enhance FIT-PNA brightness and versatility, allowing effective targeting of challenging RNA regions without compromising structural simplicity.

In addition to improved brightness, cpG^+^ FIT-PNA exhibited the lowest limit of detection (LOD) among unmodified and other modified variants. For all FIT-PNAs, LOD values were lower when hybridized with fully complementary RNA compared to G_mm_ RNA. These findings align with the CD and T_m_ data (Figure 2; Supplementary Figure S21; Table 2) indicating greater duplex stability with the matched RNA sequence. They also demonstrate FIT-PNA’s ability to distinguish between a complementary from non-complementary RNA sequence even at low concentrations [68]. Notably, despite the lower LOD with matched RNA, fluorescence intensities were consistently higher when the FIT-PNA probes were hybridized to G_mm_ RNA across various RNA concentrations (Supplementary Figure S26). This correlates with the increased duplex fluorescence and higher UV absorbance observed for the FIT-PNAs with G_mm_ RNA (Figures 2B, 3A; Supplementary Figures S17–S20).

Although cpG^+^, cpG, and G^+^ modifications led to stronger fluorescence signals and improved detection sensitivity compared to the unmodified FIT-PNA, they did not improve mismatch discrimination (G-G mismatch RNA in particular).

In a recent study it was highlighted that introduction of a second fluorescent base surrogate into a FIT probe enabled discrimination of C to U editing in a transcript encoding the glycine receptor (GlyR) [69]. Similarly, other systems such as FRET-based probes and molecular beacons [70, 71] have also shown promise in improving mismatch discrimination while retaining sensitivity [72–74]. However, despite their high specificity, these approaches often involve complex design requirements, precise optimization of dye-dye interactions, and reduced fluorescence brightness due to spectral overlap between fluorophores. cpG^+^ offers a straightforward design with robust fluorescence performance and minimal structural complexity in comparison to other RNA sensors.

The different fluorescence profiles for CCAT1 FIT-PNAs hybridized to synthetic RNA and DNA was surprising for us. However, molecular simulations (Figures 3B,D, 4, 5) allowed us, for the first time, to gain insight into these results. Based on these simulations, the G:G mismatch RNA:FIT-PNA populates a more π-π stacked configuration for the outer quinoline ring in BisQ (Scheme 2). This π-π stacking was minimal for DNA and coincides with the lower fluorescence for G:G mismatches in FIT-PNA:DNA duplexes (Figure 5). In general, this tool may be expanded for other FIT-PNA designs to achieve, a-priori, a brighter and more specific RNA sensor.

Overall, the cpG^+^ modification offers a balanced solution - combining high brightness, ease of synthesis, and flexible design. Its simplicity and robustness make cpG^+^ FIT-PNA a promising tool for RNA detection, enabling broader application in RNA diagnostics and expanding the possibilities for sequence-specific, live-cell RNA sensing. This work represents an advance in biomedical science because it shows how one may improve the RNA sensing performance of such FIT-PNAs by tailoring their chemical structures.

## Conclusion

This study presents a significant advancement in RNA sensing: the development of a cyclopentane- and positively charged cpG^+^-modified FIT-PNA probe. The biophysical properties (BR, ϕ, LOD, and I/I_o_) and structural properties (T_m_, CD, and UV-Vis) for cpG^+^ FIT-PNA were studied with synthetic RNA and DNA. Introducing the cpG^+^ PNA monomer resulted in a substantial increase in RNA sensing that was translated to detecting the lncRNA CCAT1 in OC cancer cells (SKOV3). While challenges like mismatch discrimination remain, the significant fluorescence enhancement demonstrated its potential for highly sensitive and specific RNA diagnostics. With its simple synthesis, broad design flexibility, and imaging capabilities, the cpG^+^ FIT-PNA represents a transformative step forward in nucleic acid detection technology. We are excited to explore its application to a wider range of RNA biomarkers in future studies, paving the way for more accurate and accessible molecular diagnostics.

## Summary Table

### What Is Known About This Subject

- RNA sensing molecules have been developed for a variety of biomedical indications such as identifying RNA biomarkers related to disease.
- FIT-PNAs are a class of such RNA sensing molecules that light up (fluoresce) upon RNA hybridization.
- FIT-PNAs have been shown to detect RNA biomarkers in living cells as well as in tissues.

### What This Paper Adds

- Chemically modified FIT-PNAs are shown to improve the biophysical properties of these RNA sensors.
- Molecular modelling sheds light on the enhanced brightness of these chemically modified FIT-PNAs with complementary RNA as well as mismatched DNA and RNA sequences.
- cpG^+^ FIT-PNA detects a long non-coding RNA (CCTA1) in living ovarian cancer cells and outperforms all other FIT-PNA chemical variants.

## Data Availability

The original contributions presented in the study are included in the article/Supplementary Material, further inquiries can be directed to the corresponding author.

## References

[1] Demidov VV Potaman VN Frankkamenetskii MD Egholm M Buchard O Sonnichsen SH Stability of Peptide Nucleic-Acids in Human Serum and Cellular-Extracts. Biochem Pharm (1994) 48(6):1310–3. 10.1016/0006-2952(94)90171-6 7945427

[2] Kolevzon N Hashoul D Naik S Rubinstein A Yavin E . Single Point Mutation Detection in Living Cancer Cells by Far-Red Emitting PNA-FIT Probes. Chem Commun (2016) 52(11):2405–7. 10.1039/c5cc07502e 26735489

[3] Egholm M Buchardt O Christensen L Behrens C Freier SM Driver DA PNA Hybridizes to Complementary Oligonucleotides Obeying the Watson-Crick Hydrogen-Bonding Rules. Nature (1993) 365(6446):566–8. 10.1038/365566a0 7692304

[4] Nielsen PE Egholm M Berg RH Buchardt O . Sequence-Selective Recognition of DNA by Strand Displacement with a thymine-Substituted Polyamide. Science (1991) 254(5037):1497–500. 10.1126/science.1962210 1962210

[5] McMahon BM Mays D Lipsky J Stewart JA Fauq A Richelson E . Pharmacokinetics and Tissue Distribution of a Peptide Nucleic Acid After Intravenous Administration. Antisense Nucl Acid Drug Develop (2002) 12(2):65–70. 10.1089/108729002760070803 12074366

[6] Suparpprom C Vilaivan T . Perspectives on Conformationally Constrained Peptide Nucleic Acid (PNA): Insights into the Structural Design, Properties and Applications. RSC Chem Biol (2022) 3(6):648–97. 10.1039/d2cb00017b 35755191 PMC9175113

[7] Turner JJ Ivanova GD Verbeure B Williams D Arzumanov AA Abes S Cell-Penetrating Peptide Conjugates of Peptide Nucleic Acids (PNA) as Inhibitors of HIV-1 Tat-Dependent Trans-Activation in Cells. Nucl Acids Res (2005) 33(21):6837–49. 10.1093/nar/gki991 16321967 PMC1301599

[8] Bhingardeve P Madhanagopal BR Naick H Jain P Manoharan M Ganesh K . Receptor-Specific Delivery of Peptide Nucleic Acids Conjugated to Three Sequentially Linked N-Acetyl Galactosamine Moieties into Hepatocytes. J Org Chem (2020) 85(14):8812–24. 10.1021/acs.joc.0c00601 32529829

[9] Volpi S Cancelli U Neri M Corradini R . Multifunctional Delivery Systems for Peptide Nucleic Acids. Pharmaceuticals (2021) 14(1):14. 10.3390/ph14010014 33375595 PMC7823687

[10] Avitabile C Cerasa MT D'Aniello A Saviano M Moccia M . Recent Cutting-Edge Technologies for the Delivery of Peptide Nucleic Acid. Chem – A Eur J (2025) 31(34):e202500469. 10.1002/chem.202500469 PMC12172603 40351137

[11] Brodyagin N Katkevics M Kotikam V Ryan CA Rozners E . Chemical Approaches to Discover the Full Potential of Peptide Nucleic Acids in Biomedical Applications. Beilstein J Org Chem (2021) 17:1641–88. 10.3762/bjoc.17.116 34367346 PMC8313981

[12] Hoevelmann F Gaspar I Chamiolo J Kasper M Steffen J Ephrussi A LNA-Enhanced DNA FIT-Probes for Multicolour RNA Imaging. Chem Sci (2016) 7(1):128–35. 10.1039/c5sc03053f 29861973 PMC5950760

[13] Saarbach J Sabale PM Winssinger N . Peptide Nucleic Acid (PNA) and Its Applications in Chemical Biology, Diagnostics, and Therapeutics. Curr Opin Chem Biol (2019) 52:112–24. 10.1016/j.cbpa.2019.06.006 31541865

[14] Vilaivan T . Fluorogenic PNA Probes. Beilstein J Org Chem (2018) 14:253–81. 10.3762/bjoc.14.17 29507634 PMC5815273

[15] Sato Y Miura H Tanabe T Okeke CU Kikuchi A Nishizawa S . Fluorescence Sensing of the Panhandle Structure of the Influenza A Virus RNA Promoter by Thiazole Orange Base Surrogate-Carrying Peptide Nucleic Acid Conjugated with Small Molecule. Anal Chem (2022) 94(22):7814–22. 10.1021/acs.analchem.1c05488 35604144

[16] Tepper O Appella DH Zheng H Dzikowski R Yavin E . A Biotinylated cpFIT-PNA Platform for the Facile Detection of Drug Resistance to Artemisinin in Plasmodium falciparum. ACS Sens (2024) 9(3):1458–64. 10.1021/acssensors.3c02553 38446423 PMC10964236

[17] Chamiolo J Gaspar I Ephrussi A Seitz O . *In Vivo* Visualization and Function Probing of Transport mRNPs Using Injected FIT Probes. In: Gaspar I , editor. RNA DETECTION: Methods and Protocols. p. 273–87. 10.1007/978-1-4939-7213-5_18 29130204

[18] Kam Y Rubinstein A Naik S Djavsarov I Halle D Ariel I Detection of a Long Non-Coding RNA (CCAT1) in Living Cells and Human Adenocarcinoma of Colon Tissues Using FIT-PNA Molecular Beacons. Cancer Lett (2014) 352(1):90–6. 10.1016/j.canlet.2013.02.014 23416875

[19] Kam Y Rubinstein A Nissan A Halle D Yavin E . Detection of Endogenous K-ras mRNA in Living Cells at a Single Base Resolution by a PNA Molecular Beacon. Mol Pharm (2012) 9(3):685–93. 10.1021/mp200505k 22289057

[20] Kummer S Knoll A Socher E Bethge L Herrmann A Seitz O . Fluorescence Imaging of Influenza H1N1 mRNA in Living Infected Cells Using single-chromophore FIT-PNA. Angew Chem Int Ed (2011) 50(8):1931–4. 10.1002/anie.201005902 21328673

[21] Kummer S Knoll A Socher E Bethge L Herrmann A Seitz O . PNA FIT-Probes for the Dual Color Imaging of Two Viral mRNA Targets in Influenza H1N1 Infected Live Cells. Bioconjug Chem (2012) 23(10):2051–60. 10.1021/bc300249f 22946435

[22] Mannully ST Mahajna R Nazzal H Maree S Zheng H Appella DH Detecting the FLJ22447 lncRNA in Ovarian Cancer with Cyclopentane-Modified FIT-PNAs (cpFIT-PNAs). Biomolecules (2024) 14(6):609. 10.3390/biom14060609 38927013 PMC11202290

[23] Hashoul D Shapira R Falchenko M Tepper O Paviov V Nissan A Red-Emitting FIT-PNAs: “On Site” Detection of RNA Biomarkers in Fresh Human Cancer Tissues. Biosens and Bioelectron (2019) 137:271–8. 10.1016/j.bios.2019.04.056 31121464

[24] Hoevelmann F Gaspar I Loibl S Ermilov EA Roeder B Wengel J Brightness Through Local Constraint-LNA-Enhanced FIT Hybridization Probes for in Vivo Ribonucleotide Particle Tracking. Angew Chem Int Ed (2014) 53(42):11370–5. 10.1002/anie.201406022 25167966

[25] Oh HJ Kim J Park H Chung S Hwang DW Lee DS . Graphene-Oxide Quenching-based Molecular Beacon Imaging of Exosome-Mediated Transfer of Neurogenic miR-193a on Microfluidic Platform. Biosens Bioelectron (2019) 126:647–56. 10.1016/j.bios.2018.11.027 30529896

[26] Ryoo SR Lee J Yeo J Na HK Kim YK Jang H Quantitative and Multiplexed microRNA Sensing in Living Cells Based on Peptide Nucleic Acid and Nano Graphene Oxide (PANGO). ACS Nano (2013) 7(7):5882–91. 10.1021/nn401183s 23767402

[27] Sabale PM George JT Srivatsan SG . A Base-Modified PNA–Graphene Oxide Platform as a Turn-On Fluorescence Sensor for the Detection of Human Telomeric Repeats. Nanoscale (2014) 6(18):10460–9. 10.1039/C4NR00878B 24981293

[28] Zhu Y Ji Q Hong M . Construction of Graphene Oxide Probes Loaded with Antisense Peptide Nucleic Acid and Doxorubicin for Regulating Telomerase Activity and Inducing Apoptosis of Cancer Cells. Biosensors (2025) 15(6):337. 10.3390/bios15060337 40558419 PMC12190608

[29] Abdel-Hamied M Guo M Wei Y Bansmann J El Nashar RM Oswald F A Novel Hybrid Biosensor for miRNA Detection Based on Peptide Nucleic Acids and Molecularly Imprinted Polymers. Bioelectrochem. (2025) 165:108964. 10.1016/j.bioelechem.2025.108964 40048946

[30] Fathi N Saadati A Hasanzadeh M Samiei M . Chemical Binding of Pyrrolidinyl Peptide Nucleic Acid (acpcPNA-T9) Probe with AuNPs Toward Label-Free Monitoring of miRNA-21: A Novel Biosensing Platform for Biomedical Analysis and POC Diagnostics. J *J Mol Recog* (2021) 34(8):e2893. 10.1002/jmr.2893 33822429

[31] Kangkamano T Numnuam A Limbut W Kanatharana P Vilaivan T Thavarungkul P . Pyrrolidinyl PNA polypyrrole/silver Nanofoam Electrode as a Novel Label-Free Electrochemical miRNA-21 Biosensor. Biosens Bioelectron (2018) 102:217–25. 10.1016/j.bios.2017.11.024 29149687

[32] Xu S Wang G Feng Y Zheng J Huang L Liu J PNA-Functionalized, Silica Nanowires-Filled Glass Microtube for Ultrasensitive and Label-Free Detection of miRNA-21. Anal Chem (2024) 96(19):7470–8. 10.1021/acs.analchem.3c05839 38696229

[33] Yu X Ding S Zhao Y Xu M Wu Z Zhao C . A Highly Sensitive and Robust Electrochemical Biosensor for microRNA Detection Based on PNA-DNA Hetero-Three-Way Junction Formation and Target-Recycling Catalytic Hairpin Assembly Amplification. Talanta (2024) 266(Pt 1):125020. 10.1016/j.talanta.2023.125020 37541007

[34] Amouzadeh TM . An Electrochemical PNA-Based Sensor for the Detection of the SARS-CoV-2 RdRP by Using Surface-Initiated-Reversible-Addition-Fragmentation-Chain-Transfer Polymerization Technique. Talanta (2023) 259:124490. 10.1016/j.talanta.2023.124490 37004398 PMC10060013

[35] Liu L Lu H Shi R Peng XX Xiang Q Wang B Synergy of Peptide-Nucleic Acid and Spherical Nucleic Acid Enabled Quantitative and Specific Detection of Tumor Exosomal MicroRNA. Anal Chem (2019) 91(20):13198–205. 10.1021/acs.analchem.9b03622 31553171

[36] Moccia M Caratelli V Cinti S Pede B Avitabile C Saviano M Paper-Based Electrochemical Peptide Nucleic Acid (PNA) Biosensor for Detection of miRNA-492: A Pancreatic Ductal Adenocarcinoma Biomarker. Biosens Bioelectron (2020) 165:112371. 10.1016/j.bios.2020.112371 32729503

[37] Chen L Ding S Yuan X Zhao Y Zhao C . PNA-Based Blocker Displacement Amplification System for *in situ* Visualization of Individual microRNAs in Cancer Cells. Microchem J (2025) 214:113927. 10.1016/j.microc.2025.113927

[38] Yuan H Zhou B-m Gao W Wang L-j Zhang C-y . Construction of an Endogenous ATP-Driven self-dissociated DNA Nanoflower for Rapid Imaging of Multiple Long Noncoding RNAs and Chemotherapy. Chem Eng J (2025) 518:164594. 10.1016/j.cej.2025.164594

[39] Xu M Fu P Xing S Zhao Y Zhao C . A PNA-DNA(2) Triple-Helix Molecular Switch-Based Colorimetric Sensor for Sensitive and Specific Detection of microRNAs from Cancer Cells. Chembiochem (2020) 21(18):2667–75. 10.1002/cbic.202000155 32304168

[40] Li X Song J Chen B-L Wang B Li R Jiang H-M A Label-Free Colorimetric Assay for Detection of c-Myc mRNA Based on Peptide Nucleic Acid and Silver Nanoparticles. Sci Bull (2016) 61(4):276–81. 10.1007/s11434-016-1004-3

[41] Koehler O Jarikote DV Seitz O . Forced Intercalation Probes (FIT Probes): Thiazole Orange as a Fluorescent Base in Peptide Nucleic Acids for Homogeneous Single-Nucleotide-Polymorphism Detection. ChemBioChem (2005) 6(1):69–77. 10.1002/cbic.200400260 15584015

[42] Haralampiev I Schade M Chamiolo J Jolmes F Prisner S Witkowski PT A Fluorescent RNA Forced-Intercalation Probe as a Pan-Selective Marker for Influenza A Virus Infection. ChemBioChem (2017) 18(16):1589–92. 10.1002/cbic.201700271 28557173

[43] Hoevelmann F Gaspar I Ephrussi A Seitz O . Brightness Enhanced DNA FIT-Probes for Wash-Free RNA Imaging in Tissue. J Am Chem Soc (2013) 135(50):19025–32. 10.1021/ja410674h 24295172

[44] Pokorski JK Nam J-M Vega RA Mirkin CA Appella DH . Cyclopentane-Modified PNA Improves the Sensitivity of Nanoparticle-Based Scanometric DNA Detection. Chem Commun (2005)(16) 2101–3. 10.1039/B418383E 15846413

[45] Pokorski JK Witschi MA Purnell BL Appella DH . (S,S)-Trans-Cyclopentane-Constrained Peptide Nucleic Acids. A General Backbone Modification that Improves Binding Affinity and Sequence Specificity. J Am Chem Soc (2004) 126(46):15067–73. 10.1021/ja046280q 15548003

[46] Zheng HC Botos I Clausse V Nikolayevskiy H Rastede EE Fouz MF Conformational Constraints of Cyclopentane Peptide Nucleic Acids Facilitate Tunable Binding to DNA. Nucl Acids Res (2021) 49(2):713–25. 10.1093/nar/gkaa1249 33406227 PMC7826248

[47] Tepper O Zheng HC Appella DH Yavin E . Cyclopentane FIT-PNAs: Bright RNA Sensors. Chem Commun (2021) 57(4):540–3. 10.1039/d0cc07400d 33336664

[48] Gharib E Nazemalhosseini-Mojarad E Baghdar K Nayeri Z Sadeghi H Rezasoltani S Identification of a Stool Long Non-Coding RNAs Panel as a Potential Biomarker for Early Detection of Colorectal Cancer. J Clin Lab Anal (2021) 35(2):e23601. 10.1002/jcla.23601 33094859 PMC7891513

[49] Zhao W Song M Zhang J Kuerban M Wang H . Combined Identification of Long Non-Coding RNA CCAT1 and HOTAIR in Serum as an Effective Screening for Colorectal Carcinoma. Int J Clin Exp Pathol (2015) 8(11):14131–40. 26823726 PMC4713512

[50] Sánchez-Salcedo R Miranda-Castro R de-los-Santos-Álvarez N Fernández-Martínez D García-Flórez LJ Lobo-Castañón MJ . An Electrochemical Genosensing Platform for the Relative Quantification of the Circulating Long Noncoding RNA CCAT1 to Aid in the Diagnosis of Colorectal Cancer. Sens Act B: Chem (2023) 376:132940. 10.1016/j.snb.2022.132940

[51] Hibino M Aiba Y Shoji O . Cationic Guanine: Positively Charged Nucleobase with Improved DNA Affinity Inhibits Self-Duplex Formation. Chem Commun (2020) 56(17):2546–9. 10.1039/d0cc00169d 32040115

[52] Ghafouri-Fard S Taheri M . Colon Cancer-Associated Transcripts 1 and 2: Roles and Functions in Human Cancers. J Cell Physiol (2019) 234(9):14581–600. 10.1002/jcp.28176 30693526

[53] Mu Y Li N Cui YL . The lncRNA CCAT1 Upregulates TGFβR1 via Sponging miR-490-3p to Promote TGFβ1-Induced EMT of Ovarian Cancer Cells. Cancer Cell Int (2018) 18:145. 10.1186/s12935-018-0604-1 30250403 PMC6148998

[54] Nissan A Stojadinovic A Mitrani-Rosenbaum S Halle D Grinbaum R Roistacher M Colon Cancer Associated Transcript-1: A Novel RNA Expressed in Malignant and Pre-Malignant Human Tissues. Int J Cancer (2012) 130(7):1598–606. 10.1002/ijc.26170 21547902

[55] Xin Y Li Z Shen JX Chan MTV Wu WKK . CCAT1: A Pivotal Oncogenic Long Non-Coding RNA in Human Cancers. Cell Prolif (2016) 49(3):255–60. 10.1111/cpr.12252 27134049 PMC6496795

[56] Brovkina OI Pronina IV Burdennyy AM Uroshlev LA Filippova EA Fridman MV The Role of Long Non-Coding RNA CCAT1 and SNHG14 in Activation of Some Protein-Coding Genes Associated with the Development of Ovarian Cancer. Bull Exp Biol Med (2022) 172(6):760–4. 10.1007/s10517-022-05473-8 35501644

[57] Cao Y Shi HR Ren F Jia YY Zhang RT . Long Non-Coding RNA CCAT1 Promotes Metastasis and Poor Prognosis in Epithelial Ovarian Cancer. Exp Cell Res (2017) 359(1):185–94. 10.1016/j.yexcr.2017.07.030 28754469

[58] Coni P Madeddu A Kuqi L Pichiri G Occhinegro A Ratto D LncRNA Colon Cancer-Associ Ate Transcript 1 (CCAT1) in Ovarian Cancer. Eur Rev Med Pharm Sci (2018) 22(6):1525–7. 10.26355/eurrev_201803_14554 29630091

[59] Lai XJ Cheng HF . LncRNA Colon Cancer-Associated Transcript 1 (CCAT1) Promotes Proliferation and Metastasis of Ovarian Cancer via miR-1290. Eur Rev Med Pharm Sci (2018) 22(2):322–8. 10.26355/eurrev_201801_14175 29424889

[60] Zheng H Saha M Appella DH . Synthesis of Fmoc-Protected (S,S)-Trans-Cyclopentane Diamine Monomers Enables the Preparation and Study of Conformationally Restricted Peptide Nucleic Acids. Org Lett (2018) 20(23):7637–40. 10.1021/acs.orglett.8b03374 30460846

[61] Nazzal H Gupta MK Fadila A Yavin E . A Facile Synthesis of Red-Shifted Bis-Quinoline (BisQ) Surrogate Base. Molecules (2024) 29(17):4136. 10.3390/molecules29174136 39274984 PMC11397033

[62] Brouwer AM . Standards for Photoluminescence Quantum Yield Measurements in Solution (IUPAC Technical Report). Pure Appl Chem (2011) 83(12):2213–28. 10.1351/pac-rep-10-09-31

[63] Fery-Forgues S Lavabre D . Are Fluorescence Quantum Yields so Tricky to Measure? A Demonstration Using Familiar Stationery Products. J Chem (1999) 76(9):1260. 10.1021/ed076p1260

[64] Würth C Grabolle M Pauli J Spieles M Resch-Genger U . Relative and Absolute Determination of Fluorescence Quantum Yields of Transparent Samples. Nat Protoc (2013) 8(8):1535–50. 10.1038/nprot.2013.087 23868072

[65] Armbruster DA Pry T . Limit of Blank, Limit of Detection and Limit of Quantitation. Clin Biochem Rev (2008) 29(Suppl. 1):S49–52. 18852857 PMC2556583

[66] Kumar P Jain DR . Cγ-Aminopropylene Peptide Nucleic Acid (amp-PNA): Chiral Cationic PNAs with Superior PNA:DNA/RNA Duplex Stability and Cellular Uptake. Tetrahedron (2015) 71(21):3378–84. 10.1016/j.tet.2015.03.093

[67] Piacenti V Langella E Autiero I Nolan JC Piskareva O Adamo MFA A Combined Experimental and Computational Study on Peptide Nucleic Acid (PNA) Analogues of Tumor Suppressive miRNA-34a. Bioorg Chem (2019) 91:103165. 10.1016/j.bioorg.2019.103165 31419642

[68] Saady A Wojtyniak M Varon E Böttner V Kinor N Shav-Tal Y Specific, Sensitive, and Quantitative Detection of HER-2 mRNA Breast Cancer Marker by Fluorescent Light-Up Hybridization Probes. Bioconjug Chem. (2020) 31(4):1188–98. 10.1021/acs.bioconjchem.0c00130 32208683

[69] Schöllkopf S Knoll A Homer A Seitz O . Double FIT Hybridization Probes - Towards Enhancing Brightness, Turn-on and Specificity of RNA Detection. Chem Sci (2023) 14(15):4166–73. 10.1039/d3sc00363a 37063796 PMC10094420

[70] Mergny JL Boutorine AS Garestier T Belloc F Rougée M Bulychev NV Fluorescence Energy Transfer as a Probe for Nucleic Acid Structures and Sequences. Nucl Acids Res (1994) 22(6):920–8. 10.1093/nar/22.6.920 8152922 PMC307910

[71] Tsourkas A Behlke MA Rose SD Bao G . Hybridization Kinetics and Thermodynamics of Molecular Beacons. Nucl Acids Res (2003) 31(4):1319–30. 10.1093/nar/gkg212 12582252 PMC150230

[72] Fang G-m Chamiolo J Kankowski S Hövelmann F Friedrich D Löwer A A Bright FIT-PNA Hybridization Probe for the Hybridization State Specific Analysis of a C → U RNA Edit via FRET in a Binary System. Chem Sci (2018) 9(21):4794–800. 10.1039/C8SC00457A 29910930 PMC5982193

[73] Holzhauser C Wagenknecht HA . In-Stem-Labeled Molecular Beacons for Distinct Fluorescent Color Readout. Angew Chem Int Ed (2011) 50(32):7268–72. 10.1002/anie.201101968 21717540

[74] Homer A Knoll A Gruber U Seitz O . Light Harvesting FIT DNA Hybridization Probes for brightness-enhanced RNA Detection. Chem Sci (2025) 16(2):846–53. 10.1039/d4sc06729k 39650216 PMC11622247

