# Supplementary material for: 7-Methylguanine With a Cyclopentane Backbone: A Bright Combination for a FIT-PNA RNA Sensor
Source: Br J Biomed Sci. 2025 Nov 21;82:15526. doi: 10.3389/bjbs.2025.15526 (PMC12678193; doi:10.3389/bjbs.2025.15526)
Supplement: Supplementary file 1 [file DataSheet1.pdf]

Supporting Information

7-Methylguanine with a cyclopentane backbone: A bright combination for a FIT-PNA RNA sensor

Salam Maree[a], Pinaki Chanda[a], Sheethal Thomas Mannully[a], Hongchao Zheng[b], Daniel H. Appella[b], and Eylon Yavin*[a]

[a]The Institute for Drug Research, The School of Pharmacy, The Faculty of Medicine, The Hebrew University of Jerusalem, Hadassah Ein-Kerem, Jerusalem 9112102, Israel. E-mail: eylony@ekmd.huji.ac.il

[b]Synthetic Bioactive Molecules Section, Laboratory of Bioorganic Chemistry (LBC), National Institute of Diabetes and Digestive and Kidney Diseases (NIDDK), National Institutes of Health, 8 Center Drive, Room 404, Bethesda, Maryland 20892, United States.

Supporting Information

Tables of contents:

**Sequences used for studies**	**Table S1**
**HPLC and MS analysis of CCAT1 FIT-PNAs**	**Figures S1-S7**
**T_m measurements**	**Figures S8-S15, Table S2-S3**
**Mismatch sensitivity of modified and unmodified CCAT1 FIT-PNAs**	**Figures S16-S17**
**UV-Vis spectra of FIT-PNAs**	**Figure S18-S20**
**Circular Dichroism (CD)**	**Figure S21**
**Quantum yields determination of modified and unmodified CCAT1 FIT-PNAs**	**Figures S22-S25**
**Limit of Detection (LOD)**	**Figure S26**
**RT-qPCR and primer sequences**	**Figure S27, Table S4**
**Flow Cytometry Analysis**	**Figures S28-S32**
**Confocal Microscopy**	**Figures S33-S34**
**Characterization of cpG$^+$ PNA monomer (^{1}H, ^{13}C NMR spectra and HRMS)**	**Figures S35-S36**
**Molecular Simulations of CCAT1 FIT-PNA with complementary and GG mismatch RNA/DNA**	**Figures S37-S41, Tables S5-S6**
**References**	

Table S1. Sequences used for studies.

Entry	Description	PNA/RNA/DNA Sequence	Mass	
			Calculated	Observed
Unmodified PNA	Unmodified FIT-PNA (control)	$^{3'}$(4)K_D-GTGAATG-**BisQ**TCCAACC$^{-5'}$	4734.08	4740.11
G^+ PNA	G^+ modified FIT-PNA	$^{3'}$(4)K_D-GTGAAT**G**$^+$-**BisQ**-TCCAACC$^{-5'}$	4749.92	4749.974
cpG PNA	cpG modified FIT-PNA	$^{3'}$(4)K_D-GTGAAT**cpG**-**BisQ**-TCCAACC$^{-5'}$	4774.11	4780.154
cpG^+ PNA	cpG^+ modified FIT-PNA	$^{3'}$(4)K_D-GTGAAT**cpG**$^+$-**BisQ**-TCCAACC$^{-5'}$	4789.14	4790.130
CCAT1-A PNA	Unmodified FIT-PNA with A substituted for G	$^{3'}$(4)K_D-GTGAAT**A**-**BisQ**-TCCAACC$^{-5'}$	4718.08	4719.974
CCAT1-T PNA	Unmodified FIT-PNA with T substituted for G	$^{3'}$(4)K_D-GTGAAT**T**-**BisQ**-TCCAACC$^{-5'}$	4709.08	4709.676
CCAT1-C PNA	Unmodified FIT-PNA with C substituted for G	$^{3'}$(4)K_D-GTGAAT**C**-**BisQ**-TCCAACC$^{-5'}$	4694.08	4699.534
G-C (RNA)	Fully matched RNA	$^{5'}$C-A-C-U-U-A-C-C-A-G-G-U-U-G-G$^{-3'}$	-	-
G-A (RNA)	Mismatched RNA	$^{5'}$C-A-C-U-U-A-**A**-C-A-G-G-U-U-G-G$^{-3'}$	-	-
G-G (RNA)	Mismatched RNA	$^{5'}$C-A-C-U-U-A-**G**-C-A-G-G-U-U-G-G$^{-3'}$	-	-
G-U (RNA)	Mismatched RNA	$^{5'}$C-A-C-U-U-A-**U**-C-A-G-G-U-U-G-G$^{-3'}$	-	-
G-C (DNA)	Fully matched DNA	$^{5'}$C-A-C-T-T-A-C-C-A-G-G-T-T-G-G$^{-3'}$	-	-
G-A (DNA)	Mismatched DNA	$^{5'}$C-A-C-T-T-A-**A**-C-A-G-G-T-T-G-G$^{-3'}$	-	-
G-G (DNA)	Mismatched DNA	$^{5'}$C-A-C-T-T-A-**G**-C-A-G-G-T-T-G-G$^{-3'}$	-	-
G-T (DNA)	Mismatched DNA	$^{5'}$C-A-C-T-T-A-**T**-C-A-G-G-T-T-G-G$^{-3'}$	-	-

(4)K_D = 4 D-lysines, BisQ is marked in blue, guanine modified PNA bases are marked in red and bold letters represent substitutions in the PNA sequence/mismatches in RNA sequence mismatches in the RNA/DNA sequence.

HPLC and Maldi-TOF MS of modified and unmodified CCAT1 FIT-PNAs

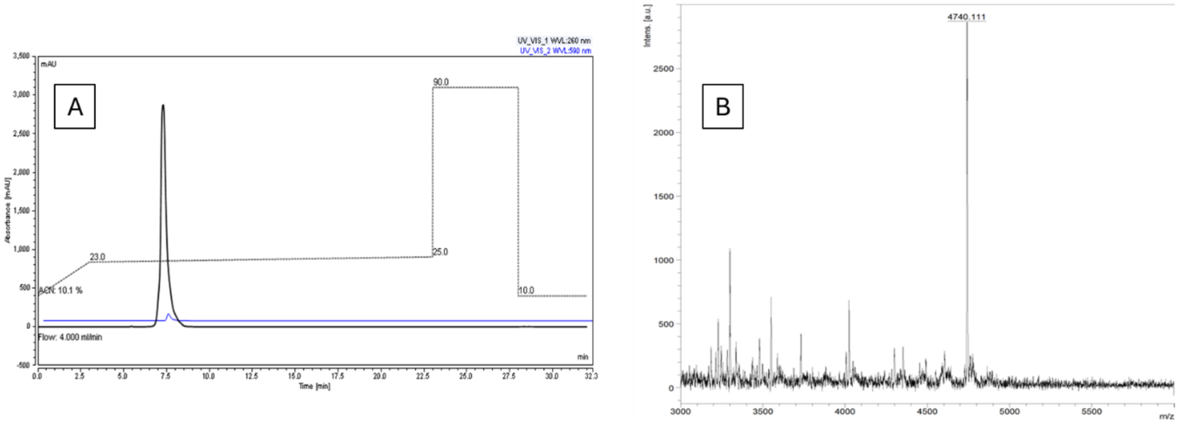

Figure S1: **Characterization of unmodified CCAT1 FIT-PNA. (A)** HPLC chromatogram. Eluents: A (0.1% TFA in water) and B (MeCN) were used in a linear gradient (10-23 % B over 3 min, 23-25% over 20 min) with a flow rate of 4 mL/min and elution time at 7.5 min. **(B)** Maldi-TOF MS. M_{calc} = 4734.08, M_{obs} =4740.11.

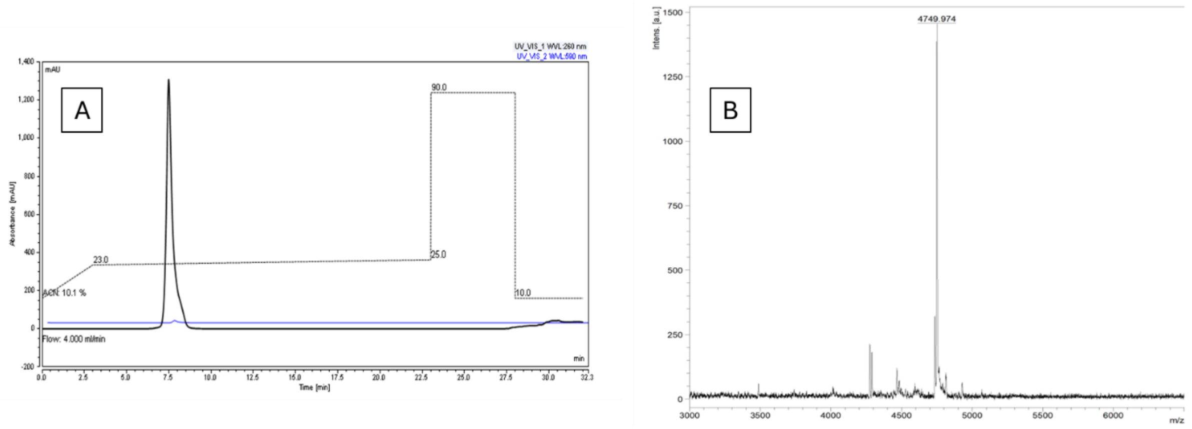

Figure S2: Characterization of G^{+} modified CCAT1 FIT-PNA. **(A)** HPLC chromatogram. Eluents: A (0.1% TFA in water) and B (MeCN) were used in a linear gradient (10-23 % B over 3 min, 23-25% over 20 min) with a flow rate of 4 mL/min and elution time at 7.5 min. **(B)** Maldi-TOF MS. M_{calc} = 4749.92, M_{obs} =4749.974.

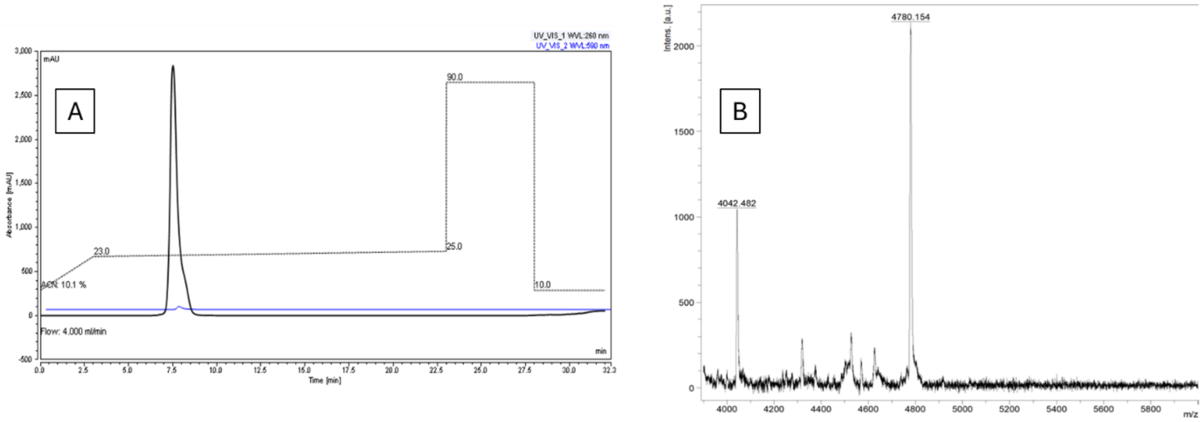

Figure S3: Characterization of cpG modified CCAT1 FIT-PNA. **(A)** HPLC chromatogram. Eluents: A (0.1% TFA in water) and B (MeCN) were used in a linear gradient (10-23 % B over 3 min, 23-25% over 20 min) with a flow rate of 4 mL/min and elution time at 7.5 min. **(B)** Maldi-TOF MS. M_{calc} = 4774.11, M_{obs} =4780.154.

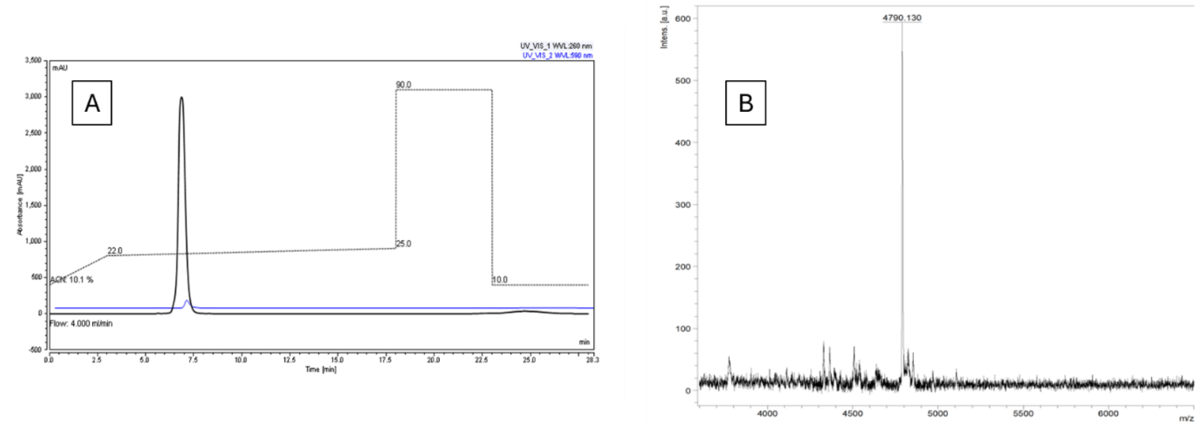

Figure S4: Characterization of cpG^{+} modified CCAT1 FIT-PNA. **(A)** HPLC chromatogram. Eluents: A (0.1% TFA in water) and B (MeCN) were used in a linear gradient (10-22 % B over 3 min, 22-25% over 20 min) with a flow rate of 4 mL/min and elution time at 7 min. **(B)** Maldi-TOF MS. M_{calc} = 4789.14, M_{obs} =4790.130.

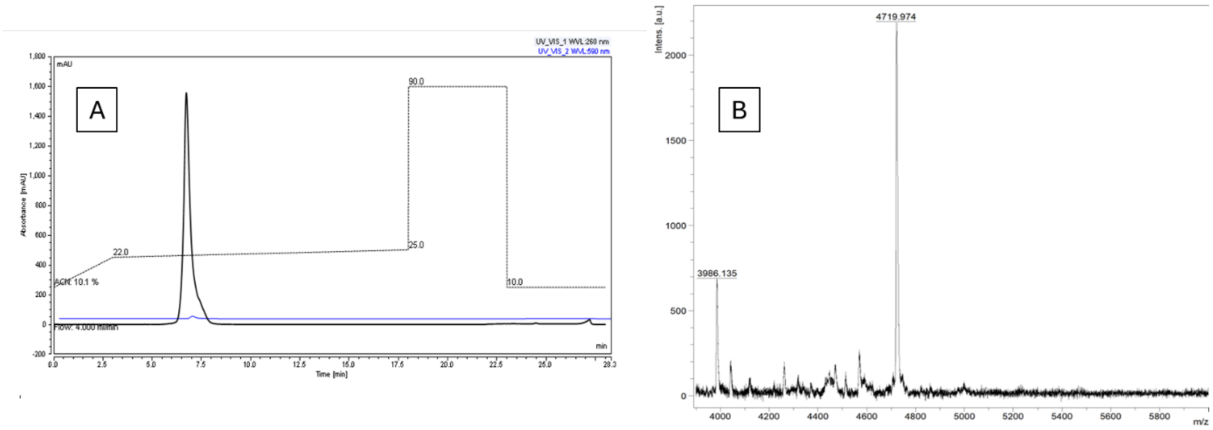

Figure S5: Characterization of CCAT1-A FIT-PNA. **(A)** HPLC chromatogram. Eluents: A (0.1% TFA in water) and B (MeCN) were used in a linear gradient (10-22 % B over 3 min, 22-25% over 15 min) with a flow rate of 4 mL/min and elution time at 7 min. **(B)** Maldi-TOF MS. M_{calc} = 4718.08, M_{obs} =4719.974.

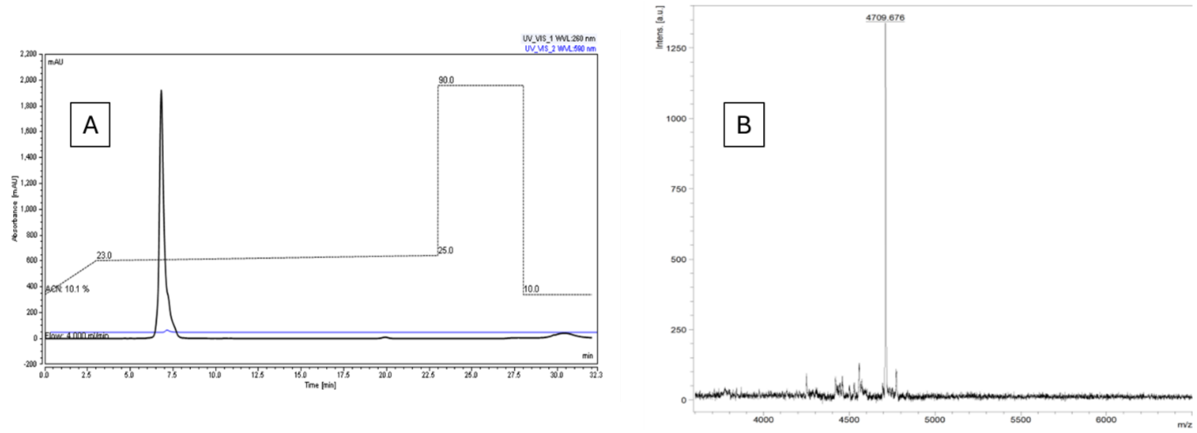

Figure S6: Characterization of CCAT1-T FIT-PNA. **(A)** HPLC chromatogram. Eluents: A (0.1% TFA in water) and B (MeCN) were used in a linear gradient (10-23 % B over 3 min, 23-25% over 20 min) with a flow rate of 4 mL/min and elution time at 7 min. **(B)** Maldi-TOF MS. M_{calc} = 4709.08, M_{obs} =4709.676.

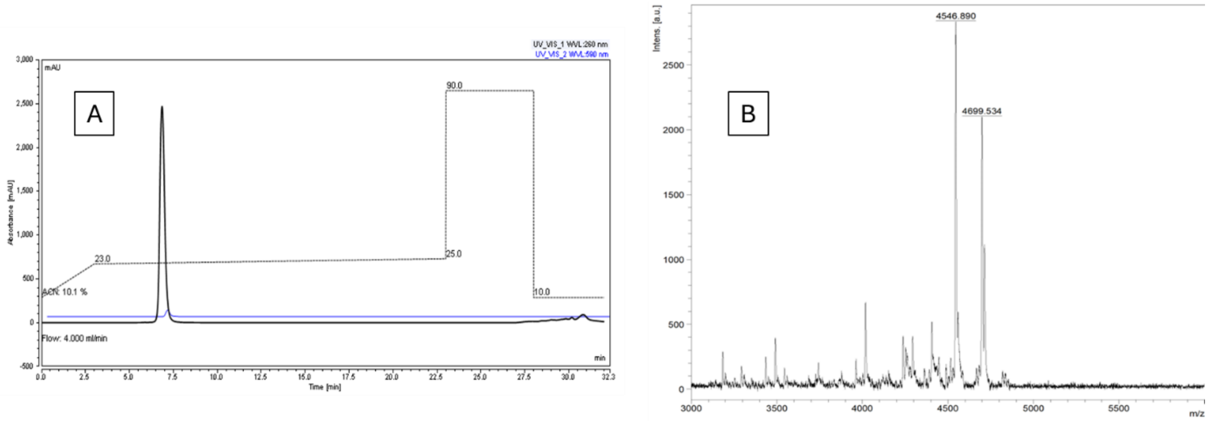

Figure S7: Characterization of CCAT1-C FIT-PNA. (A) HPLC chromatogram. Eluents: A (0.1% TFA in water) and (B (MeCN) were used in a linear gradient (10-23 % B over 3 min, 23-25% over 20 min) with a flow rate of 4 mL/min and elution time at 7 min. **(B)** Maldi-TOF MS. M_{calc} = 4694.08, M_{obs} =4699.534.

T_m measurements

Table S2. T_m measurements of modified and unmodified CCAT1 FIT-PNAs with complementary and mismatched synthetic RNA.

Entry	T_m (^{0}C) of PNA: RNA/ mmRNA duplex			
	FM (G-C)	G-A	G-G	G-U
Unmodified PNA	**65.55** (±0.2)	60 (±0.12)	58.97 (±0.1)	59.87 (±0.11)
G^+ PNA	**62.59** (±0.13)	59.3 (±0.1)	58.2 (±0.1)	57.7 (±0.08)
cpG PNA	**68.8** (±0.23)	62.18 (±0.1)	60.88 (±0.1)	61.6 (±0.11)
cpG^+ PNA	**61.96** (±0.08)	58.1 (±0.11)	56.86 (±0.15)	56.7 (±0.09)

Table S3. T_m measurements of modified and unmodified CCAT1 FIT-PNAs with complementary and mismatched synthetic DNA.

Entry	T_m (^{0}C) of PNA: DNA/ mmDNA duplex			
	FM (G-C)	G-A	G-G	G-T
Unmodified PNA	**63** (±0.1)	57.2 (±0.09)	56.5 (±0.05)	57.5 (±0.06)
G^+ PNA	**60.09** (±0.09)	56.57 (±0.12)	57.3 (±0.08)	57.7 (±0.07)
cpG PNA	**63.58** (±0.09)	59.57 (±0.07)	54.17 (±0.06)	58.1 (±0.06)
cpG^+ PNA	**60.3** (±0.08)	59.3 (±0.1)	57.45 (±0.09)	56.9 (±0.09)

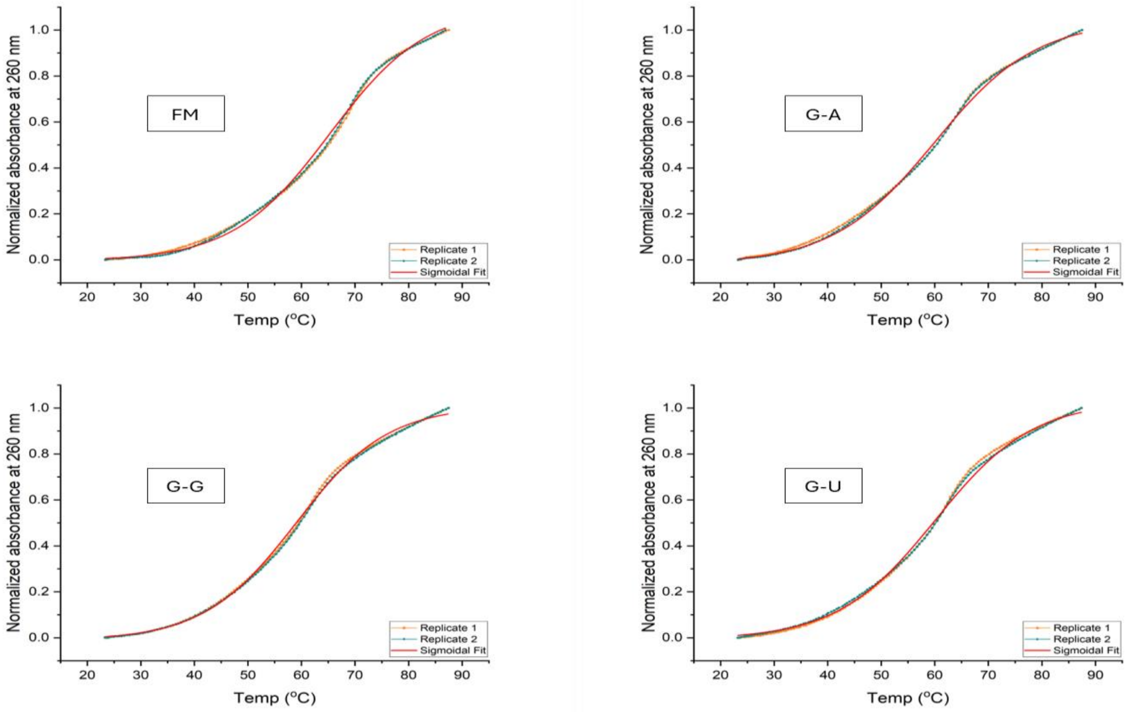

Figure S8: Melting curve profiles for unmodified CCAT1 FIT-PNA annealed to complementary and mismatched synthetic RNA. [FIT-PNA] = [RNA] = 2μM.

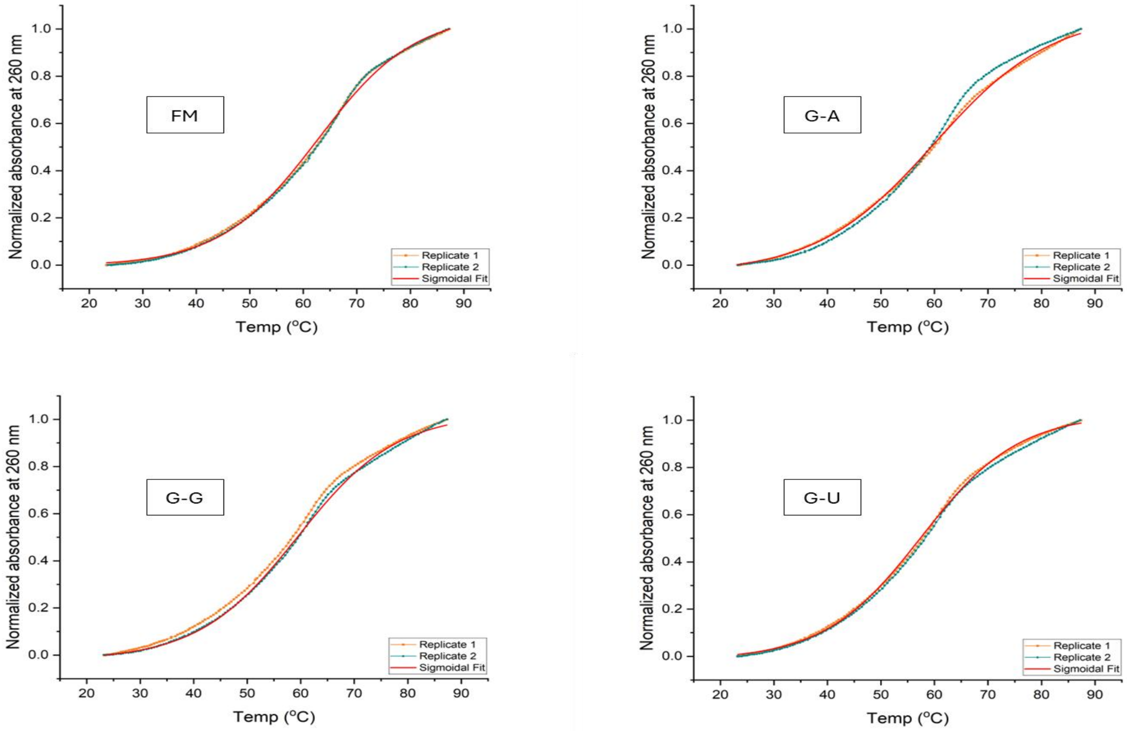

Figure S9: Melting curve profiles for G^{+} modified CCAT1 FIT-PNA annealed to complementary and mismatched synthetic RNA. [FIT-PNA] = [RNA] = 2μM.

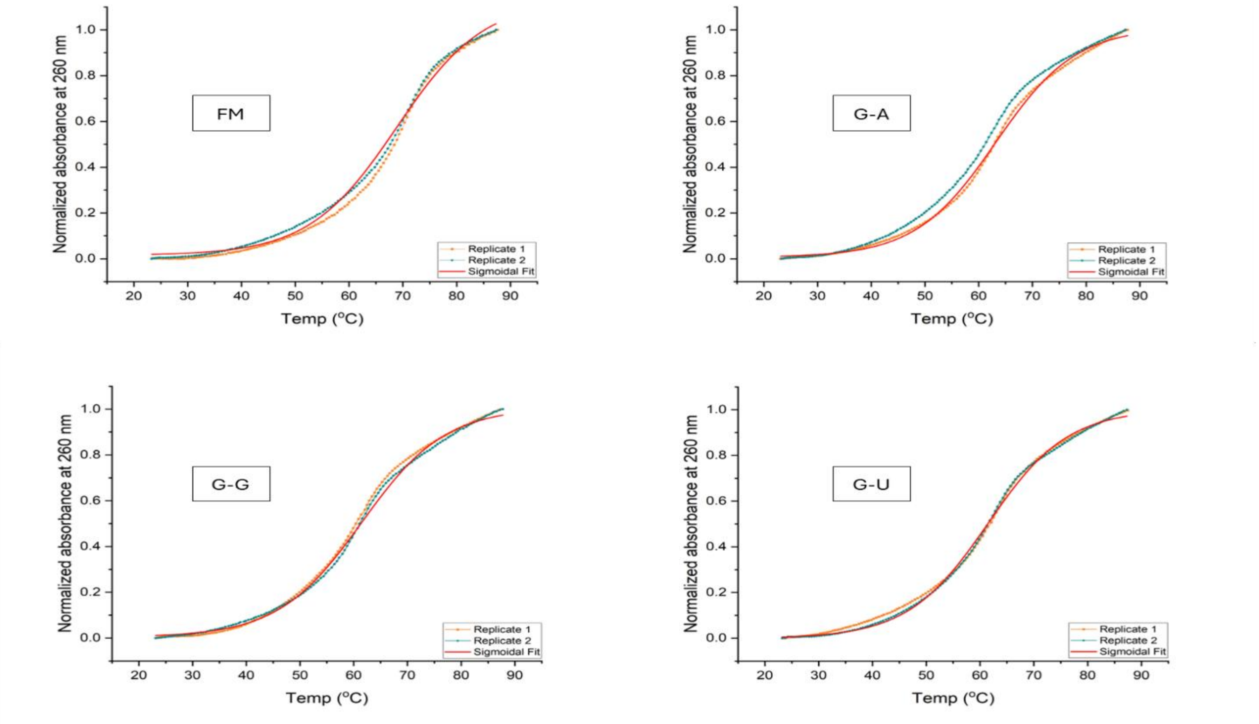

Figure S10: Melting curve profiles for cpG modified CCAT1 FIT-PNA annealed to complementary and mismatched synthetic RNA. [FIT-PNA] = [RNA] = 2μM.

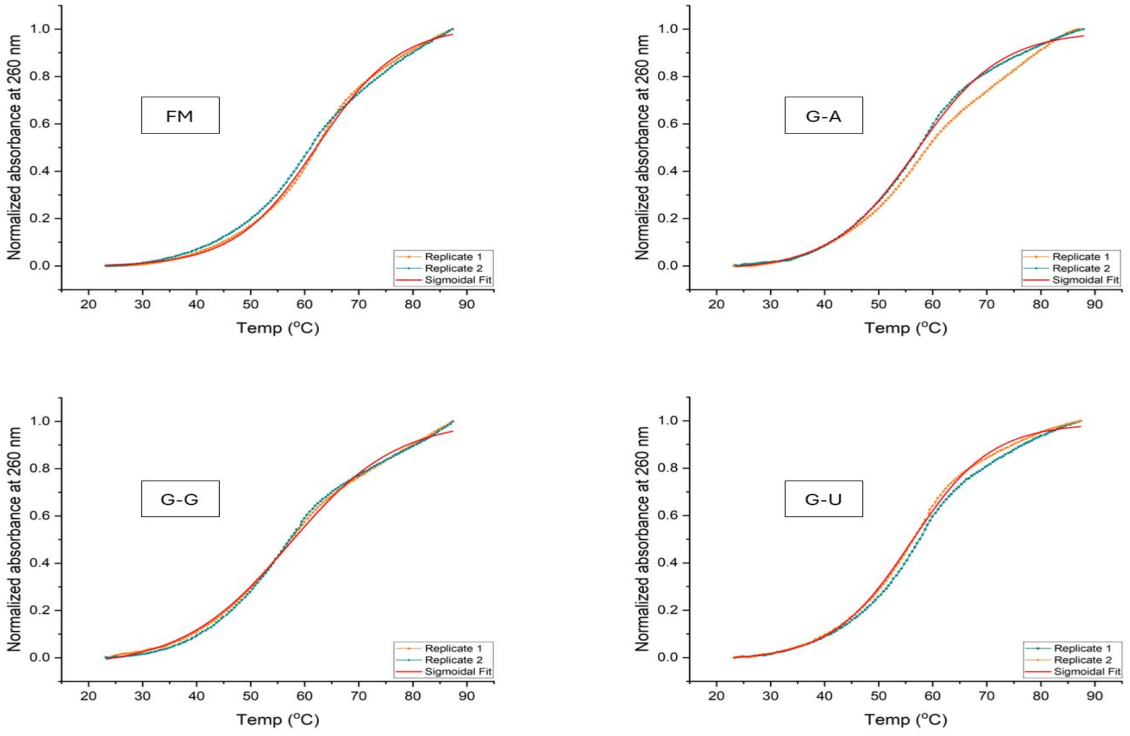

Figure S11: Melting curve profiles for cpG^{+} modified CCAT1 FIT-PNA annealed to complementary and mismatched synthetic RNA. [FIT-PNA] = [RNA] = 2μM.

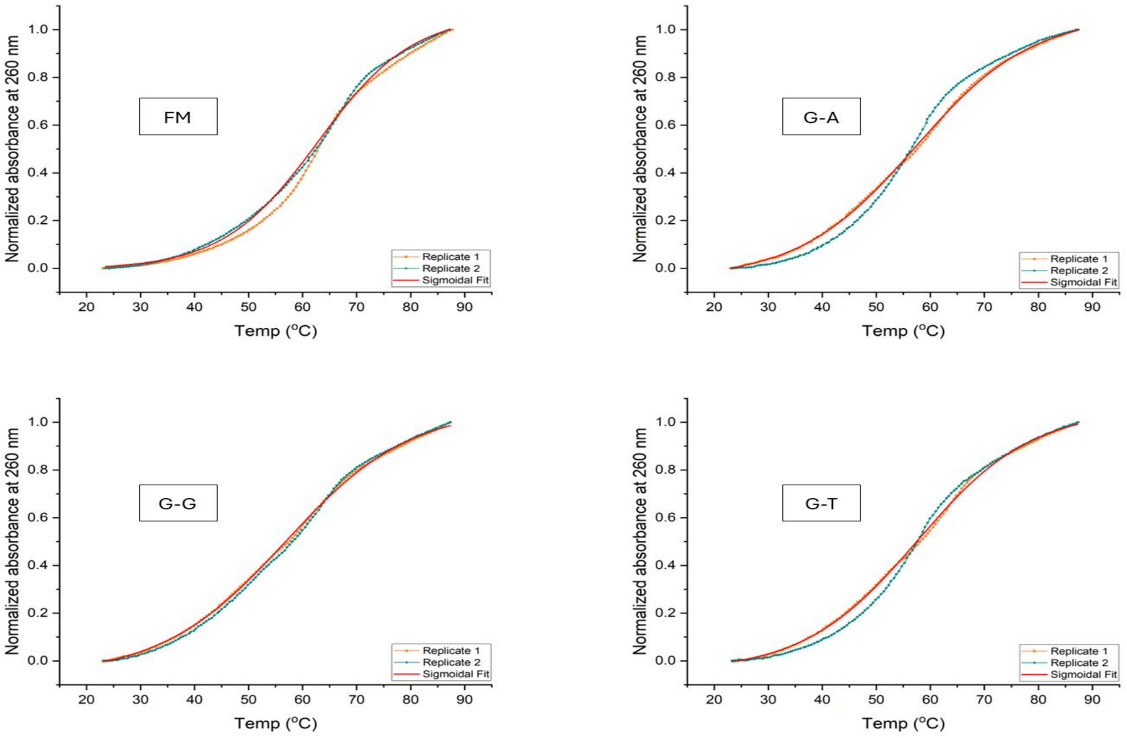

Figure S12: Melting curve profiles for unmodified CCAT1 FIT-PNA annealed to complementary and mismatched synthetic DNA. [FIT-PNA] =[DNA] = 2μM.

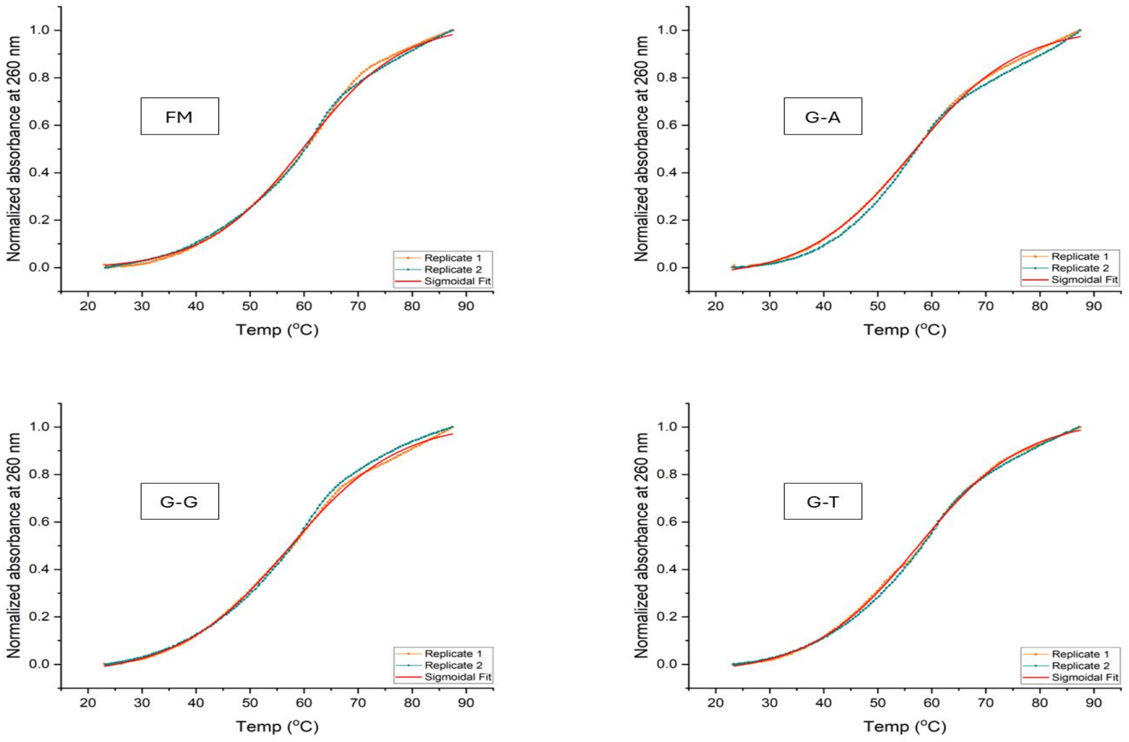

Figure S13: Melting curve profiles for G^{+} modified CCAT1 FIT-PNA annealed to complementary and mismatched synthetic DNA. [FIT-PNA] =[DNA] = 2μM.

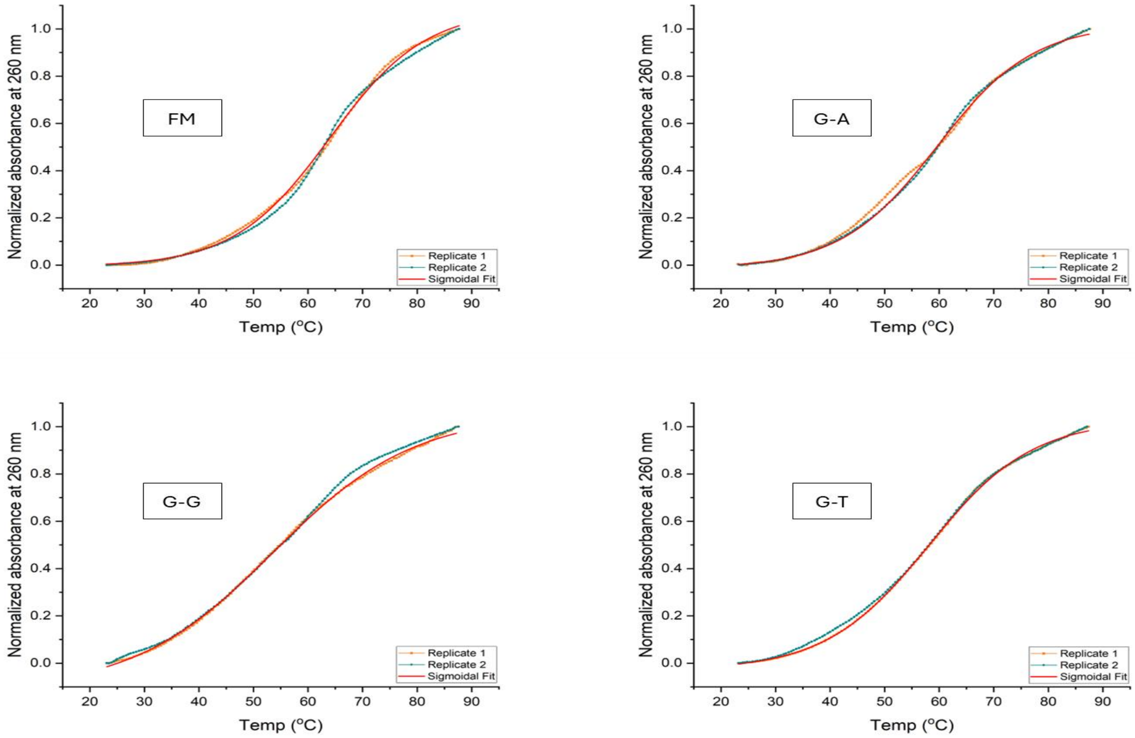

Figure S14: Melting curve profiles for cpG modified CCAT1 FIT-PNA annealed to complementary and mismatched synthetic DNA. [FIT-PNA] =[DNA] = 2µM.

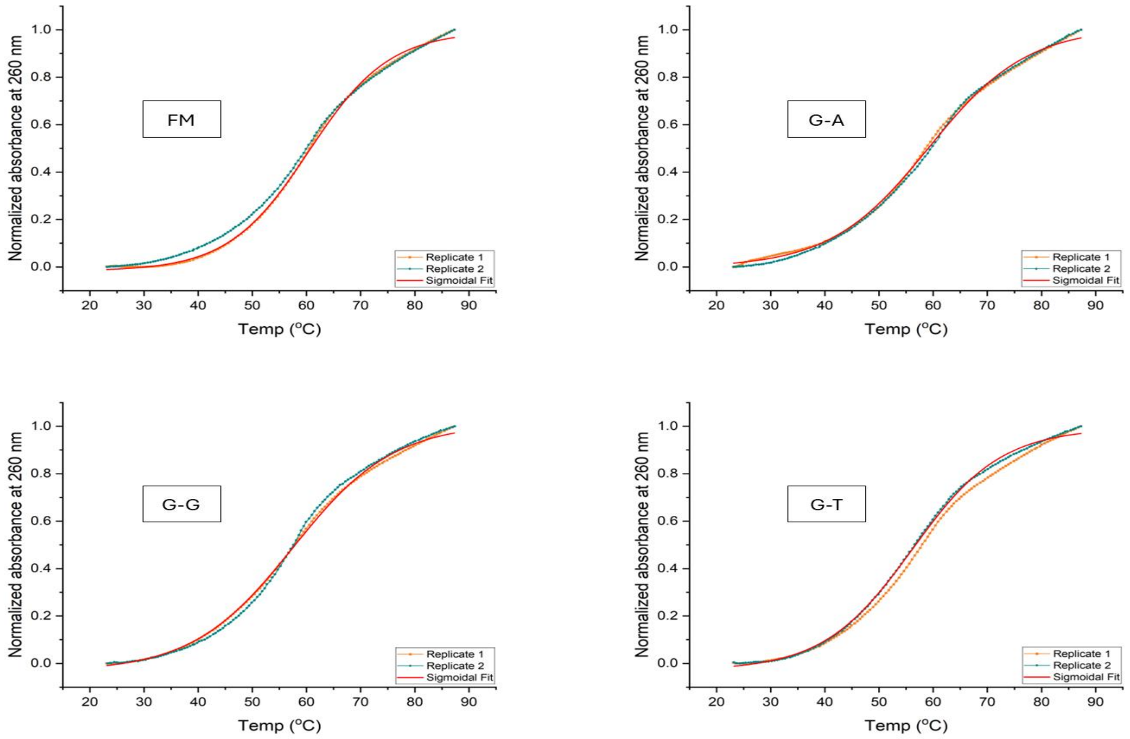

Figure S15: Melting curve profiles for cpG^{+} modified CCAT1 FIT-PNA annealed to complementary and mismatched synthetic DNA. [FIT-PNA] = [DNA] = 2µM.

Fluorescence spectra of mismatch sensitivity of modified and unmodified CCAT1 FIT-PNAs with RNA and DNA

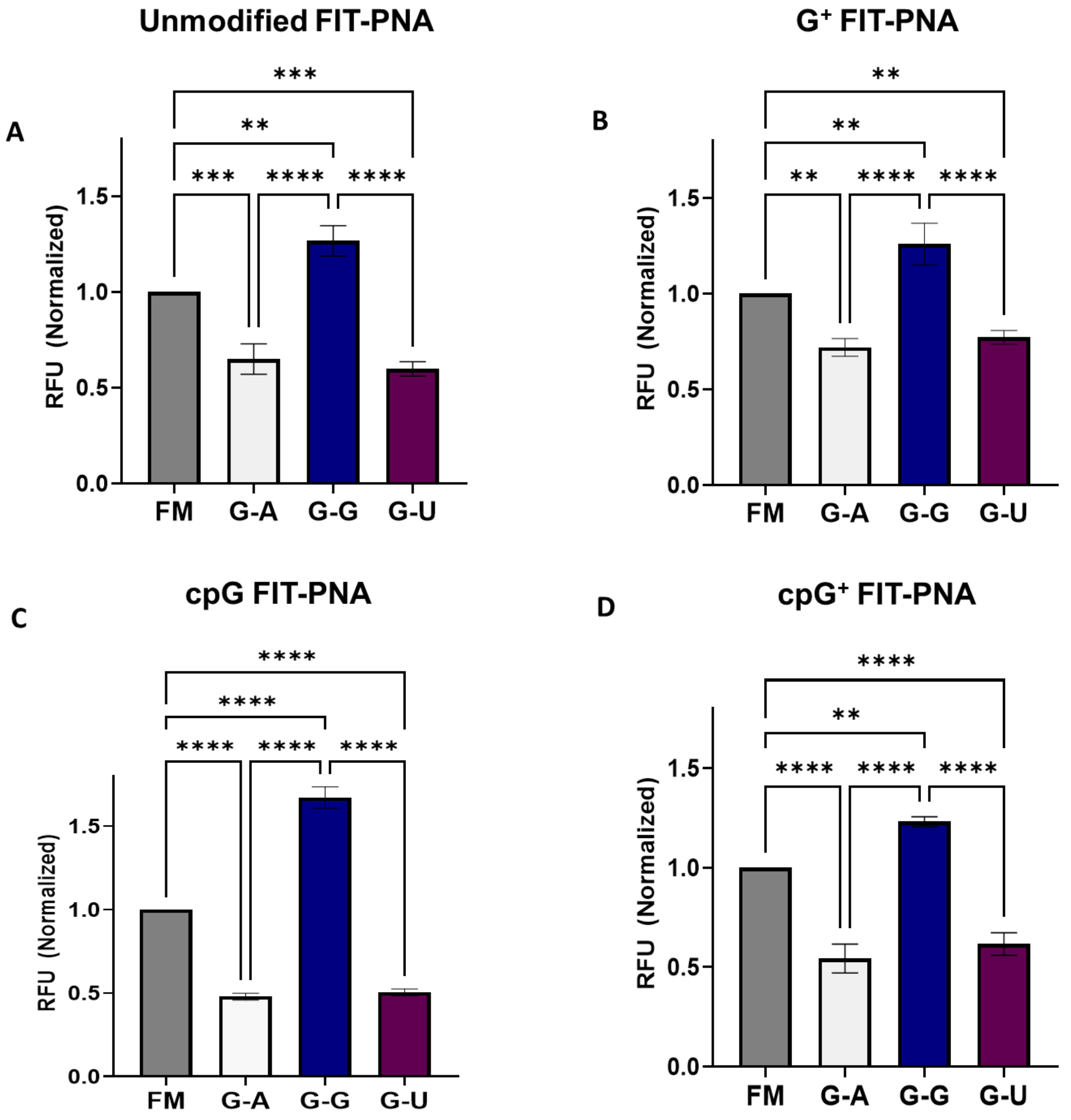

Figure S16: **Mismatch selectivity of modified and unmodified CCAT1 FIT-PNAs after RNA hybridization.** **(A)** Unmodified CCAT1 FIT-PNA, **(B)** G^+ modified CCAT1 FIT-PNA, **(C)** cpG modified CCAT1 FIT-PNA, **(D)** cpG^+ modified CCAT1 FIT-PNA. FIT-PNAs (0.5 µM) were annealed to matched or mismatched RNAs (1 µm) at 37 ºC for 2 hours. Duplex fluorescence of all FIT-PNAs is marked in grey for the fully matched RNA, in white for A mismatched RNA, blue for G mismatched RNA and purple for U mismatched RNA. (λex = 570 nm, λem = 580 nm). The Data is normalized to the fully matched RNA and presented as the mean ± SD (n = 3). *** represents $p \leq 0.001$, ** represents $p \leq 0.01$ and * represents $p \leq 0.05$ as determined by a One-way ANOVA test.

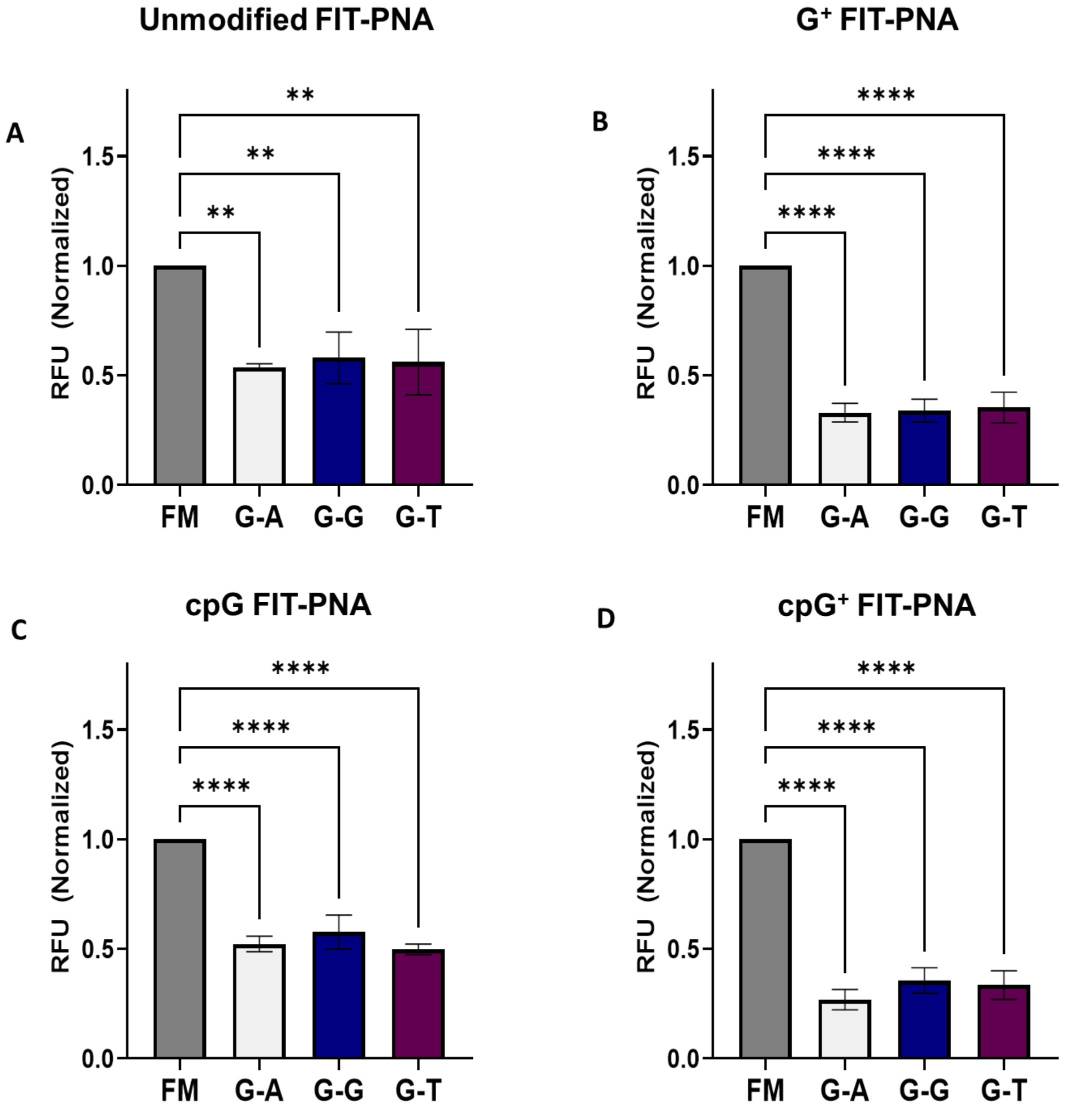

Figure S17: **Mismatch selectivity of modified and unmodified CCAT1 FIT-PNAs after DNA hybridization. (A)** Unmodified CCAT1 FIT-PNA, **(B)** G^+ modified CCAT1 FIT-PNA, **(C)** cpG modified CCAT1 FIT-PNA, **(D)** cpG^+ modified CCAT1 FIT-PNA. FIT-PNAs (0.5 µM) were annealed to matched or mismatched DNAs (1 µm) at 37 ºC for 2 hours. Duplex fluorescence of all FIT-PNAs is marked in grey for the fully matched DNA, in white for A mismatched DNA, blue for G mismatched DNA and purple for T mismatched DNA. (λex = 570 nm, λem = 580 nm). The Data is normalized to the fully matched DNA and presented as the mean ± SD (n = 3). *** represents $p \leq 0.001$, ** represents $p \leq 0.01$ and * represents $p \leq 0.05$ as determined by a One-way ANOVA test.

UV-Vis spectrum of CCAT1 FIT PNAs

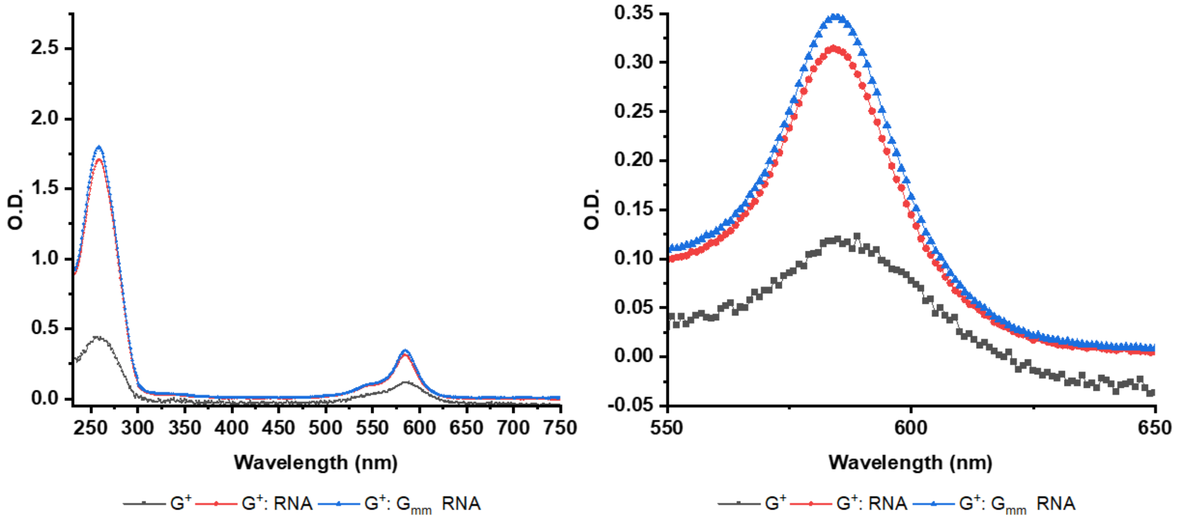

Figure S18: UV-Vis spectrum of G^{+} FIT-PNA with and without the presence of fully matched/ G_{mm} synthetic RNA. [FIT-PNA]= [RNA]= 4μM.

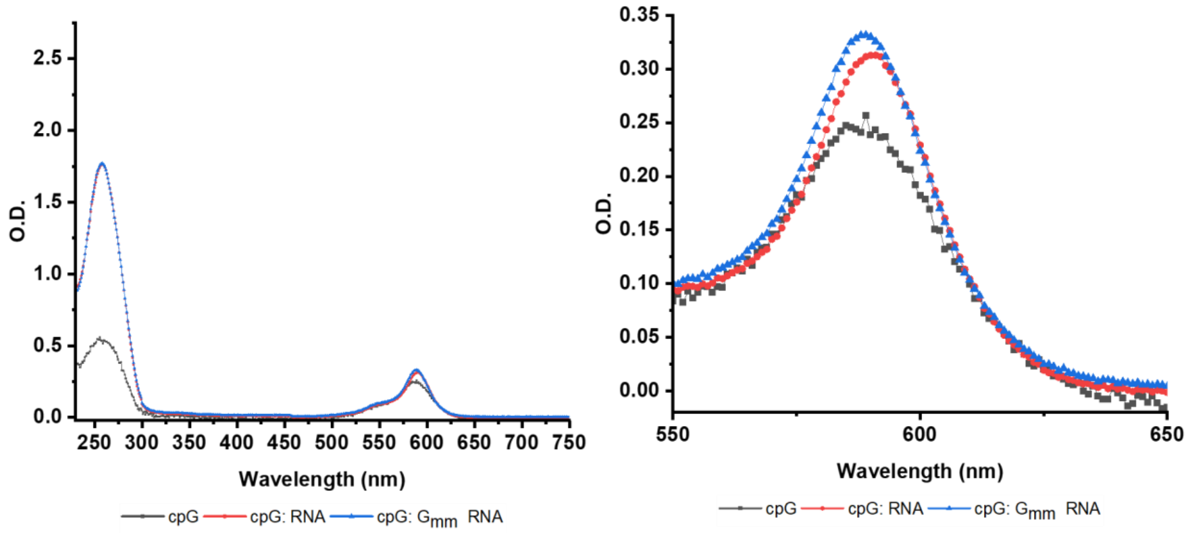

Figure S19: UV-Vis spectrum of cpG FIT-PNA with and without the presence of fully matched/ G_{mm} synthetic RNA. [FIT-PNA]= [RNA]= 4μM.

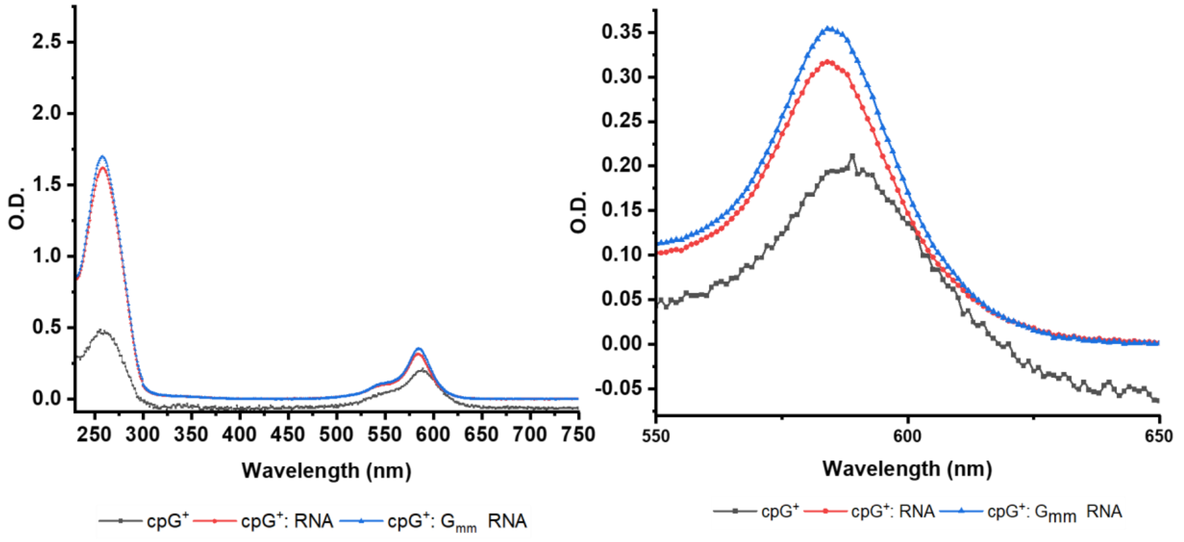

Figure S20: UV-Vis spectrum of cpG^{+} FIT-PNA with and without the presence of fully matched/ G_{mm} synthetic RNA. [FIT-PNA]= [RNA]= 4µM.

Circular dichroism (CD) spectroscopy

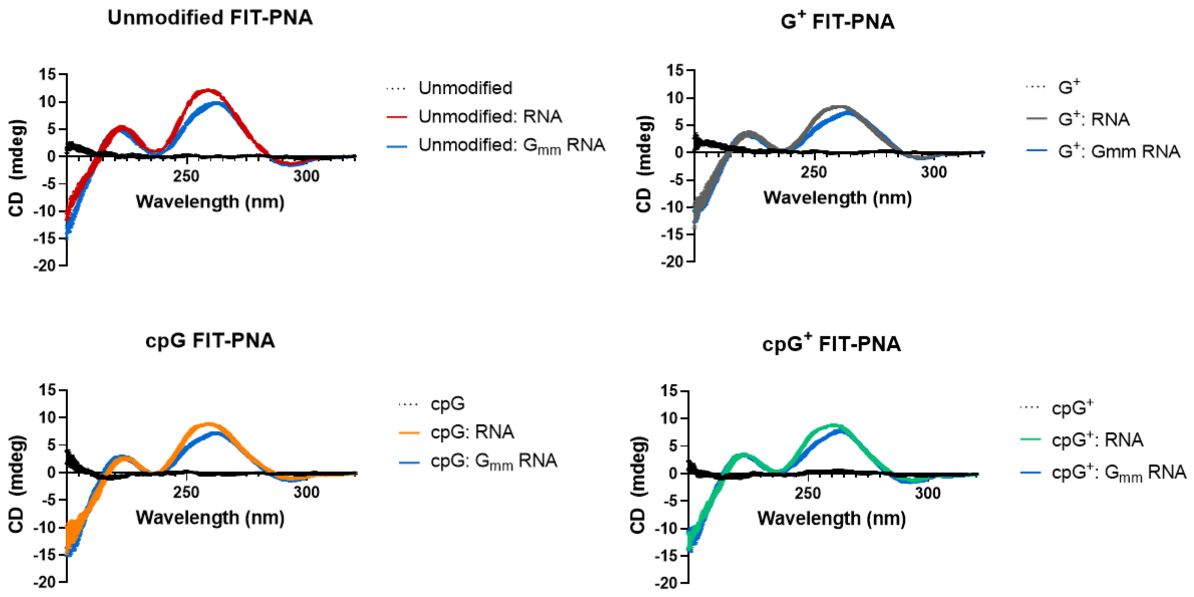

Figure S21: CD spectra of modified and unmodified CCAT1 FIT-PNAs as single strand and hybridized to fully matched and G_{mm} RNA in PBS buffer. [FIT-PNA]=[RNA]= 15µM.

Quantum yields of modified and unmodified CCAT1 FIT-PNAs

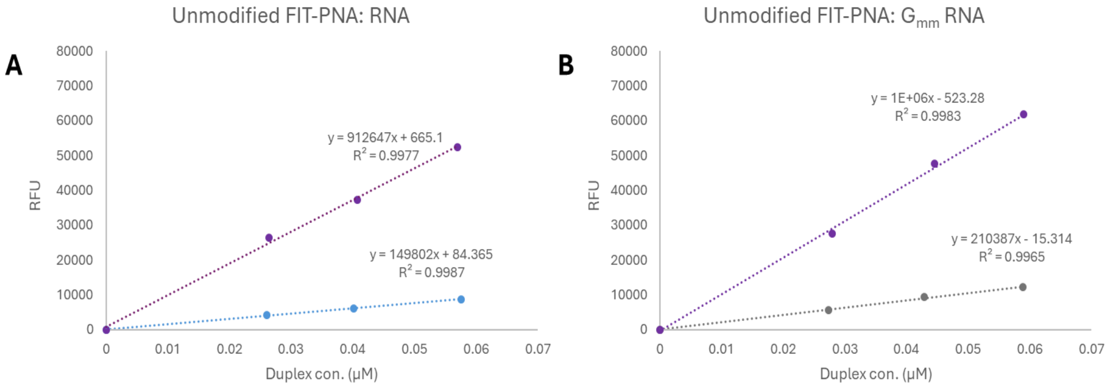

Figure S22: Quantum yield determination of unmodified FIT-PNA relative to cresyl violet with: **(A)** fully matched RNA and **(B)** G mismatched RNA in PBS (pH 7.0). FIT-PNA (4, 6 and 8 µM) was incubated with RNA at a ratio of 1:2 at 37°C for 2 hours. (λex = 580 nm, λem = 400-750 nm).

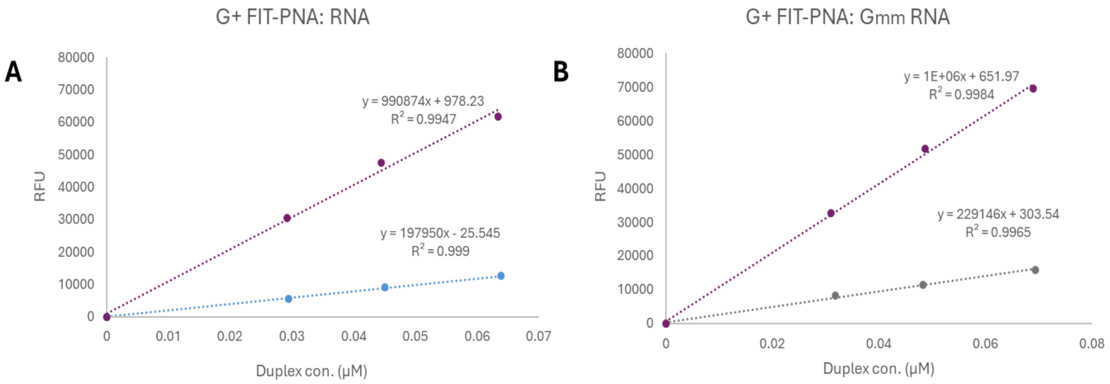

Figure S23: Quantum yield determination of G^+ FIT-PNA relative to cresyl violet with: **(A)** fully matched RNA and **(B)** G mismatched RNA in PBS (pH 7.0). FIT-PNA (4, 6 and 8 µM) was incubated with RNA at a ratio of 1:2 at 37°C for 2 hours. (λex = 580 nm, λem = 400-750 nm).

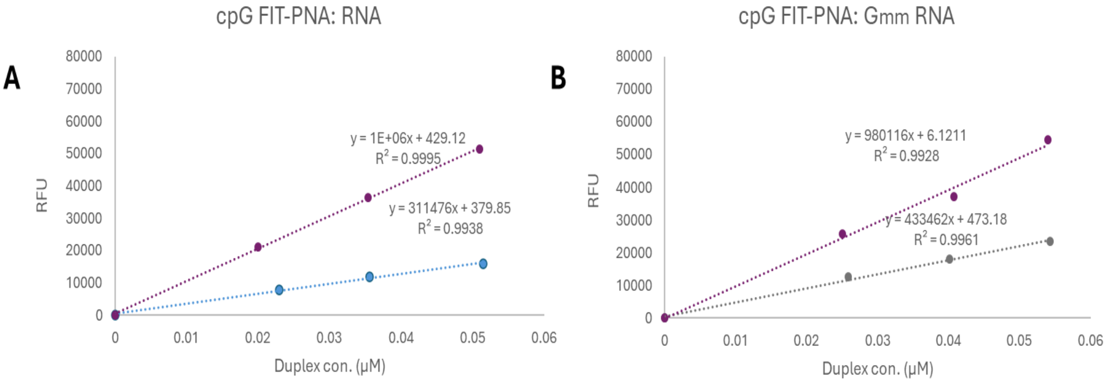

Figure S24: Quantum yield determination of cpG FIT-PNA relative to cresyl violet with: **(A)** fully matched RNA and **(B)** G mismatched RNA in PBS (pH 7.0). FIT-PNA (4, 6 and 8 µM) was incubated with RNA at a ratio of 1:2 at 37°C for 2 hours. (λex = 580 nm, λem = 400-750 nm).

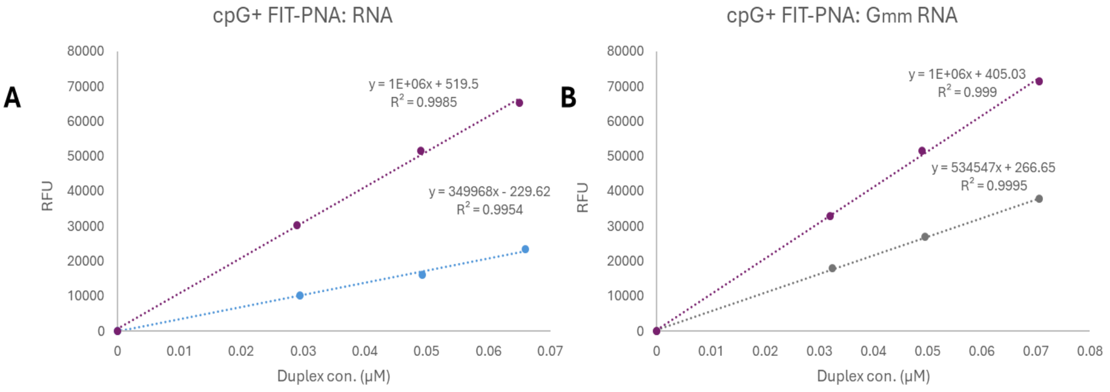

Figure S25: Quantum yield determination of cpG^{+} FIT-PNA relative to cresyl violet with: **(A)** fully matched RNA and **(B)** G mismatched RNA in PBS (pH 7.0). FIT-PNA (4, 6 and 8 µM) was incubated with RNA at a ratio of 1:2 at 37°C for 2 hours. (λex = 580 nm, λem = 400-750 nm)

Limit of Detection (LOD)

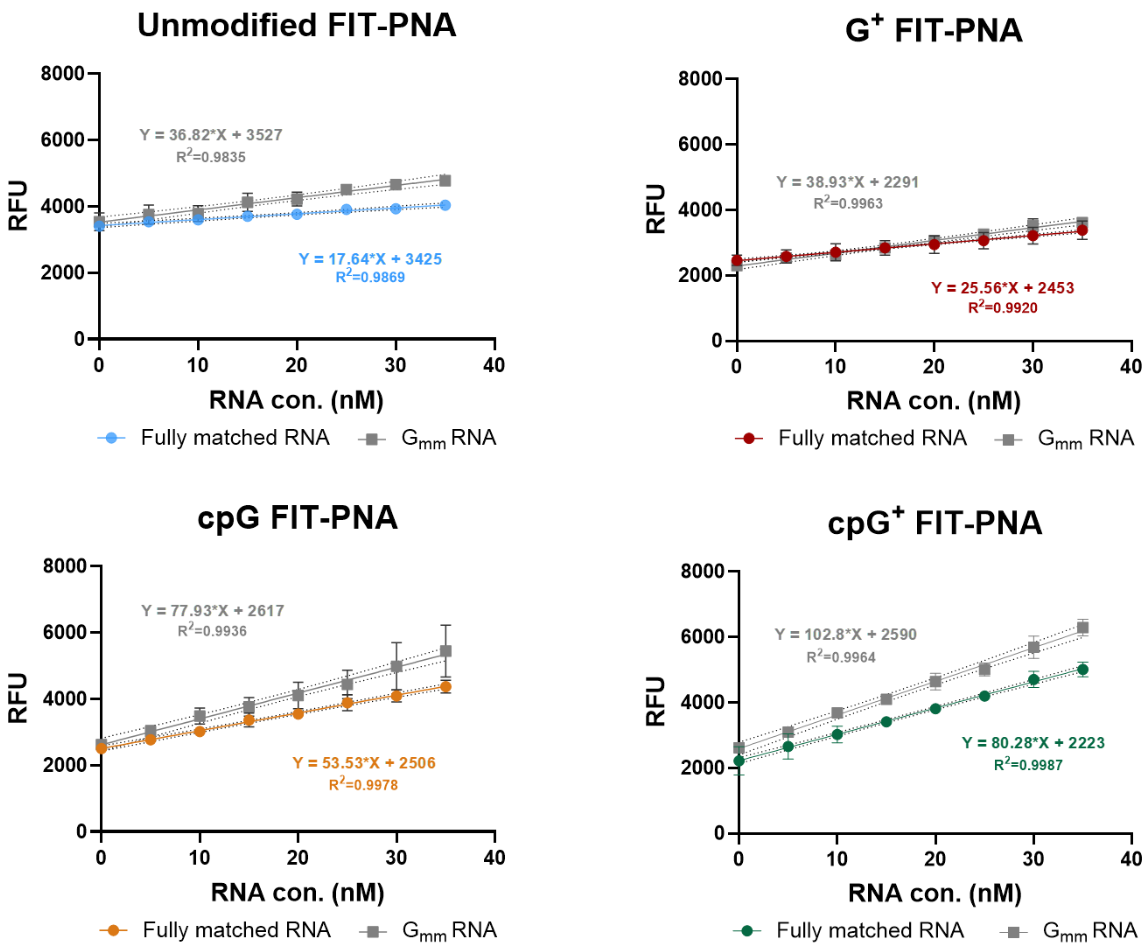

Figure S26: **LOD of modified and unmodified CCAT1 FIT-PNAs.** LOD was measured on a plate reader at constant PNA concentration of 0.5 µM and the complementary and G_{mm} RNA were added in different concentrations. For annealing, all duplex solutions were incubated at 37 ºC for 2 hours. (λex=580 nm, λem= 610 nm, n=3).

RT-qPCR

Table S4. Primer sequences used for the RT-qPCR.

Primers	Sequence (5'-3')
RPLPO-Forward	CCAACTACTTCCTTAAGATCATCCAACTA
RPLPO-Reverse	ACATGCGGATCTGCTGCA
CCAT1-Forward	GGCACTACTCTGTCCCAACA
CCAT1-Reverse	TAGCCATACAGAGCCAACCT

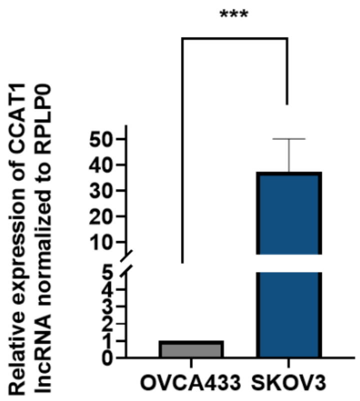

Figure S27: RT-qPCR analysis of CCAT1 lncRNA expression in OVCA433 and SKOV3 cells. Analysis performed using Student's t-test; error bars represent mean ± SEM; *** $p < 0.001$.

Flow Cytometry Analysis

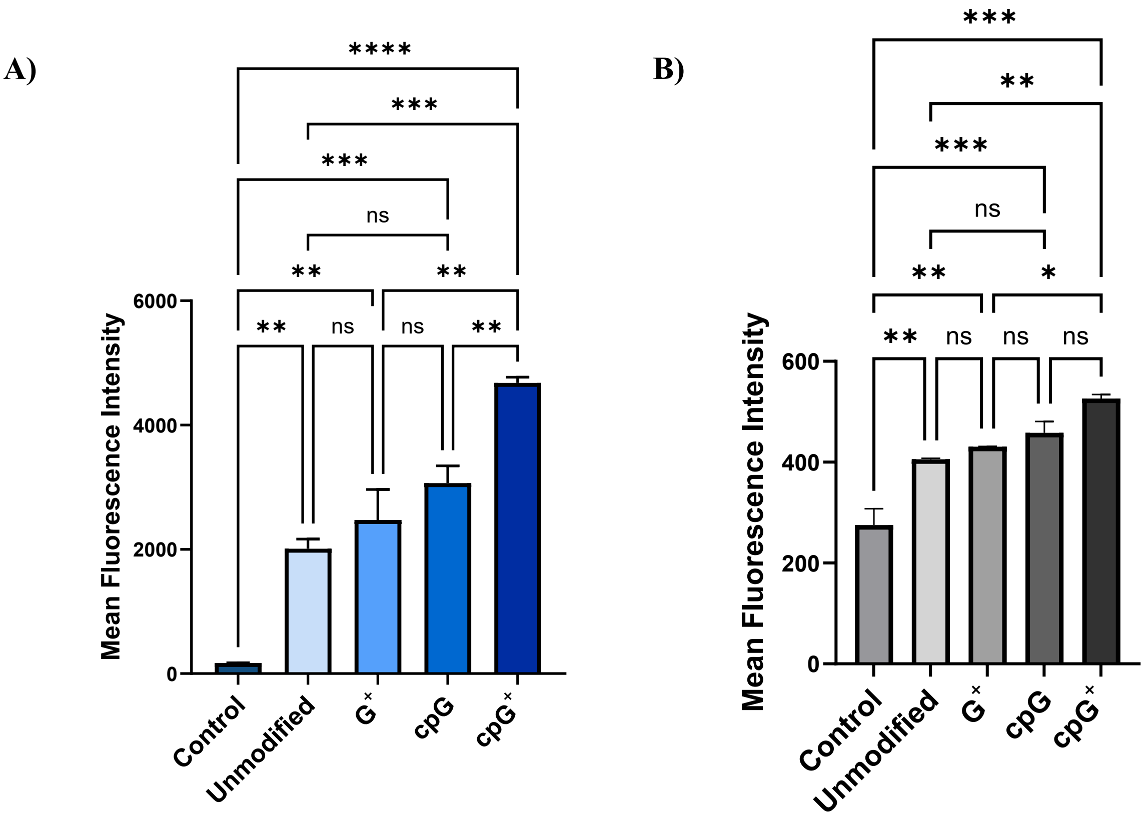

Figure S28: **Mean fluorescence intensity of OC cells. (A)** Mean fluorescence intensity of SKOV3 cells treated with 2 µm of modified and unmodified CCAT1 FIT-PNAs for 3h at 37 ^{0}C. **(B)** Mean fluorescence intensity of OVCA433 cells treated with 2 µm of modified and unmodified CCAT1 FIT-PNAs for 3h at 37 ^{0}C. The Data is presented as the mean ± SD (n = 2). *** represents $p \leq 0.001$, ** represents $p \leq 0.01$ and * represents $p \leq 0.05$ as determined by a One-way ANOVA test.

Supporting Information

A.

SKOV3-
Control

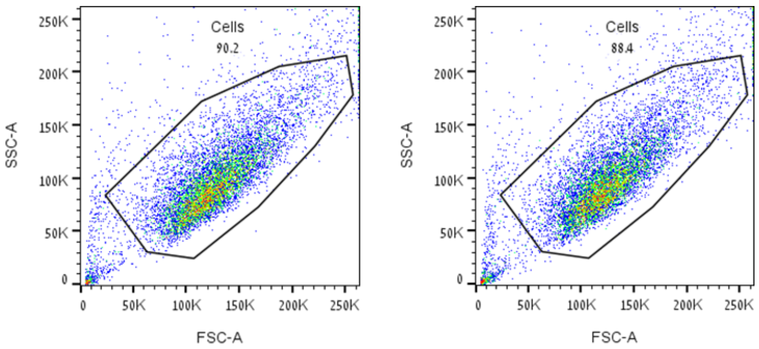

B.

SKOV3-
Unmodified

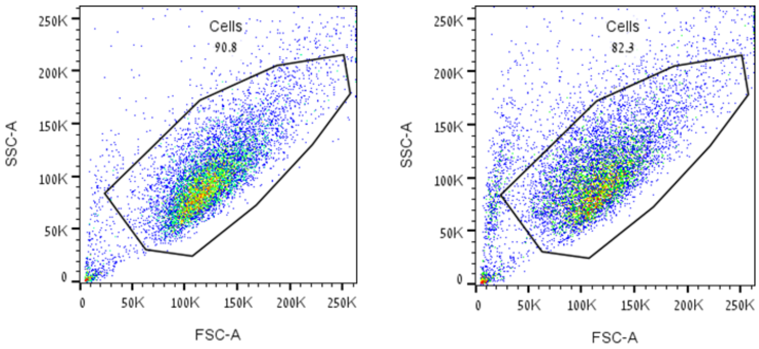

C.

SKOV3-
G+

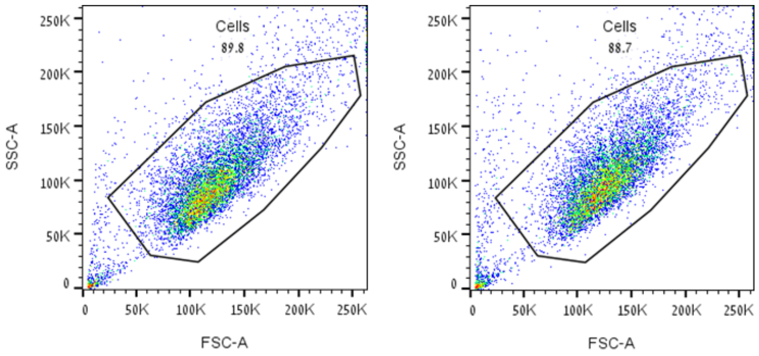

D.

SKOV3-
cpG

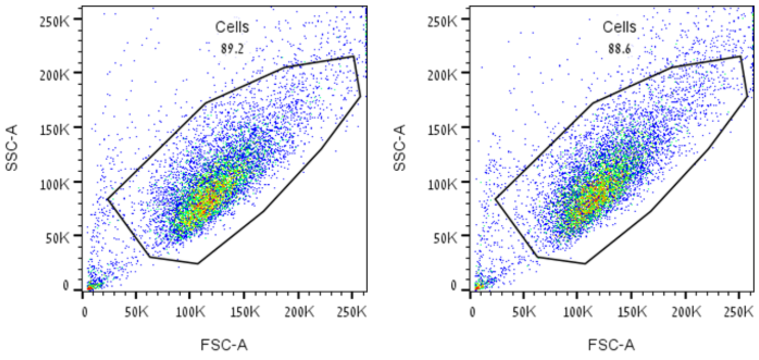

E.

SKOV3-cpG^{+}

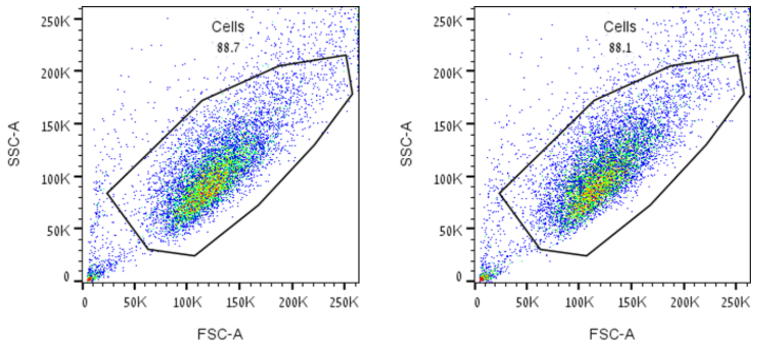

Figure S29: Forward and sideward scatter plots of SKOV3 cells incubated with 2 µM of FIT-PNA for 3 h at 37°C in media. **(A)** Untreated SKOV3 cells (served as control), **(B)** Unmodified FIT-PNA, **(C)** G^{+} FIT-PNA, **(D)** cpG FIT-PNA and **(E)** cpG^{+} FIT-PNA.

Supporting Information

A.

OVCA433-
Conrol

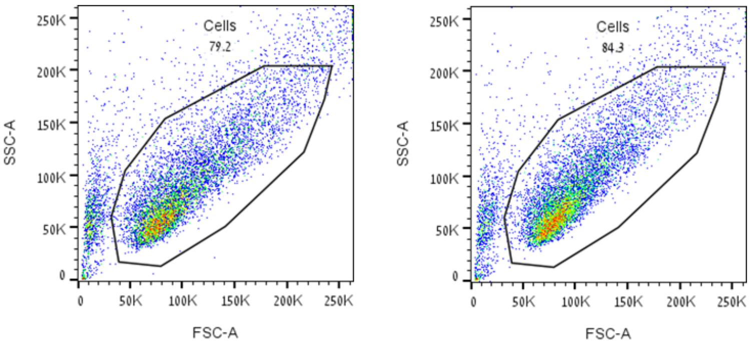

B.

OVCA433-
Unmodified

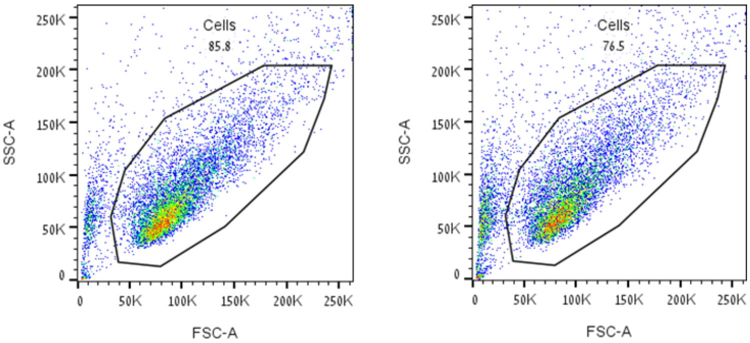

C.

OVCA433-
G^+

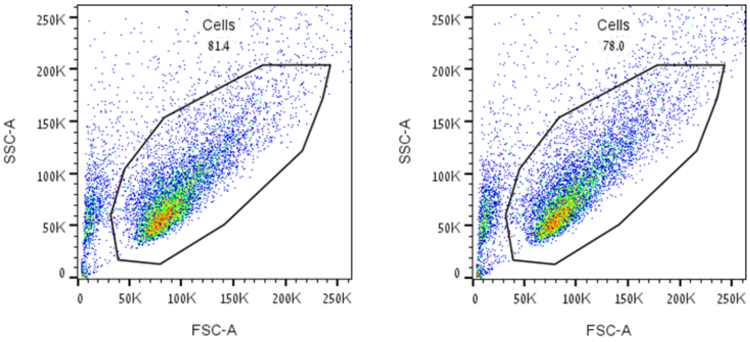

D.

OVCA433-
cpG

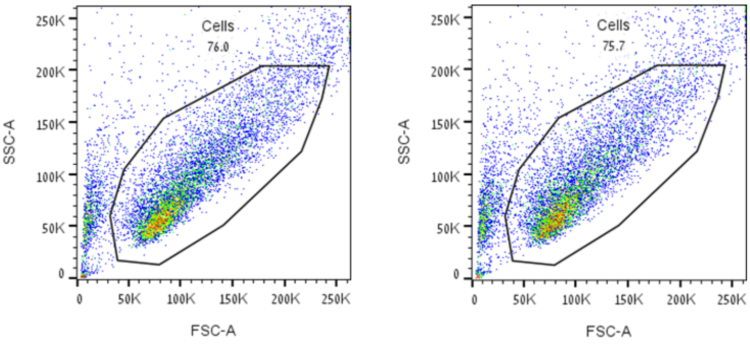

E.

OVCA433-cpG$^+$

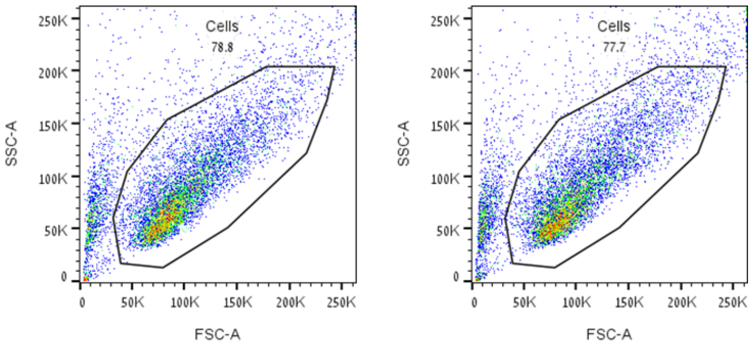

Figure S30: Forward and sideward scatter plots of OVCA433 cells incubated with 2 µM of FIT-PNA for 3 h at 37°C in media. **(A)** Untreated OVCA433 cells (served as control), **(B)** Unmodified FIT-PNA, **(C)** G$^+$ FIT-PNA, **(D)** cpG FIT-PNA and **(E)** cpG$^+$ FIT-PNA.

Supporting Information

A.

SKOV3-
Control

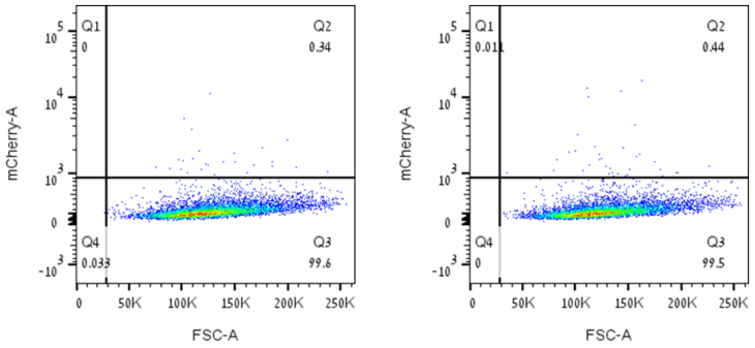

B.

SKOV3-
Unmodified

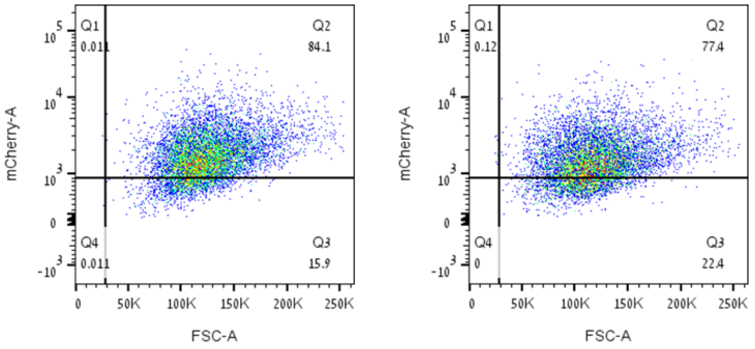

C.

SKOV3-
G^+

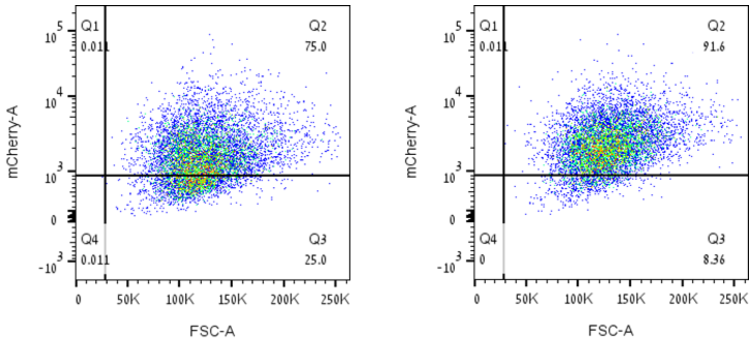

D.

SKOV3-
cpG

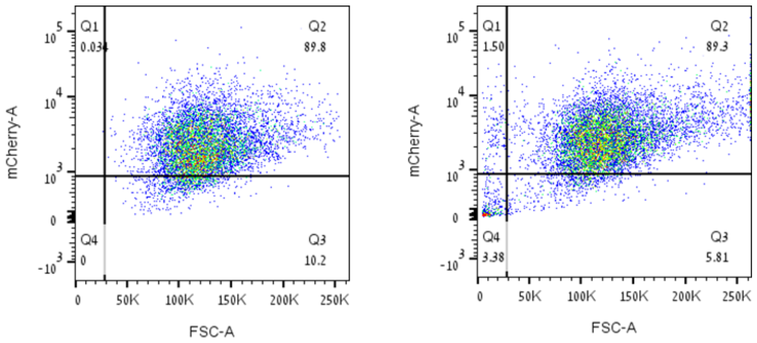

E.

SKOV3-cpG$^+$

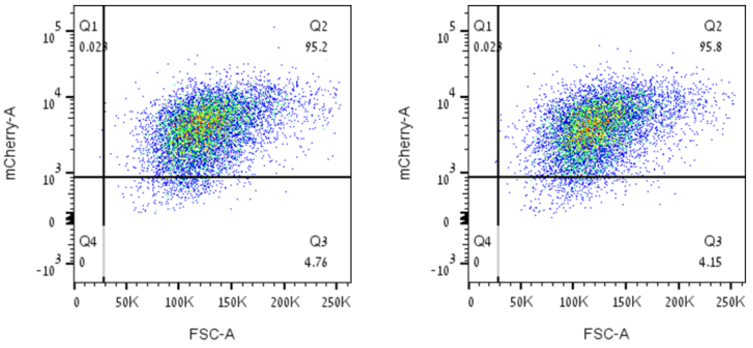

Figure S31: FACS results of replicates for FIT-PNAs in SKOV3 cells. The cells were incubated with 2 µM of FIT-PNA for 3 h at 37°C in media. The data is gated to the mCherry-positive populations. **(A)** Untreated SKOV3 cells (served as control), **(B)** Unmodified FIT-PNA, **(C)** G$^+$ FIT-PNA, **(D)** cpG FIT-PNA and **(E)** cpG$^+$ FIT-PNA.

Supporting Information

A.

OVCA433-Control

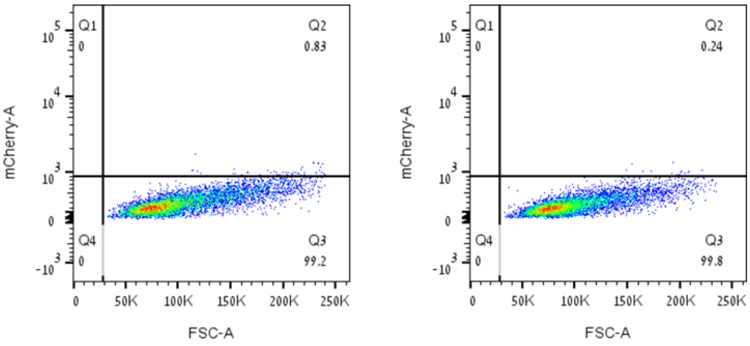

B.

OVCA433-Unmodified

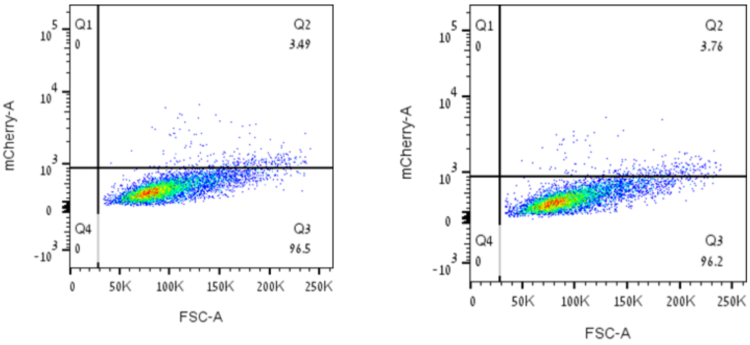

C.

OVCA433-G^+

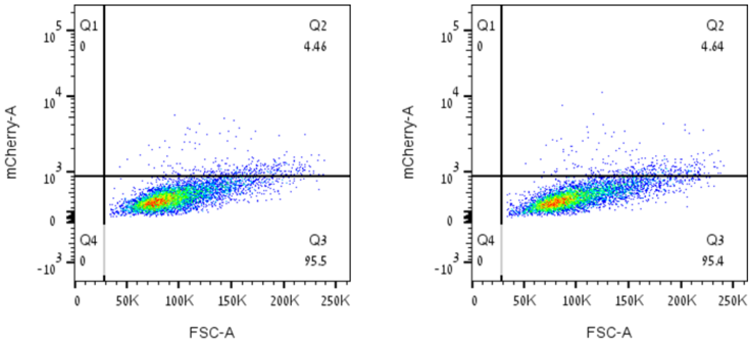

D.

OVCA433-cpG

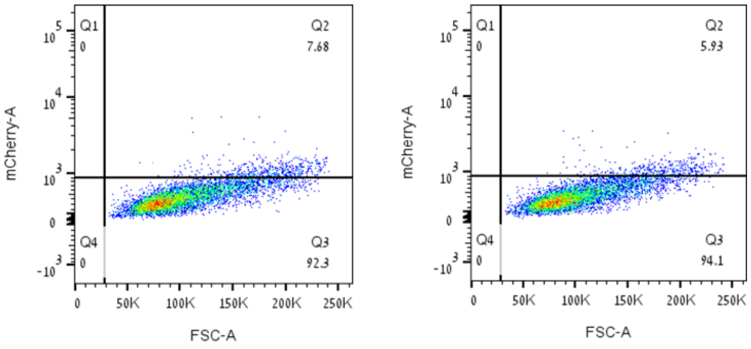

E.

OVCA433-cpG⁺

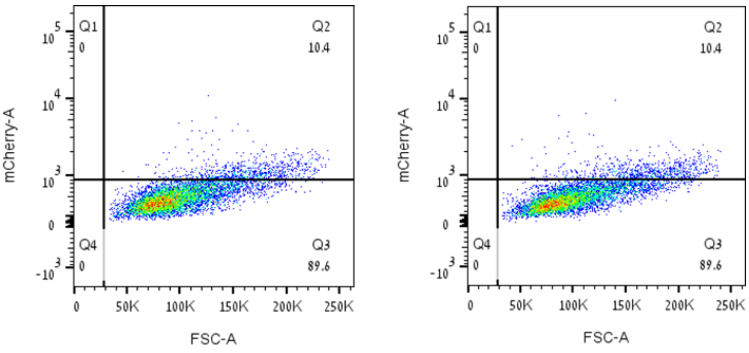

Figure S32: FACS results of replicates for FIT-PNAs in OVCA433 cells. The cells were incubated with 2 µM of FIT-PNA for 3 h at 37°C in media. The data is gated to the mCherry-positive populations. **(A)** Untreated OVCA433 cells (served as control), **(B)** Unmodified FIT-PNA, **(C)** G^+ FIT-PNA, **(D)** cpG FIT-PNA and **(E)** cpG^+ FIT-PNA.

Confocal Microscopy

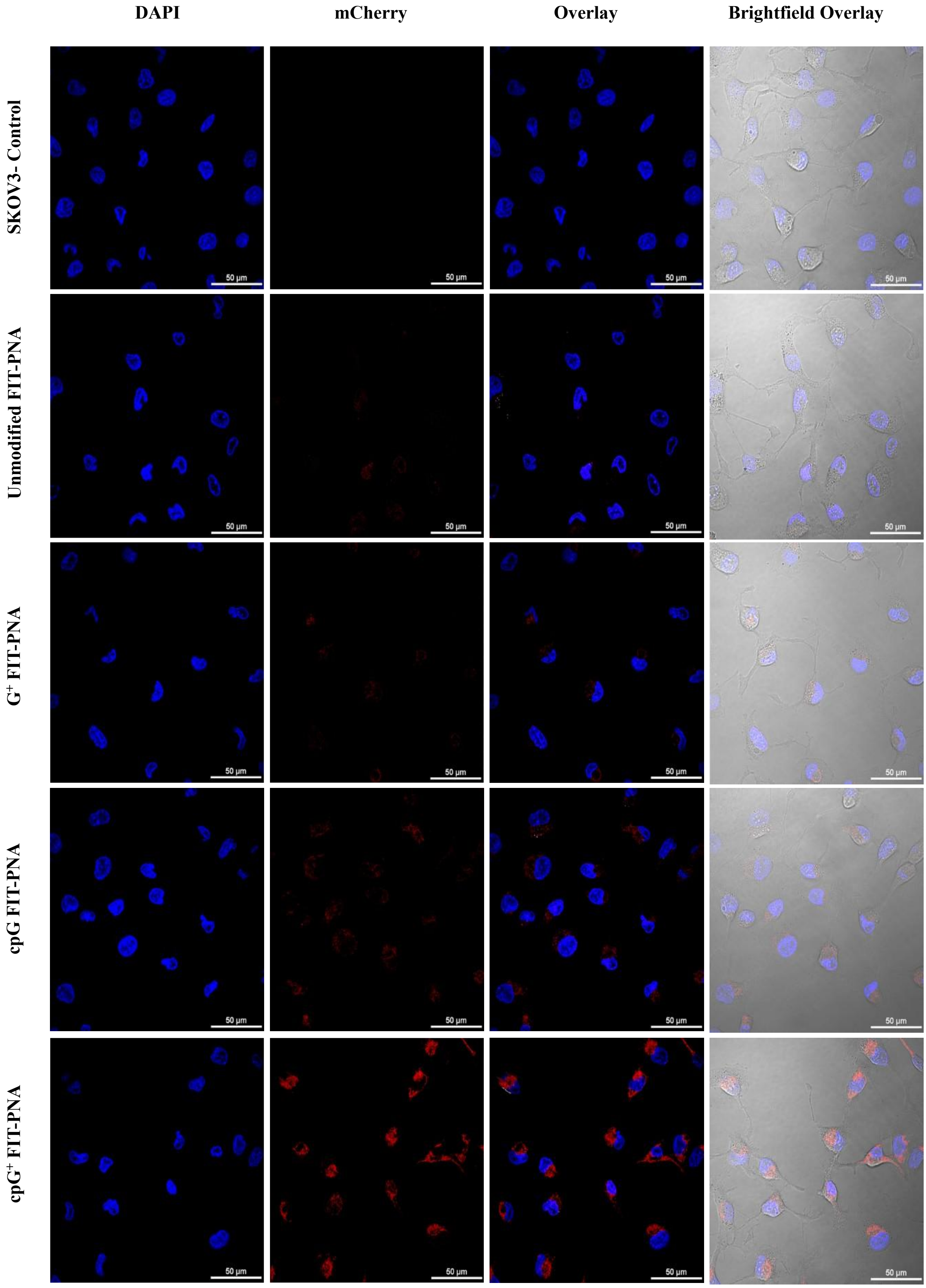

Figure S33: Confocal images of modified and unmodified CCAT1 FIT-PNAs (red) in SKOV3 cells with the nucleus labelled by DAPI marker (blue). Scale bar = 50 µm. Cells were treated with 2 µM of FIT-PNAs for 5 h at 37°C. Untreated cells served as control.

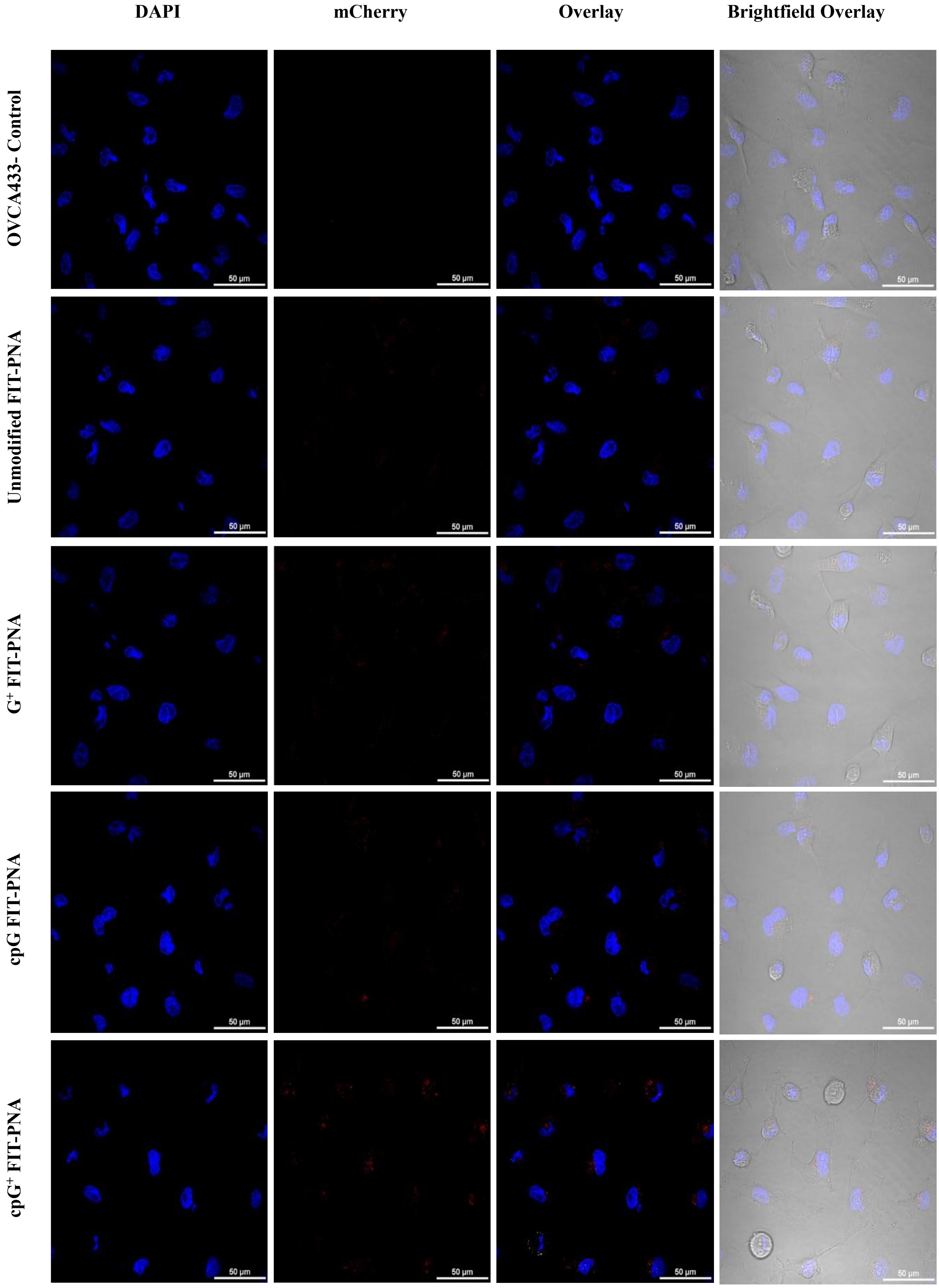

.

Figure S34: Confocal images of modified and unmodified CCAT1 FIT-PNAs (red) in OVCA433 cells with the nucleus labelled by DAPI marker (blue). Scale bar = 50 µm. Cells were treated with 2 µM of FIT-PNAs for 5 h at 37°C. Untreated cells served as control.

^{1}H and ^{13}C NMR spectra and HRMS of cpG^{+} PNA monomer:

cpG^{+} PNA monomer was synthesized following the same protocol used for G^{+} PNA monomer (1).

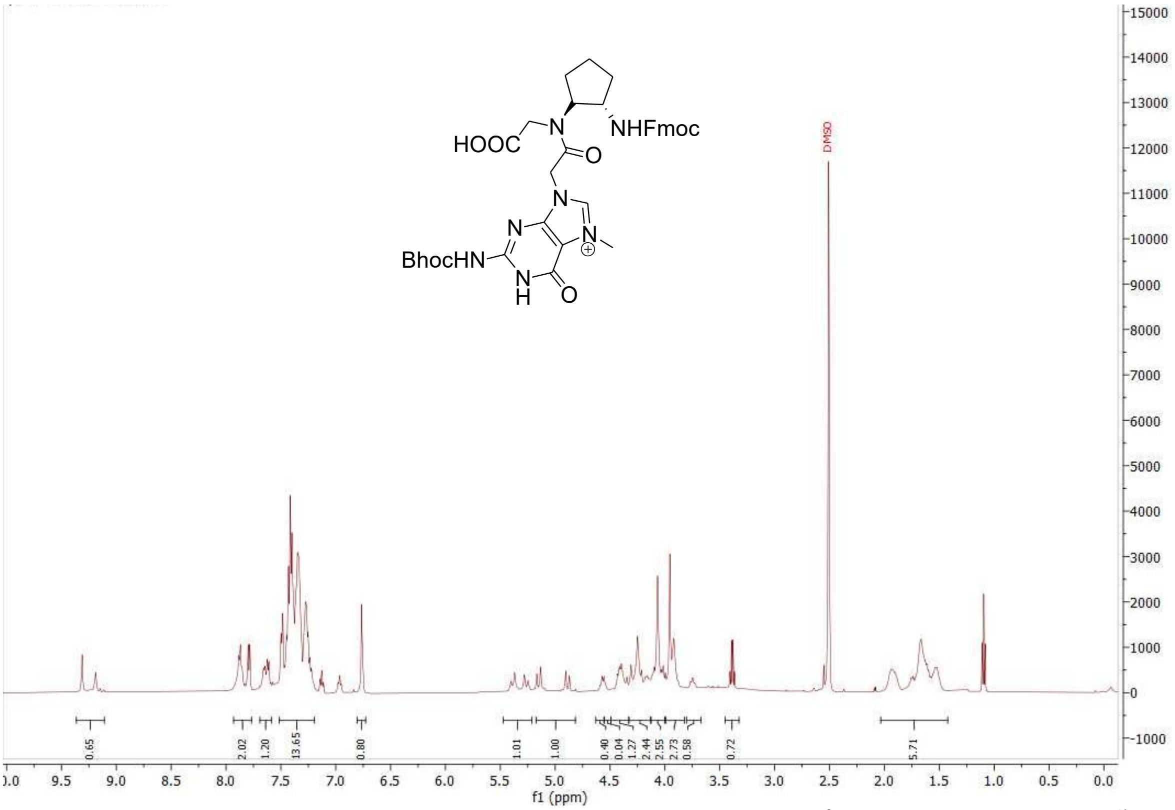

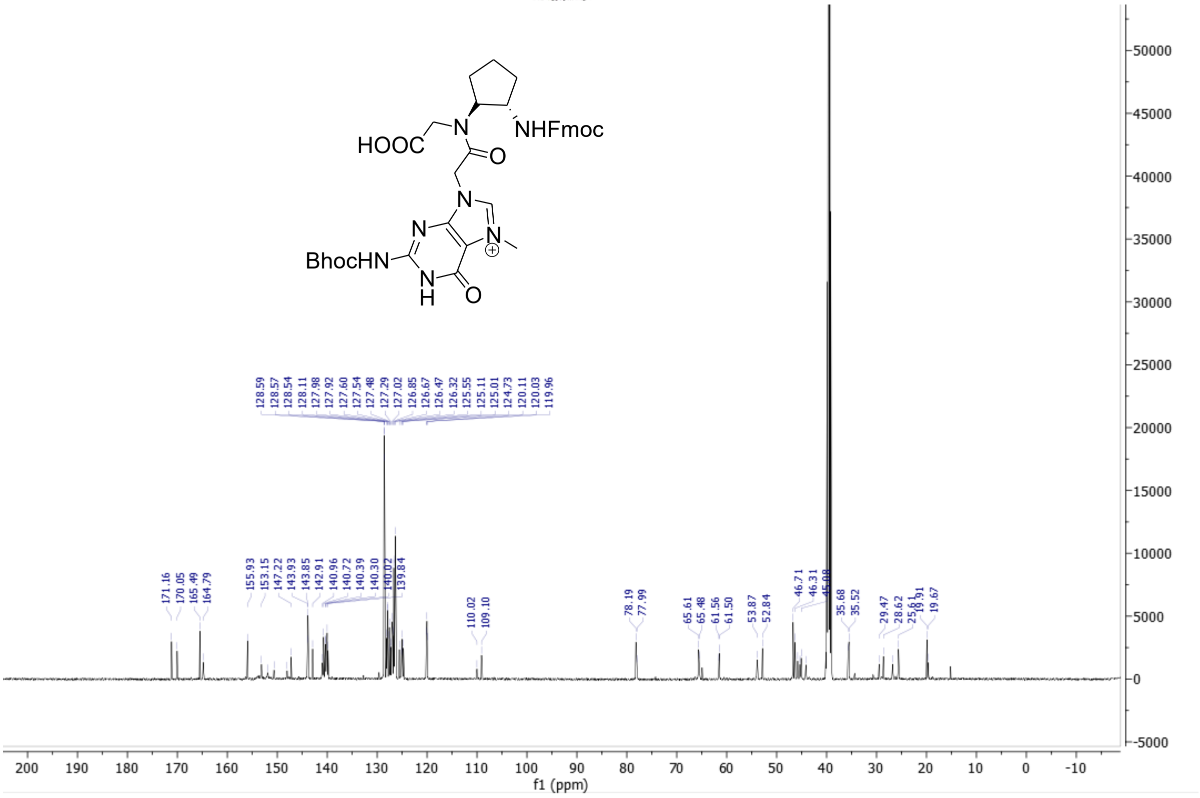

Figure S35: ^{1}H and ^{13}C NMR spectra of cpG^{+} PNA monomer**.** ^{1}**H NMR** (500 MHz, d-DMSO) δ 9.36 – 9.11 (m, 1H), 7.93 – 7.77 (m, 2H), 7.69 – 7.59 (m, 1H), 7.51 – 7.20 (m, 14H), 6.76 (d, J = 4.5 Hz, 1H), 5.47 – 5.21 (m, 1H), 5.17 – 4.82 (m, 1H), 4.57 (t, J = 9.1 Hz, 1H), 4.54 (s, 0H), 4.49 – 4.33 (m, 1H), 4.33 – 4.13 (m, 2H), 4.13 – 4.00 (m, 2H), 3.94 (d, J = 15.7 Hz, 3H), 3.75 (t, J = 9.7 Hz, 1H), 3.39 (q, J = 7.0 Hz, 1H), 2.03 – 1.42 (m, 6H); ^{13}**C NMR** (126 MHz,d- DMSO) δ 171.16, 170.05, 165.49, 164.79, 155.93, 153.15, 151.91, 150.66, 148.03, 147.22, 143.93, 143.85, 142.91, 140.96, 140.72, 140.39, 140.30, 140.02, 139.84, 128.59, 128.57, 128.54, 128.11, 127.98, 127.92, 127.60, 127.54, 127.48, 127.29, 127.02, 126.85, 126.67, 126.47, 126.32, 125.55, 125.11, 125.01, 124.73, 120.11, 120.03, 119.96, 110.02, 109.10, 78.19, 77.99, 65.61, 65.48, 61.56, 61.50, 53.87, 52.84, 46.71, 46.31, 45.78, 45.33, 45.08, 44.14, 39.52, 35.68, 35.52, 29.47, 28.62, 26.78, 25.61, 19.91, 19.67.

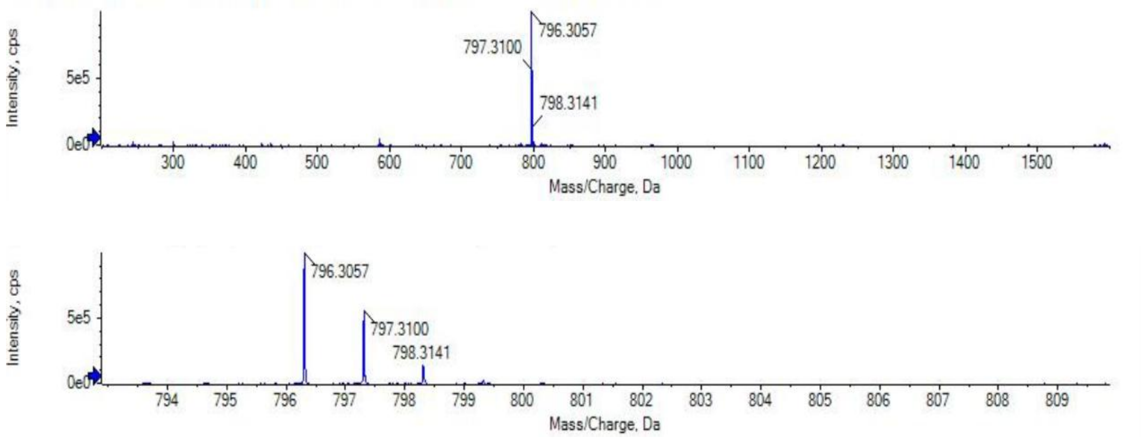

Figure S36: HRMS of cpG^{+} PNA monomer. M_{calc} = 796.30894, M_{obs} =796.30565.

Molecular Simulations

Building PNA:RNA and PNA:DNA duplex

Initially, Proto Nucleic Acid Builder (pNAB) was used to build the double stranded PNA molecule. Here we have used the 5' to 3' RNA/DNA strand sequence (from the target PNA:RNA/PNA:DNA sequence we want to build) as $N_{terminal}$ to $C_{terminal}$ PNA input sequence (Figure S37 – Step: 1a) (2). All parameters optimized for PNA building were set to default except the 'number of iterations to search over dihedral angles', which was set to a value of 10^9. Generated PNA:PNA duplex structure was uploaded to x3DNA server to calculate the helical parameters (3). At this point, the parameter file, which was generated by x3DNA, was modified manually (all the 'T' were replaced by 'U') and used to build double stranded RNA structure using x3DNA server and for generating double stranded B-DNA. The parameter file is used as is in the x3DNA server (Figure S37 – Step: 1b). From the generated PDB files of PNA:PNA duplex by pNAB and RNA:RNA/DNA:DNA duplex by x3DNA, one relevant stand from each PDB file were isolated for our final RNA:PNA/DNA:PNA duplex and saved as different PDB files (Figure S37– Step: 2a, 2b). These two PDB files, one containing single stranded PNA and another containing single stranded RNA/DNA were docked using HNADOCK server to get the PNA:RNA and PNA:DNA duplex (Figure S37 – Step: 3) (4). These PNA:RNA/PNA:DNA duplex structures were imported to Schrodinger Maestro software (v.14.0) and hydrogens, partial charges and bond orders were fixed (5, 6). Next, PNA:RNA and PNA:DNA duplex structures were energy minimized using OPLS4 force field build in Schrodinger Maestro software (v.14.0) to get the final structure (Figure S37 – Step: 4).

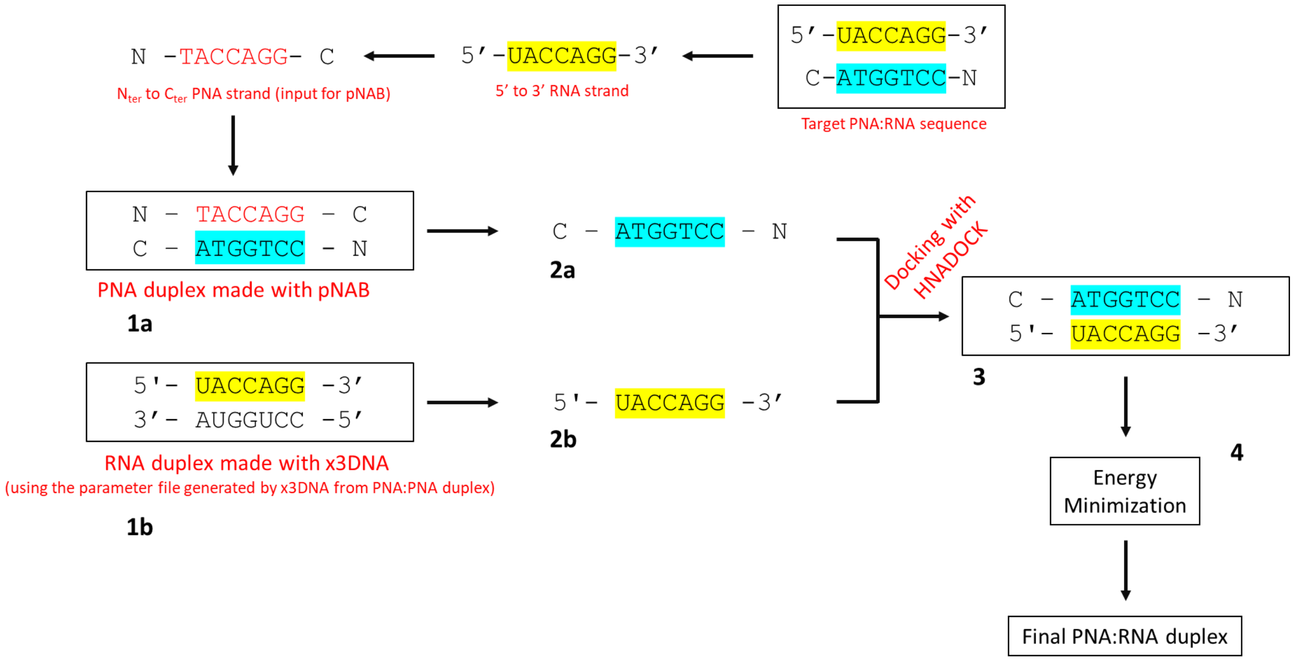

Figure S37: Pipeline for generating PNA:RNA duplex structure. This diagram shows how we generated 5'-UACCAGG-3' (RNA) and C-ATGGTCC-N (PNA) duplex 3D model starting from the sequence.

We have also generated an 8-mer PNA:RNA (5EMF) and an 8 mer PNA:DNA duplex (1PDT) from a published structure in the Protein Data Bank using the above-mentioned pipeline and compared the generated model with the published PDB structures to compare the accuracy of our method. After aligning crystal structures with our modelled structure, we found a RMSD value of 1.467 for 5EMF and 1.516 for 1PDT (Figure S38). We also found that most hydrogen bonds between nitrogen bases and π–π stacking interactions are conserved in our model (data not shown). Subsequently, we generated two original 7-mer PNA:RNA and PNA:DNA used in this study (Table S5: Sl. No. 1 and 2).

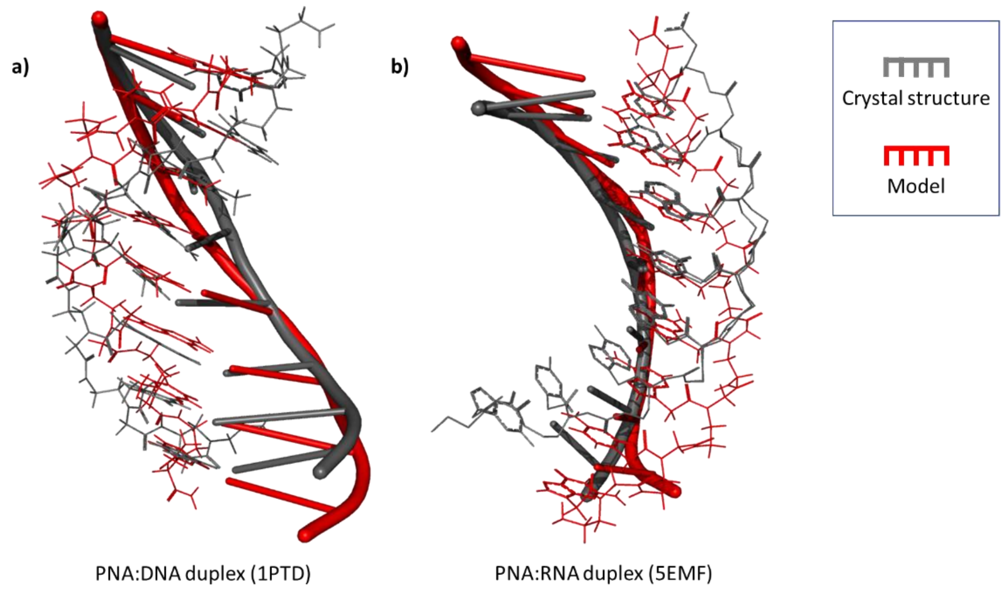

Figure S38: Comparison between published crystal structures (Gray) and our model (Red). RNA strands are represented by cartoon model and PNA strands are represented by line model. **(a)** 1PTD – PNA:DNA duplex. **(b)** 5EMF – PNA:RNA duplex.

Building BisQ FIT-PNA:RNA and BisQ FIT-PNA:DNA duplex with a G-G mismatch

We have modelled BisQ containing four different duplexes for our further studies (Table 5: Sl. No. 3 - 6). Before the final energy minimization step (Figure S37 – Step: 4), the 4th G base of the PNA from PNA:RNA/DNA duplex structures was removed and manually replaced by BisQ, followed by energy minimization to get the 'Fully matched BisQ FIT-PNA:RNA duplex' (Figure S39 a) and 'Fully matched BisQ FIT-PNA:DNA duplex' (Figure S40 a). In the same manner, before the final energy minimization step, the 4th G base of the PNA from PNA:RNA/DNA duplex structures was manually replaced by BisQ and the 3th C base (from the 5' end) of the DNA/RNA was changed to G followed by energy minimization to obtain 'G-G mismatched BisQ FIT-PNA:RNA duplex' (Figure S39 b) and 'G-G mismatched BisQ FIT-PNA:DNA duplex' (Figure S40 b).

Table S5. Sequences of all the duplex models generated for this study.

Sl. No.	Duplex sequence	Duplex type
1.	5′-UACCAGG- 3′<br>C -ATGGTCC- N	Original PNA:RNA duplex
2.	5′-TACCAGG- 3′<br>C -ATGGTCC- N	Original PNA:DNA duplex
3.	5′-UACCAGG- 3′<br>C -ATG**B**TCC- N	Fully matched BisQ FIT-PNA:RNA duplex<br>(B = BisQ)
4.	5′-UAGCAGG- 3′<br>C -ATG**B**TCC- N	G-G mismatched BisQ FIT-PNA:RNA duplex<br>(B = BisQ)
5.	5′-TACCAGG- 3′<br>C -ATG**B**TCC- N	Fully matched BisQ FIT-PNA:DNA duplex<br>(B = BisQ)
6.	5′-TAGCAGG- 3′<br>C -ATG**B**TCC- N	G-G mismatched BisQ FIT-PNA:DNA duplex<br>(B = BisQ)

In each case, BisQ was drawn using Maestro 'Add Fragment' tool in such a way that both quinoline ring of BisQ form π–π stacking with neighboring nucleobases and “mutation” of C base to G base were performed using built-in mutation option in Maestro. Energy minimization was done in Maestro using OPLS4 force field (7). We used PRCG method with maximum iteration of 5000 and gradient convergence threshold was set to 0.05. During minimization process of BisQ containing FIT-PNA:RNA/DNA duplexes, one base pair from each terminal was kept intact by constraining the distance between the heavy atoms responsible for the formation of those hydrogen bonds and other base pairs were allowed free movement assuming that BisQ can disrupt the geometry of base pairing H-bonds to the neighboring base pairs. All four BisQ containing FIT-PNA:RNA/DNA duplexes were used to run further simulations.

Stochastics Dynamics Simulation

The free rotation of the dihedral angle between the two quinoline rings of BisQ limits its ability to stack between nucleobases and furthermore, this rotation is also responsible for fluorescent quenching. In this study, we ran 10 ns Stochastics Dynamics simulation (Time step 1.5 ps and temperature 300K) of the prepared BisQ FIT-PNA:RNA/DNA duplex (fully matched and G-G mismatched) structures using OPLS4 force field with an equilibration time of 500 ps. During simulation, the same method of constraining the terminal bases and allowing free movement of all other bases was applied. We have monitored the dihedral angle between two ring systems of BisQ. A total of 1000 structures were generated in equal time intervals for further analysis of the π–π stacking over time of the BisQ molecule in the BisQ FIT-PNA:RNA/DNA duplexes.

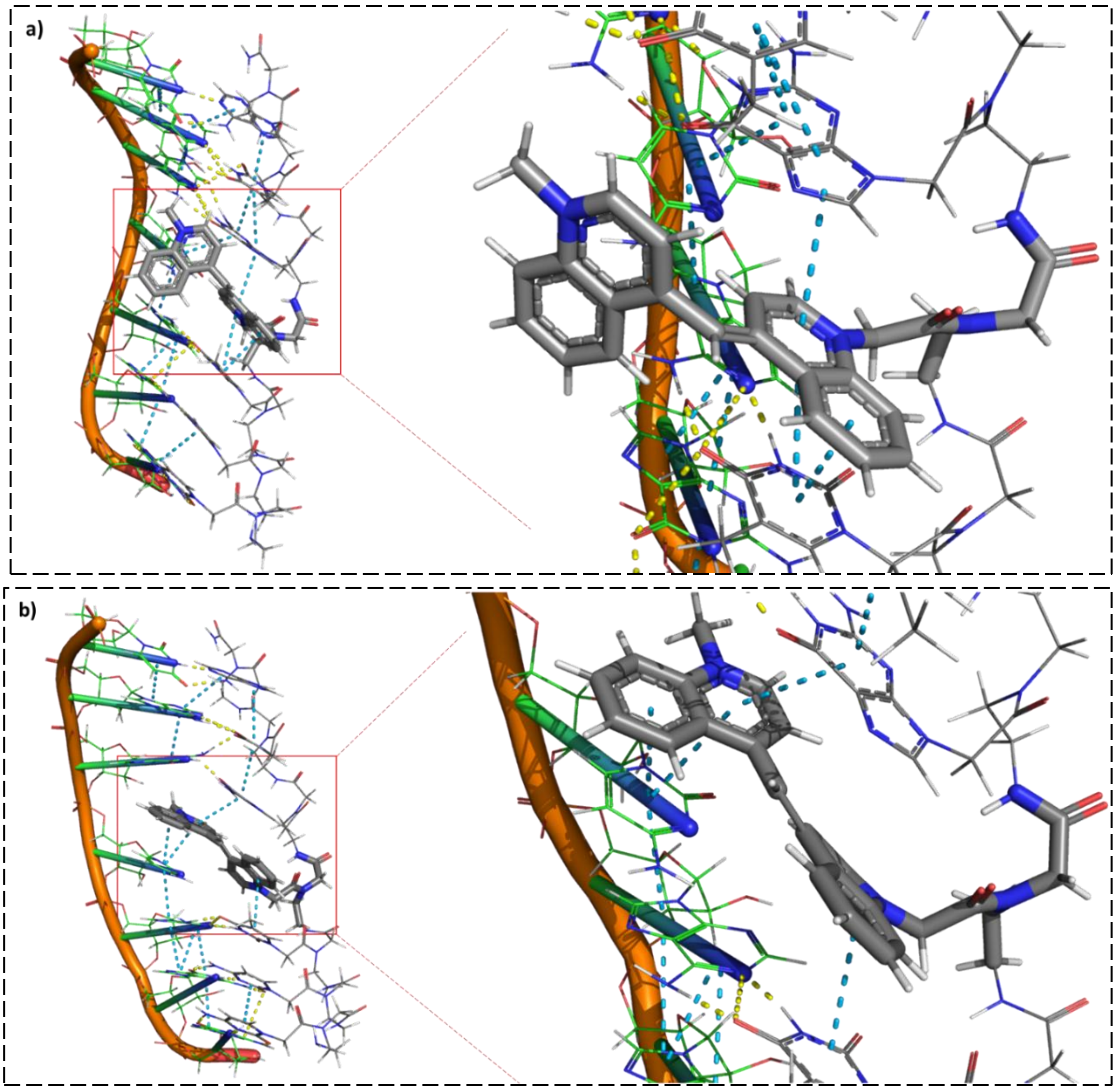

Figure S39: Generated 7-mer BisQ FIT-PNA:RNA duplex model used in this study. (a) Fully matched duplex, (b) G-G mismatched duplex. PNA is represented by grey line model, BisQ is represented by grey stick model, RNA is represented by both green line and cartoon model, π–π stacking is represented by cyan dashed line and H-bond are represented by yellow dashed line.

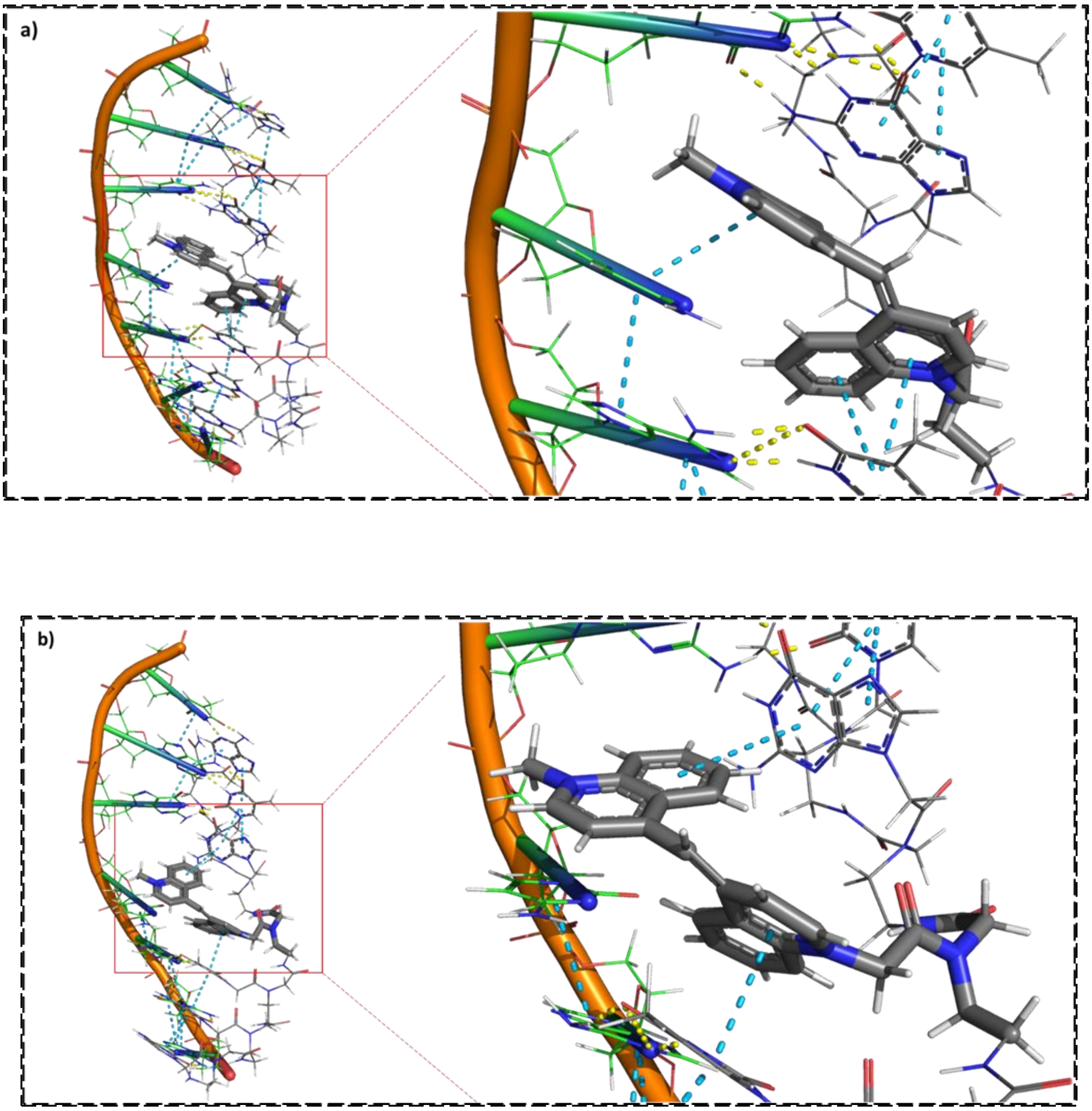

Figure S40: Generated 7-mer BisQ FIT-PNA:DNA duplex model used in this study (a) Fully matched duplex (b) G-G mismatched duplex. PNA is represented by grey line model, BisQ is represented by grey stick model, DNA is represented by both green line and cartoon model, $\pi-\pi$ stacking is represented by cyan dashed line and H-bond are represented by yellow dashed line.

Table S6. Count of BisQ populations in the π-stacked configuration during a 10 ns simulation period

The number of structures where the value of ω is in between -40° to -80° (both rings of BisQ are stacked) over 10 ns time scale.	BisQ FIT-PNA:RNA duplex		BisQ FIT-PNA:DNA duplex	
	Full match	G-G mismatch	Full match	G-G mismatch
[Total structures = 1000]	**111** (11.1%)	**651** (65.1%)	**186** (18.6%)	**153** (15.3%)

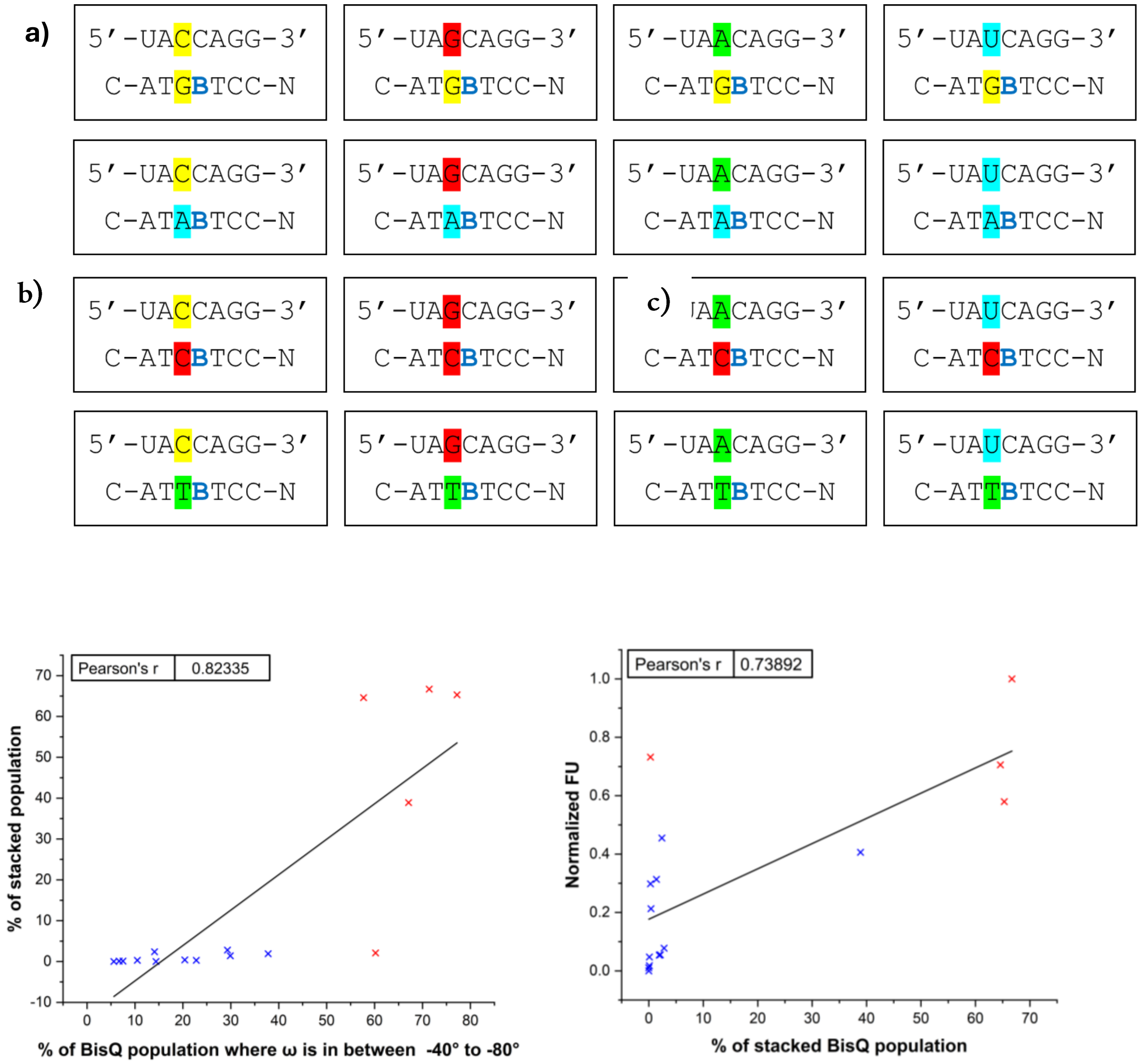

Figure S41: (a) FIT-PNA:RNA duplexes used for correlation study. (b) Correlation study between percentage of π–stacked populations vs. percantge of BisQ populations where ω is in between -40° to -80°. At 0.05 (95% confidence interval), level slope is siginificantly defferent from y=constant ($p<0.05$). Red crosses mark BisQ population with $-40^0<w<-80^0$ over 50%. (c) Corrilation study between normalised fluorescent units and percentage of well π–stacked BisQ population. At 0.05, level slope is siginificantly defferent from y=constant ($p<0.05$). Red crosses mark flourescence intensity over 50%.

References

1. Hibino M, Aiba Y, Shoji O. Cationic guanine: positively charged nucleobase with improved DNA affinity inhibits self-duplex formation. *Chem Commun*. 2020;56(17):2546-9. 10.1039/d0cc00169d
2. Alenaizan A, Barnett JL, Hud NV, Sherrill CD, Petrov AS. The proto-Nucleic Acid Builder: a software tool for constructing nucleic acid analogs. *Nucl Acids Res*. 2020;49(1):79-89. 10.1093/nar/gkaa1159
3. Li S, Olson WK, Lu X-J. Web 3DNA 2.0 for the analysis, visualization, and modeling of 3D nucleic acid structures. *Nucl Acids Res*. 2019;47(W1):W26-W34. 10.1093/nar/gkz394
4. He J, Wang J, Tao H, Xiao Y, Huang S-Y. HNADOCK: a nucleic acid docking server for modeling RNA/DNA–RNA/DNA 3D complex structures. *Nucl Acids Res*. 2019;47(W1):W35-W42. 10.1093/nar/gkz412
5. Mohamadi F, Richards NGJ, Guida WC, Liskamp R, Lipton M, Caufield C, et al. Macromodel—an integrated software system for modeling organic and bioorganic molecules using molecular mechanics. *J Comp Chem*. 1990;11(4):440-67. https://doi.org/10.1002/jcc.540110405
6. Watts KS, Dalal P, Tebben AJ, Cheney DL, Shelley JC. Macrocycle Conformational Sampling with MacroModel. *J Chem Inf Mod*. 2014;54(10):2680-96. 10.1021/ci5001696
7. Lu C, Wu C, Ghoreishi D, Chen W, Wang L, Damm W, et al. OPLS4: Improving Force Field Accuracy on Challenging Regimes of Chemical Space. *J Chem Theory Comp*. 2021;17(7):4291-300. 10.1021/acs.jctc.1c00302
